# Olfactory receptor genes and chromosome 11 structural aberrations: Players or spectators?

**DOI:** 10.1016/j.xhgg.2023.100261

**Published:** 2023-12-30

**Authors:** Serena Redaelli, Francesca Romana Grati, Viviana Tritto, Giuliana Giannuzzi, Maria Paola Recalcati, Elena Sala, Nicoletta Villa, Francesca Crosti, Gaia Roversi, Francesca Malvestiti, Valentina Zanatta, Elena Repetti, Ornella Rodeschini, Chiara Valtorta, Ilaria Catusi, Lorenza Romitti, Emanuela Martinoli, Donatella Conconi, Leda Dalprà, Marialuisa Lavitrano, Paola Riva, Angela Bentivegna

**Affiliations:** 1 School of Medicine and Surgery, University of Milano-Bicocca, 20900 Monza, Italy; 2 R&D, Cytogenetics, Molecular Genetics and Medical Genetics Unit, Toma Advanced Biomedical Assays S.p.A. (ImpactLab), 21052 Busto Arsizio, Italy; 3 Department of Medical Biotechnology and Translational Medicine, University of Milan, 20122 Milan, Italy; 4 Department of Biosciences, University of Milan, 20122 Milan, Italy; 5 IRCCS Istituto Auxologico Italiano, Medical Cytogenetics Laboratory, 20095 Cusano Milanino, Italy; 6 UC Medical Genetics, Fondazione IRCCS San Gerardo dei Tintori, 20900 Monza, Italy; 7 Pathology and Cytogenetics Laboratory, Clinical Pathology Department, Fondazione IRCCS Ca' Granda Ospedale Maggiore Policlinico, 20162 Milan, Italy

**Keywords:** structural chromosomal aberrations, olfactory receptor genes, chromosome 11, structural variants, cytogenetics, cytogenomics, chromosomal territories, chromosomal rearrangements, non-homologous end joining, NHEJ, non-allelic homologous recombination, NAHR, replication-based microhomology-mediated break-induced replication, MMBIR

## Abstract

The largest multi-gene family in metazoans is the family of olfactory receptor (OR) genes. Human ORs are organized in clusters over most chromosomes and seem to include >0.1% the human genome. Because 369 out of 856 OR genes are mapped on chromosome 11 (HSA11), we sought to determine whether they mediate structural rearrangements involving this chromosome. To this aim, we analyzed 220 specimens collected during diagnostic procedures involving structural rearrangements of chromosome 11. A total of 222 chromosomal abnormalities were included, consisting of inversions, deletions, translocations, duplications, and one insertion, detected by conventional chromosome analysis and/or fluorescence *in situ* hybridization (FISH) and array comparative genomic hybridization (array-CGH). We verified by bioinformatics and statistical approaches the occurrence of breakpoints in cytobands with or without OR genes. We found that OR genes are not involved in chromosome 11 reciprocal translocations, suggesting that different DNA motifs and mechanisms based on homology or non-homology recombination can cause chromosome 11 structural alterations. We also considered the proximity between the chromosomal territories of chromosome 11 and its partner chromosomes involved in the translocations by using the deposited Hi-C data concerning the possible occurrence of chromosome interactions. Interestingly, most of the breakpoints are located in regions highly involved in chromosome interactions. Further studies should be carried out to confirm the potential role of chromosome territories’ proximity in promoting genome structural variation, so fundamental in our understanding of the molecular basis of medical genetics and evolutionary genetics.

## Teaser

The olfactory receptor (OR) genes constitute the largest gene family in metazoans. In humans, ORs are spread on most chromosomes, mapping 40% on chromosome 11. After the analysis of 222 rearrangements of chromosome 11, ORs did not appear to be significantly involved in genomic rearrangements despite being widely present in the genome. Homologous or non-homologous recombination and chromosomal territories’ proximity can mediate chromosomal rearrangements leading to diseases’ or genomes’ evolution.

## Introduction

Structural variants represent a type of genome mutation that can be balanced or unbalanced on the basis of a possible loss or gain of a functional genomic portion. If there is an imbalance, the aberration may be embryonically lethal, or, in the best-case scenario, it may have negative effects on a child’s development or on an adult’s ability to reproduce.^1^ Structural variants are known to derive from three major mutational mechanisms: non-homologous end joining (NHEJ), non-allelic homologous recombination (NAHR), and replication-based microhomology-mediated break-induced replication (MMBIR). All three homologous (NAHR) and non-homologous/microhomologous (NHEJ and MMBIR) events are crucial for genomic DNA rearrangements and genome evolution.^1^ Genome architectural motifs, such as repeated sequences, frequently encourage structural variations both in DNA-recombination-based events and in replication processes.^2^ For several years, attention has been focused on possible chromosomal regions that contain highly homologous and repeated sequences and fragile or unstable loci, predisposing them to susceptibility to chromosomal rearrangements. Among the repeated sequences, those including the family of olfactory receptor (OR) genes stand out for their abundance, being one of the largest multi-gene families in metazoans. Human ORs were frequently found to be spread in clusters over most chromosomes, suggesting that the “olfactory subgenome” (the OR genes and their genomic environment) may include >0.1% the human genome.^3^^,^^4^ All human chromosomes, with the exception of HSA20 and HSAY,^5^^,^^6^ include OR genes, but HSA11 is by far the richest in OR genes, as they represent >10% the whole genes in chromosome 11. The HSA11 OR regions are enriched in LINE-1 retrotransposons, repetitive elements that contribute significantly to structural variation and may play a specific role in olfactory neurons’ nuclear architecture.^7^ In addition, Ou and colleagues demonstrated that the interchromosomal low-copy repeat (LCR) harboring the OR gene cluster in 11p15.4 is a novel genomic instability region that mediates the relatively common recurrent constitutional non-Robertsonian translocation t(4;11) by NAHR.^8^ Moreover, in their computationally determined genome-wide “recurrent translocation map,” some of the potential interchromosomal NAHR pairs represent OR gene repeats. Another well-known example of OR-mediated recurrent translocation is the t(4;8)(p16;p23), where heterozygous sub-microscopic inversion polymorphisms of the OR region at 8p23 play a crucial role in the generation of chromosomal imbalances through unusual meiotic exchanges.^9^

Based on this knowledge, we wondered if OR genes could trigger human chromosomal rearrangements. In particular, since 369 (43%) out of 856 OR genes are found on HSA11,^10^ in this study, we examined structural rearrangements involving this chromosome in a series of cases collected during diagnostic procedures in five laboratories. A total of 222 chromosomal abnormalities involving chromosome 11 were gathered, including translocations, inversions, deletions, duplications, and one insertion. We searched for a possible association with the presence of OR genes as mediators and/or sites of breakage causing structural rearrangements by using conventional and molecular cytogenetics and a bioinformatics-statistical approach, suggesting that OR genes are not preferentially involved.

## Subjects and methods

### General sample data

The 220 samples were collected from five medical genetics laboratories with oversight by the respective institutional review boards and after written informed consent was obtained from parents or legal guardians. A total of 222 chromosomal abnormalities were identified, because two cases carry a double rearrangement: 155 were determined by conventional chromosome analysis of the karyotype and include 138 translocations, 14 inversions, 2 deletions, and 1 insertion (Table S1); the 65 remaining cases were found using array comparative genomic hybridization (array-CGH) and include 32 duplications and 35 deletions (Table S2). All investigations were carried out following precise clinical indications, such as suspicion of fetal abnormalities reported by ultrasound analysis in prenatal cases or complex pediatric pictures or reproductive problems in adults. In any case, no statistically significant differences between the two sexes were observed (chi-squared p > 0.05; Table S3). Some examples of identified alterations are shown in Figure S1.

### Chromosome analysis

Standard methods were applied to conduct chromosome analysis by QFQ and GTG banding as previously reported.^11^ Detailed methods for fluorescence *in situ* hybridization (FISH) and array-CGH analysis are available in the supplemental methods.

### Statistics

We obtained the location of OR genes and coordinates of chromosome 11 cytogenetic bands in the hg19 reference from the UCSC genome browser (UCSC genes and chromosome band tracks, last access date: June 16, 2022). We considered as “OR cytoband” those cytobands where at least one OR gene was mapped and as “no OR cytoband” those where no OR gene was mapped. We excluded 11q23 from the “no OR cytoband” and calculated the total size (in base pairs) of cytobands in the two groups. We counted the number of translocations with chromosome 11 breakpoint in either cytoband group, excluding cases with breakpoint at 11q23, as t(11;22)(q23;q11) rearrangements are common and known to be mediated by AT-rich palindromes,^12^ and cases with breakpoint at the centromere. We also excluded translocation cases for which a lack of information about the sub-band hampered the assignment to either group. The null distributions of copy-number variation (CNV) breakpoints were generated by performing 1,000 permutations along chromosome 11, excluding gaps, by using BEDTools v.2.30.0.^13^ Statistical analyses were performed in R v.4.0.3.^14^

## Results

### Structural aberrations evidenced by conventional cytogenetic analysis

#### Interchromosomal rearrangements: Translocations

**Figure 1:**
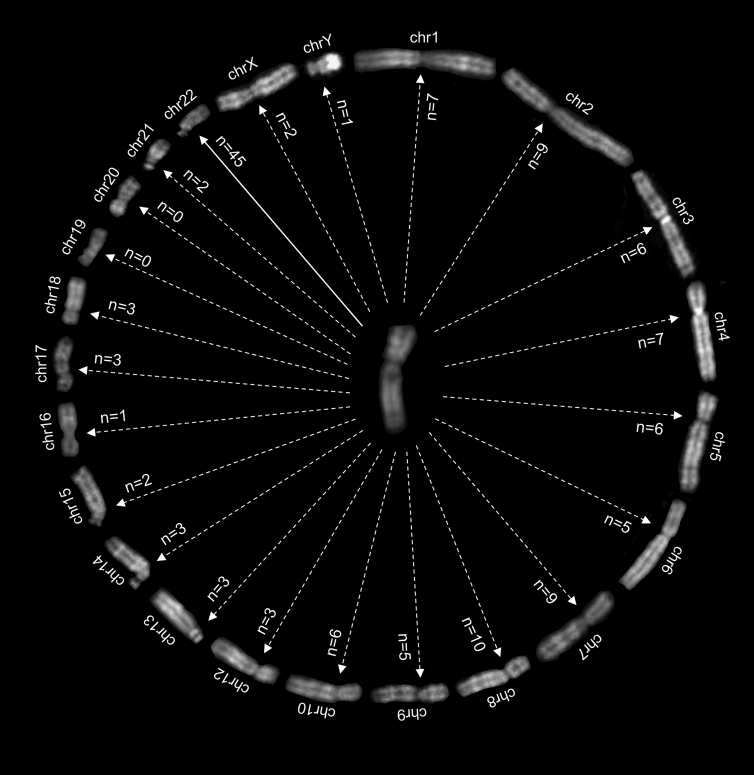
Chromosome 11 translocation predisposition Chromosomes in QFQ bands are arranged in a circle, with chromosome 11 in the middle. The arrows indicate the number of translocations collected in this specific study.

**Figure 2:**
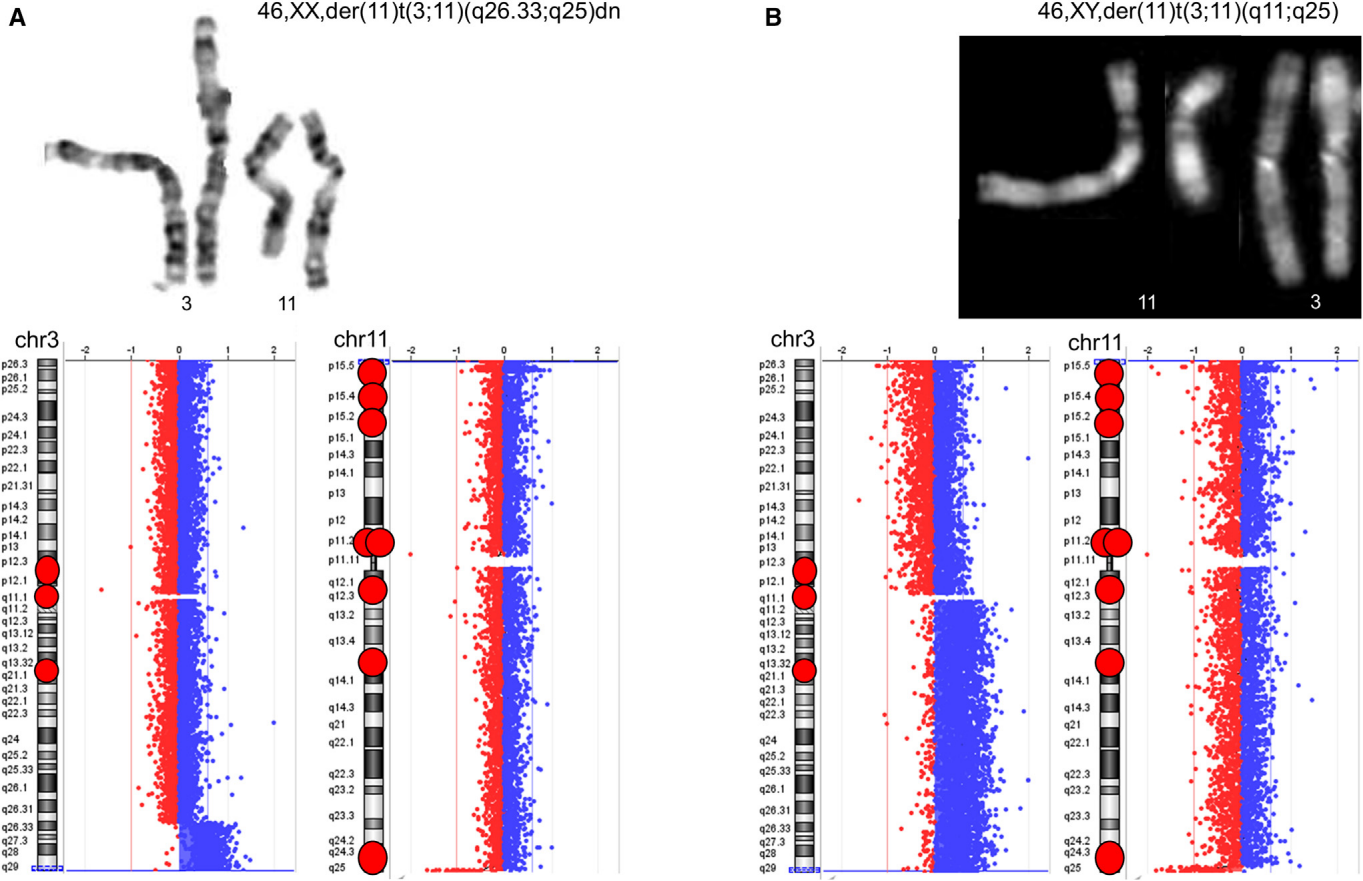
Two examples of translocation derivatives In both cases, the partner of chromosome 11 is chromosome 3. In (A), the breakpoint on chromosome 3 falls in a cytoband devoid of OR gene clusters; however, in (B), a OR gene cluster is present in the cytoband of the breakpoint of chromosome 3. The chromosome 11 breakpoints fall in the same cytoband containing OR genes, in both cases. The pairs of chromosomes (top) and their genomic profiles (bottom) are shown.

Our survey evidenced a general predisposition of chromosome 11 to rearrange with virtually all chromosomes without correlation between chromosome size and number of breakpoints (Figure 1). Notably, for some translocation partners, we identified breakpoints both in regions containing OR genes and in non-OR regions (Figure 2). Only chromosomes 19 and 20 do not show rearrangements, the latter being curiously one of the only two chromosomes without OR genes. The disproportion of the involvement of chromosome 22 compared to the other chromosomes emerged, with 45 cases of the well-known 11q;22q translocation, caused by the presence of palindromic AT-rich repeats (PATRRs) on 11q23 (PATRR11) and on 22q11 (PATRR22).^15^ Ten (22.2%) out of 45 cases with 11q;22q translocation were identified in prenatal diagnosis as unbalanced with an extra der(22), inherited from a balanced mother. Only one case, a miscarriage, presented an imbalance with a supernumerary der(11) of paternal origin and the lack of a chromosome 22 (46,XX,+der(11)t(11;22)(q23;q11)pat,−22). In another miscarriage, the translocation 11;22 was balanced, but trisomy 22 was present (47,XX,t(11;22)(q23;q11.2),+22); the parents refused further investigation.

In order to identify a possible association with the presence of OR genes as mediators and/or sites of breakage, we first classified breakpoints from a cytomorphological and a cytogenetic point of view, according to the presence or absence of OR cluster genes in the cytobands, on the basis of Glusman’s mapping^4^ (Table S4; Figure S2). Only 18.8% breakpoints fall within a cytoband with OR genes. Regarding the partner of chromosome 11 translocations, we observed no correlation between chromosome size and the number of breakpoints. Considering the breakpoints on chromosome 11, excluding 45 cases of recurrent translocation 11q;22q, about 40% fall in regions that contain OR cluster genes (Table 1; Figure 4).

**Table 1:** Distribution of the translocation breakpoints along the cytobands of chromosome 11

Chromosome 11	Cytoband	OR genes	N° breakpoints	%
p arm	15	+	15	16.1
	14	−	0	0.0
	13	−	10	10.7
	12	−	2	2.1
	11.2	+	5	5.4
	11.1	+	3	3.2
Centromere	/	/	/	/
q arm	11	+	5	5.4
	12	−	1	1.1
	13	+	7	7.5
	14	−	4	4.3
	21	−	9	9.7
	22	−	5	5.4
	23	−	15^a^	16.1
	24	+	3	3.2
	25	−	9	9.7

Based on Glusman’s mapping, OR gene families are present or absent.^4^

a 45 recurrent translocations (11q; 22q) are not included.

**Figure 3:**
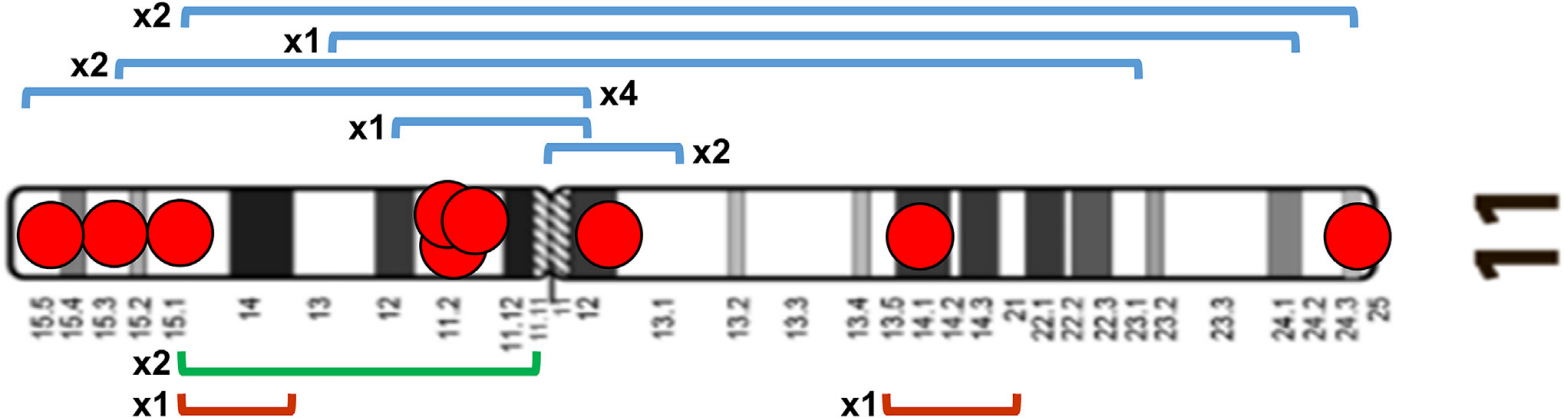
Intrachromosomal rearrangements: Inversions and deletions Ideogram of chromosome 11, showing the breakpoints of pericentric and paracentric inversions (blue and green lines, respectively) and deletions (brown lines). Cytobands containing OR genes are indicated by red circles. The numbers indicate how many times the specific anomaly has been reported in apparently unrelated subjects.

**Figure 4:**
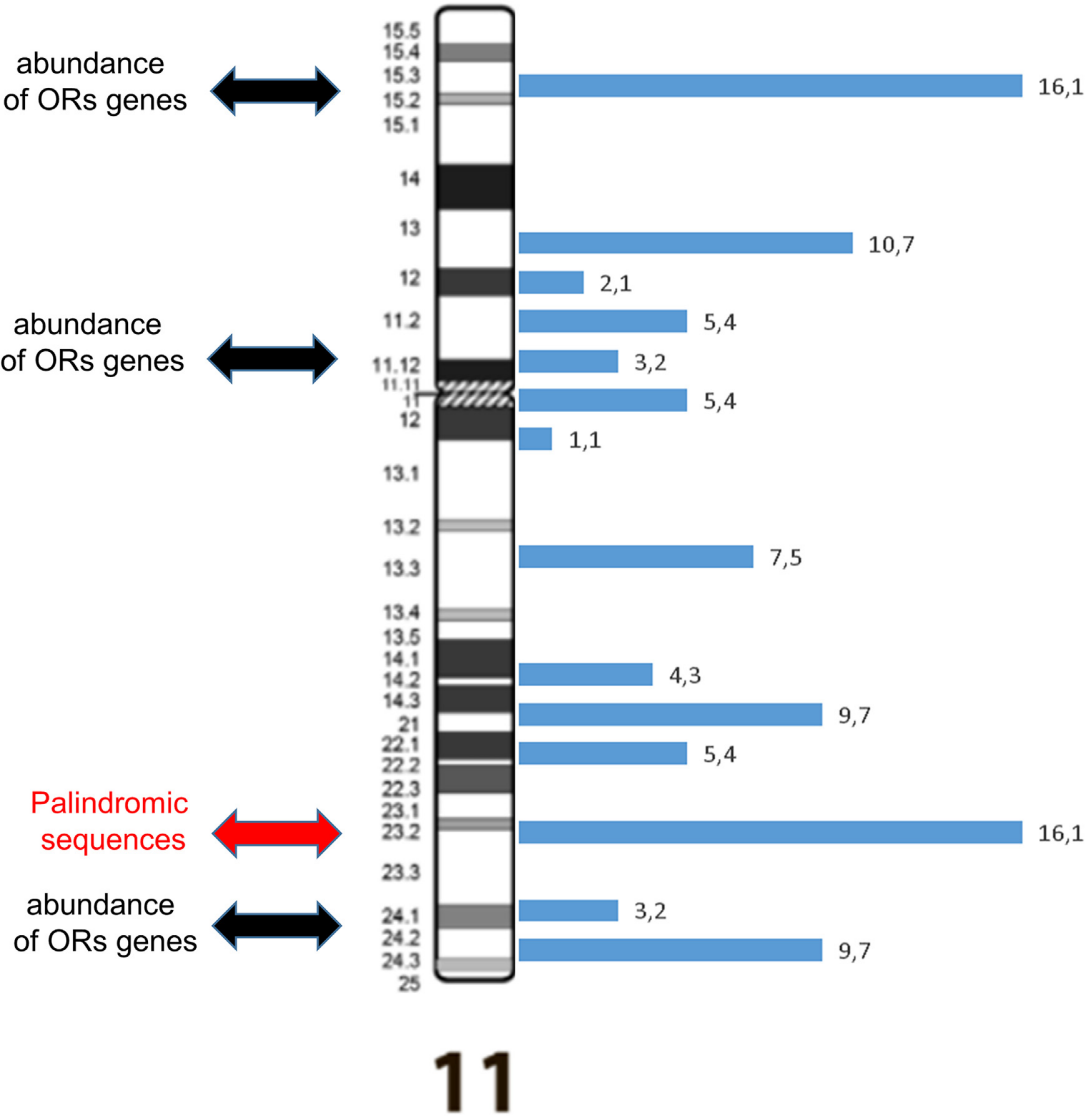
Distribution of the breakpoints on chromosome 11 The number reported on the right of the ideogram represent the percentage associated with the specific translocation from Table 1.

Then, we moved toward a more accurate statistical analysis, checking the location of OR genes by the coordinates of chromosome 11 cytogenetic bands in the hg19 reference from the UCSC genome browser. In particular, we classify those that have the chromosome 11 breakpoint located in a cytoband containing at least one OR gene from those that have the chromosome 11 breakpoints located in a cytoband without OR genes. We excluded 45 cases of recurrent translocations involving the 11q23 cytoband and also cases for which a lack of information about the sub-band hampered the assignment to either group. Our final dataset consisted of 62 translocations: 15 with chromosome 11 breakpoints mapped to an “OR cytoband” and 47 with chromosome 11 breakpoints mapped to a “no OR cytoband.” This collection did not show an enrichment of breakpoints in OR cytobands (Fisher’s exact test p = 0.14, odds ratio = 0.63, two-sided). Unfortunately, information on familiarity was available in about 50% cases (Table S1). Maternal transmission was observed to be twice as high as paternal transmission, and the rate of new mutations was not negligible. Given that physical interaction and pairing of double-strand breaks (DSBs) appear to be necessary for the inaccurate ligation of the two broken chromosomes,^16^ chromosome 11 and its translocation partners could co-localize in the normal cells’ nuclei prior to rearrangement, according to the “contact first” hypothesis that the three-dimensional genome architecture contributes to rearrangements between chromosomes showing spatial interactions.^17^ Our analysis focused on the proximity of chromosomal regions between chromosome 11 and its companion chromosomes, taking into account translocations with breakpoints in cytobands 11p15 and 11q23, which account for approximately 33% all translocations studied. By using the Hi-C data visualization software (Juicebox Aiden Lab Tool^18^) of the human GM12878 cell line, we obtained a qualitative output showing the interactions between the specific 11p15 or 11q23 region and the whole genome. Then, we selected the chromosomal partner regions involved in the translocations and the proximity for most of the cytobands was inferred (see supplemental methods, Table S5, and Figures S4A–S4Z).

#### Interchromosomal rearrangements: Insertions

The only insertion case reported in this study, 46,XX,ins(11;2)(p14;q14.3q31), was identified in the karyotype of a woman, pregnant at the 13th week of gestation, whose sister was a carrier of the same anomaly (Figure S1A). The insertion event was confirmed by FISH using 11p and 11q telomeric probes. The pregnancy was normal.

#### Intrachromosomal rearrangements: Inversions and deletions

We evidenced 14 inversions: 12 pericentric and 2 paracentric (Figure 3, blue and green lines, respectively). In contrast to translocations, in virtually all but one of the 14 diagnosed inversion cases, the breakpoints were in cytobands containing OR genes.

In this study, only 2 deletions were detectable by conventional cytogenetics (Figure 3, brown lines); one concerns the p arm, *de novo*, and one the q arm. The breakpoints do not appear to be affected by OR genes, but the numbers are too low to draw any conclusions.

### CNVs detected by array-CGH

A total of 67 CNVs were detected, of which 35 were deletions and 32 were duplications, in 65 investigated patients. As a matter of fact, in two cases, a double CNV was observed: a double duplication and a duplication followed by a deletion, both on chromosome 11 (Figure S3). We next assessed the location of the 134 breakpoints of 35 deletions and 32 duplications and found that six were within 10 kbp of an OR gene. We generated 1,000 permutations of the 134 breakpoints that showed a mean of 3.5 breakpoints within 10 kbp of an OR gene. While this value suggested a possible enrichment of breakpoints near OR genes, it did not reach statistical significance (p = 0.136). Taken together, we did not observe an enrichment of chromosome 11 rearrangement breakpoints near OR genes.

In eight cases, duplications were found together with other genomic abnormalities on other chromosomes. Also for deletions, in nine cases, they resulted in combination with a duplication and/or a further deletion in other chromosomes. Following is the description of some interesting cases.

A case of prenatal diagnosis with fetal abnormalities observed at morphological ultrasound (pregnancy from egg donation) showed a deletion: (arr[GRCh37] 11q14.1(79726995_83738395)x1) (size 4,011 kbp). Since the deletion was not present in the father, we can state that the deletion was derived from the donated egg cell. Assuming a phenotypically normal oocyte donor, we cannot attribute the pathogenicity of the fetal abnormalities to this deletion. Furthermore, in this region, no OMIM genes associated with known diseases are present.

In the double duplication case (Figure S3, case “∗”), (arr[GRCh37] 11p12p11.12(38353874_51327199)x2∼3,11q11q12.1(54829323_58291307)x2∼3), the presence of a small supernumerary marker chromosome derived from chromosome 11, in mosaic condition (40%), could only be inferred. In fact, centromeric regions are not represented by specific probes, and they cannot be visualized by array-CGH analyses. Unfortunately, patient material for FISH analysis was not available, making it impossible to test this hypothesis.

In the duplication and terminal deletion case (Figure S3, case “∗∗”), (arr[GRCh37] 11q23.3q24.1(119982356_122568533)x3,11q24.1q25(122621163_134868407)x1), a possible inverted duplication and deletion of 11qter, or inv dup del(11qter) has been assumed, but similarly to the case described above, it was not possible to confirm.

A deletion of 1,530 kbp in 11q25 (from nt133403830 to nt134934196), in addition to a duplication of 16 Mb in 5q34q35.3, was observed in two brothers with a clinical phenotype. A possible unbalanced translocation inherited from one parent could explain the same CNVs observed in both brothers. Due to lack of subject material, it was not possible to continue the study.

Finally, in a 1-year-old girl with a clinical phenotype and normal standard karyotype (46,XX), array-CGH evidenced a pathological outcome with a duplication on chromosome 3 and a deletion on chromosome 11: arr[GRCh37] 3q26.33q29(179498992_197861598)x3,11q25(134446101_134934196)x1. This is a terminal deletion on chromosome 11, without gene involvement.

## Discussion

The identification of DNA motifs and molecular mechanisms mediating structural rearrangements in the human genome is challenging. Such identification is key for the prediction of genome breakage hot spots and structural variants and the development of targeted molecular diagnostics.^2^ It is interesting to note that gene family regions and loci with genes or pseudogenes in “linked proximity” appear to be particularly prone to genomic instability, where LCR clusters might function as NAHR substrates. On the other hand, Alu elements can mediate template switching during microhomology replication-based repair mechanisms.^19^ In both cases, rearrangements arise, generating CNV alleles constituted by deletions and duplications.^1^^,^^8^^,^^20^^,^^21^ Given that chromosome 11 contains more than 50% all OR clusters (many of which dispersed throughout the human genome) and that recurrent reciprocal translocations like t(4;11) and t(8;12) are mediated by NAHR with an involvement of OR clusters (in particular for t(4;11)^8^), we wondered if OR clusters mediate chromosome 11 translocations. This idea, however, is not supported by statistical analysis that compares the presence of breakpoints in cytobands with or without OR, as well as analysis that compares breakpoint regions with or without OR sequences.

Interestingly, chromosome 11 synteny is highly conserved throughout mammalian evolution. In both primate and boreoeutherian mammalian ancestors, this chromosome was probably telocentric or acrocentric, with the centromere located at the orthologous human 11qter position. The current structure of human chromosome 11 was defined by a significant pericentric inversion and centromere repositioning event that occurred in the common ancestor of humans and African apes.^22^ The two inversion breakpoints were found at 11q13.4 and 11p15.4, the latter about 1 Mbp (million base pairs) from the 11p15.4 OR gene cluster. Interestingly, two OR genes (*OR7E12P* and *OR7E117P*) are located at the 11p15.4 inversion breakpoint (Gencode v.44). Similarly, *OR7E87P* and *OR7E4P* and *OR7E126P* and *OR7E128P* genes are located at the 11q13.4 breakpoint interval. The absence of orthologs at corresponding locations in orangutan and rhesus genomes suggests that these genes were absent in the common ape ancestor. Conversely, their presence in the chimpanzee, bonobo, and/or gorilla genomes suggests that these genes were inserted at these locations in the common ancestor of humans and African apes. However, we cannot discern whether they triggered this evolutionary rearrangement or not.

Our results are consistent with the hypothesis proposed by Chiang et al.^23^ that canonical NHEJ (c-NHEJ) is the main mechanism at the basis of balanced translocations. Replication-based mechanisms such as fork stalling and template switching (FoSTeS) and MMBIR are mechanisms possibly underlying the formation of non-recurrent structural variants in humans associated with the onset of many diseases.^2^^,^^24^^,^^25^ On average, about one DSB per 10^8^ bp occurs spontaneously in the genome of normal human cells, and if the repair by HR does not occur, the mechanism mainly involved in the joining of chromosome ends seems to be NHEJ.^26^^,^^27^

The visualization of the proximity between the cytobands most involved in the translocations suggests their territorial proximity. Even if we obtained only qualitative evidence, we speculate that when a break occurs, the close proximity of chromosomal territories could lead the DNA repair mechanisms generating the observed translocations. In fact, most of the cytobands involved in the translocations seem to localize in close chromosome territories. Conversely, the most common recurrent constitutional translocation, t(11;22)(q23;q11), does not show this kind of proximity of chromosome territories (Figure S4Z). For this type of aberration, the supported model would be an increased rate of cruciform structures at PATRR regions that leads to increased DSB-mediated repair via the NHEJ pathway.^15^

In conclusion, this is a retrospective, multicenter work with a large collection of specific cases on rearrangements involving chromosome 11, including translocations, inversions, deletions, duplications, and one insertion. There is no similar work in the literature, to our knowledge, with such a large collection of cases. We focused more on translocations because they are more represented in our survey and therefore also assessable from a statistical point of view. Although OR genes have been implicated in t(4;11) and t(8;12),^8^ our analyses indicate that OR genes are not preferentially involved in the reciprocal translocation of chromosome 11 and its partner chromosomes. Chromosome 11 structural alterations appears to be caused by a variety of DNA motifs and mechanisms, some of which are still understudied, such as those induced by the closeness of particular chromosomal territories. Further studies aimed at sequencing breakpoint translocations should be carried out to understand the nature of the sequences involved, improve knowledge on mechanisms causing genome structural variations and possible environmental effects increasing susceptibility to genetic diseases, and provide new insights into genome evolution.

## Web Resources

Juicebox Aiden Lab Tool, http://www.aidenlab.org/juicebox/.

## Supplemental information

Document S1. Figures S1–S4, Tables S1–S5, and supplemental methods

Document S2. Article plus supplemental information
