# Supplementary material for: Olfactory receptor genes and chromosome 11 structural aberrations: Players or spectators?
Source: HGG Adv. 2023 Dec 30;5(2):100261. doi: 10.1016/j.xhgg.2023.100261 (PMC10820794; doi:10.1016/j.xhgg.2023.100261)
Supplement: Document S1. Figures S1–S4, Tables S1–S5, and supplemental methods [file mmc1.pdf]

HGGA, Volume *5*

Supplemental information

Olfactory receptor genes and chromosome 11 structural aberrations: Players or spectators?

Serena Redaelli, Francesca Romana Grati, Viviana Tritto, Giuliana Giannuzzi, Maria Paola Recalcati, Elena Sala, Nicoletta Villa, Francesca Crosti, Gaia Roversi, Francesca Malvestiti, Valentina Zanatta, Elena Repetti, Ornella Rodeschini, Chiara Valtorta, Ilaria Catusi, Lorenza Romitti, Emanuela Martinoli, Donatella Conconi, Leda Dalprà, Marialuisa Lavitrano, Paola Riva, and Angela Bentivegna

Supplemental Information

Table of contents

Figure S1 (A-F). Examples of identified alterations in this work.

Figure S2 (A-G). Breakpoint distribution of chromosome translocations in relation to OR gene cluster locations.

Figure S3. Copy number variations detected by array CGH.

Figure S4. Hi-C heat maps showing inter-chromosomal interactions between chromosome 11 and its partner chromosomes in translocations.

Table S1. Sex distribution of patients showing chromosome 11 anomalies identified by conventional chromosome analysis.

Table S2. Sex distribution of patients showing chromosome 11 anomalies identified by array-CGH.

Table S3. Inheritance of translocation.

Table S4. Distribution of translocation breakpoints on chromosome 11 partners.

Table S5. Cytobands involved in the main collected translocations, visualized on the Hi-C maps.

Supplemental Methods.

1) Array Comparative Genomic Hybridization (Array-CGH).

2) Juicebox Aiden Lab Tool.

Figure S1. Examples of identified alterations in this work.

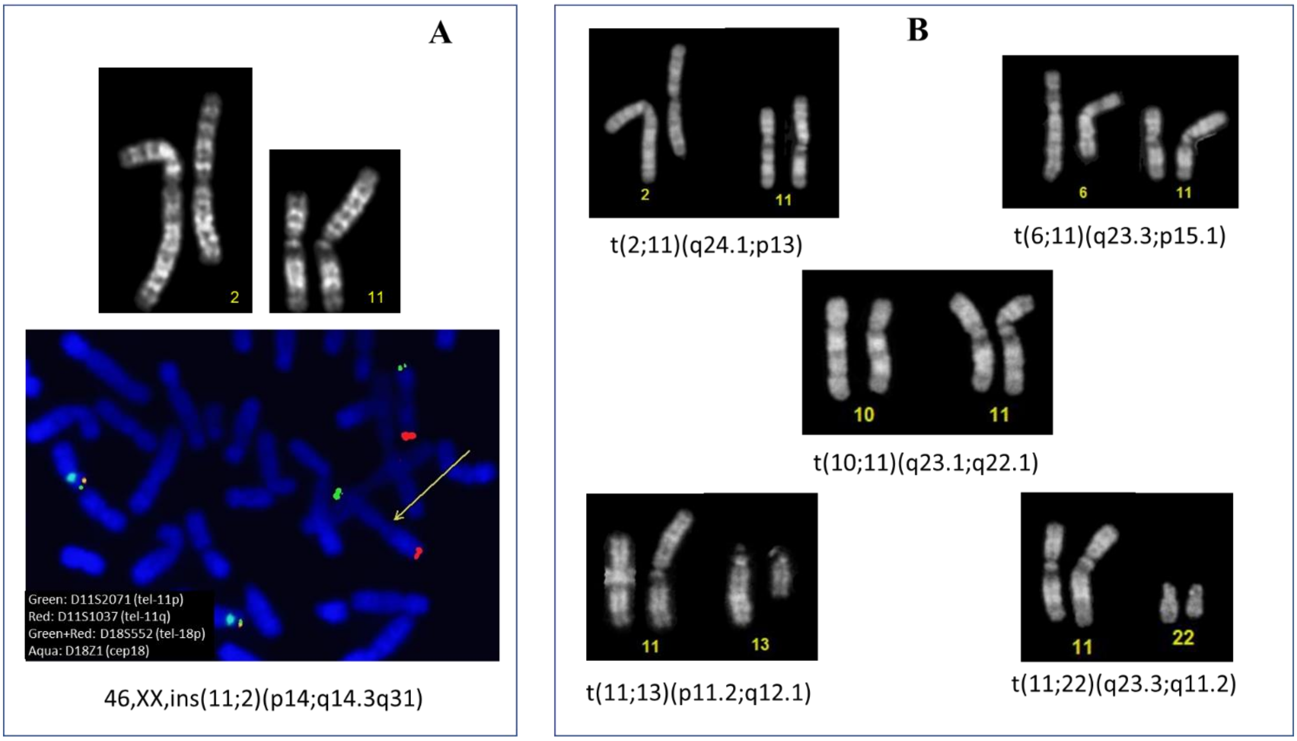

Figure S1. A. The unique insertion case: part of the long arm of chromosome 2 is inserted into the short arm of chromosome 11. Top: QFQ banded chromosomes 2 and 11 (normal ones on the left of each couple). Bottom: FISH with Vysis ToTelVysion Multi-Color FISH Probe Kit. Probes that recognize the specific telomeric regions of 11 (green= tel 11p; red= tel 11q) hybridize in the correct position, as can be observed in the partial metaphase. The arrow indicates the inserted chromosome. (red+green= tel 18p; aqua=CEP18). FISH analysis was performed with chromosome 11 probes and not with the whole kit.

Figure S1. B. QFQ banded chromosomes from cases of translocation involving chromosome 11 identified in this collection. The abnormal chromosomes are on the right of each couple.

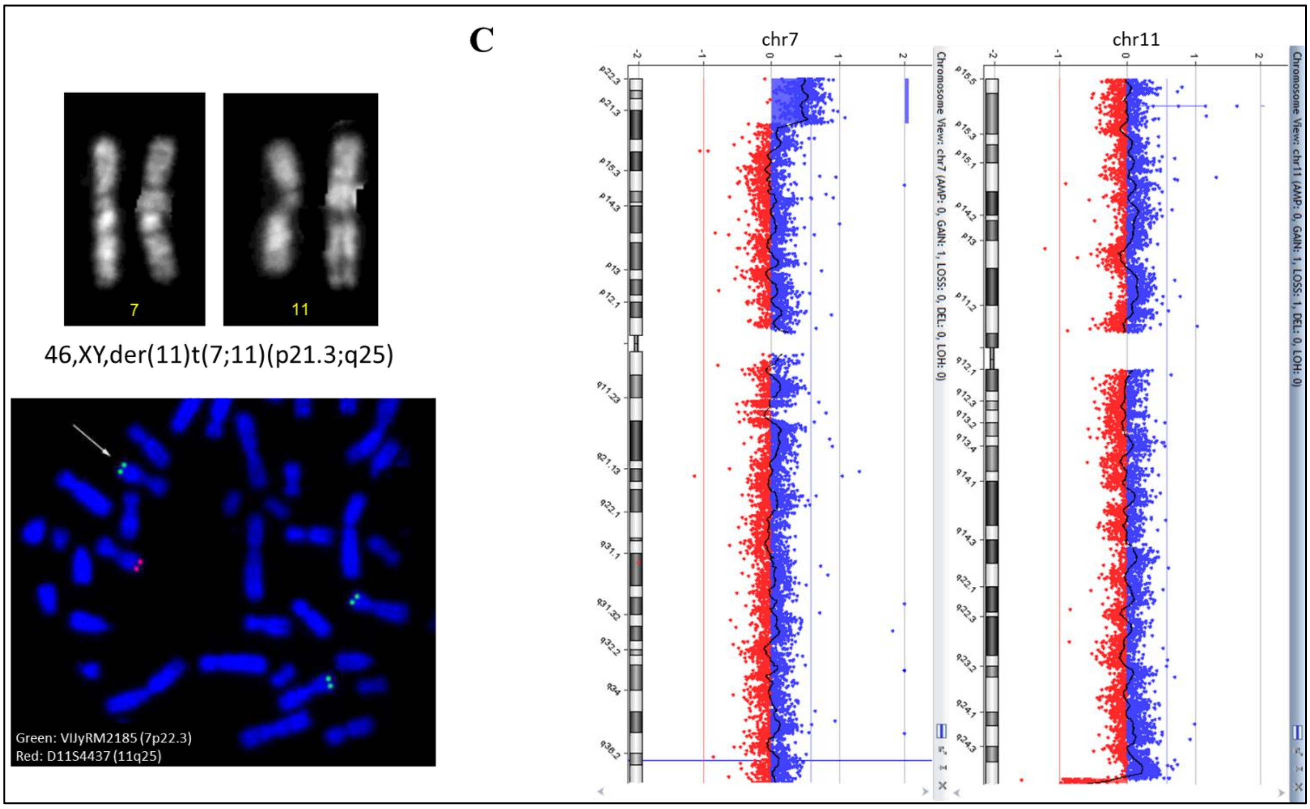

Figure S1. C. A derivative of translocation 7;11 evidenced by QFQ banding, FISH, and array-CGH. Top left: QFQ banded chromosomes with the derivative 7 on the right and the derivative chromosome 11 on the left, respectively. Bottom left: the partial metaphase shows FISH with a specific probe for 7p telomere, (7p22.3, VIJyRM2185, green) and a specific probe for 11q telomere, (11q25, D11S4437, red). Three green signals are observed, of which two are correctly positioned in telomeres 7p and one positioned on the derivative 11q (arrow). Normal chromosome 11 shows a 11q red signal on the qter. Right: array-CGH chromosome view of the two chromosomes involved in the translocation.

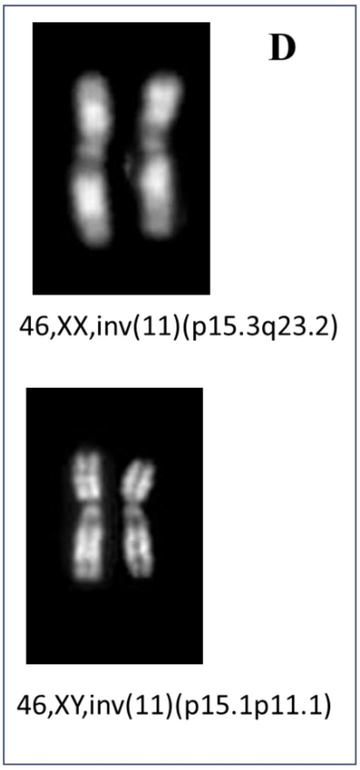

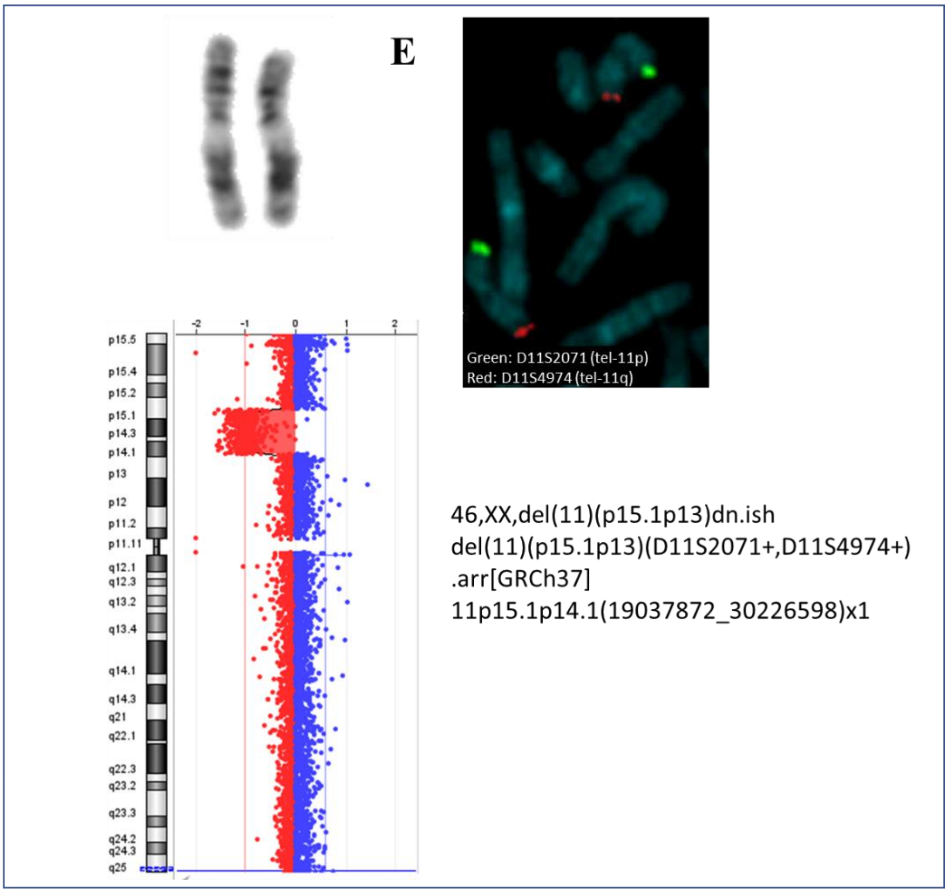

Figure S1. D. Top: a pericentric inversion observed in QFQ bands (the abnormal chromosome 11 is on the right of the couple); bottom: a paracentric inversion in QFQ bands (the abnormal chromosome 11 is on the left of the couple).

Figure S1. E. A 11p interstitial deletion identified by conventional cytogenetics (top) and confirmed by array-CGH (bottom). On the left: Chromosomes 11 are shown in GTG bands (the abnormal chromosome 11 is on the right of the couple). On the right: FISH with 11 specific telomeric probes shows normal signals (tel-11p, D11S2071, green; tel-11q, D11S4974, red).

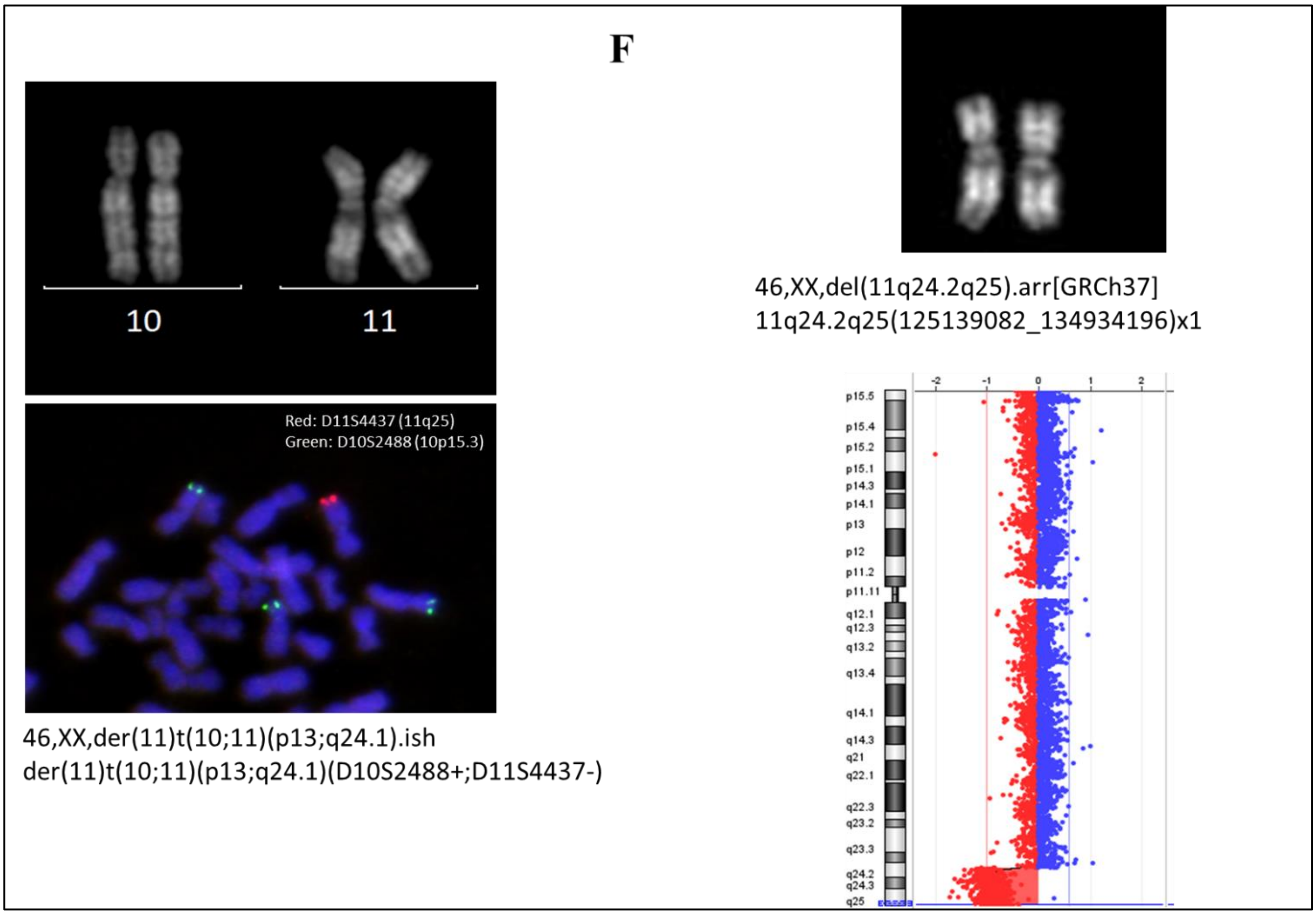

Figure S1. F. Two examples of chromosome alterations not detected by conventional cytogenetics. QFQ banding of chromosomes do not show any alterations in both cases (top panels). On the left, an unbalanced translocation with a derivative identified by array-CGH and confirmed by FISH. The bottom left panel shows a partial metaphase with three green signals (10p15.3, D10S2488) and one red signal (11q25, D11S4437), confirming the imbalance of the translocation. On the right, a terminal deletion identified by array-CGH (bottom).

Figure S2. Breakpoint distribution of chromosome translocations in relation to OR gene cluster locations.

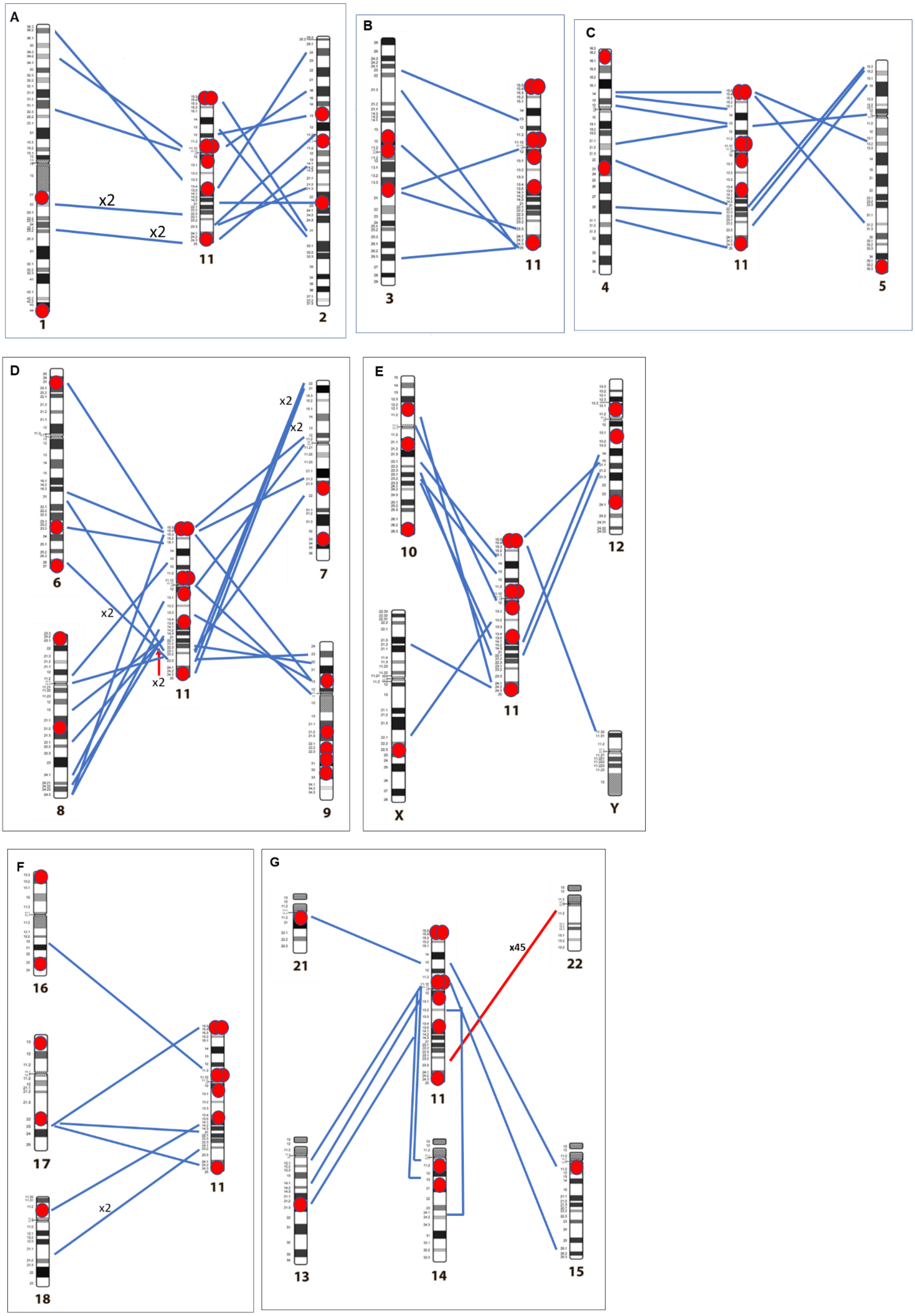

Figure S2. A-G. Breakpoint distribution of chromosome translocations in relation to OR gene cluster locations based on Glusman's mapping (red circles; see ref. 4 in the main text). Blue lines indicate bands involved in translocations with chromosome 11; the recurrent 11q;22q translocation is represented by a red line. "x N°" (for example x2): indicates the number of times the rearrangement is identified. See Table S4 for details.

Figure S3. Copy number variations detected by array CGH.

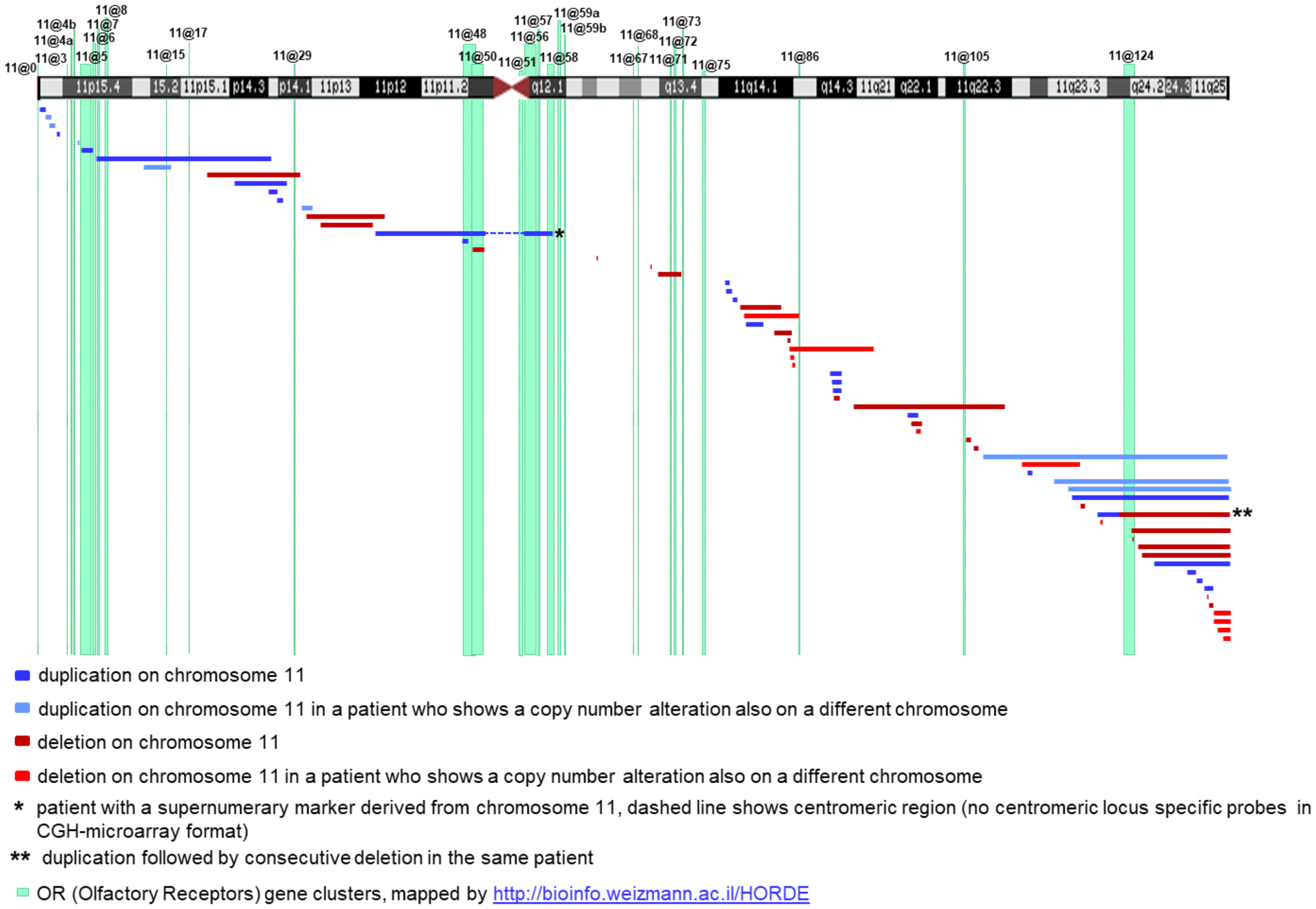

Figure S3. Copy number variations detected by array CGH. 11@N stands for OR gene cluster on chromosome 11 and N is the position in Mbp.

Figure S4. Hi-C heat maps showing inter-chromosomal interactions between chromosome 11 and its partner chromosomes in translocations. **(a)** chr11 vs chr2; **(b)** chr11 vs chr4; **(c)** chr11 vs chr5; **(d)** chr11 vs chr5; **(e)** chr11 vs chr6; **(f)** chr11 vs chr6; **(g)** chr11 vs chr7; **(h)** chr11 vs chr7; **(i)** chr11 vs chr8; **(j)** chr11 vs chr9; **(k)** chr11 vs chr12; **(l)** chr11 vs chr17; **(m)** chr11 vs chrY; **(n)** chr11 vs chr1; **(o)** chr11 vs chr2; **(p)** chr11 vs chr2; **(q)** chr11 vs chr3; **(r)** chr11 vs chr5; **(s)** chr11 vs chr6; **(t)** chr11 vs chr6; **(u)** chr11 vs chr7; **(v)** chr11 vs chr7; **(w)** chr11 vs chr8; **(x)** chr11 vs chr9; **(y)** chr11 vs chr18; **(z)** chr11 vs chr22. Each panel obtained using Juicebox Aiden Lab Tool (http://www.aidenlab.org/juicebox/) shows the selected Hi-C dataset, the cell line on which the experiment was carried out (GM12878), the resolution (250 or 500 kb), and the genomic coordinates of the two chromosomes displayed. On the x-axis is shown one of the two partner chromosomes, on the y-axis the other one. The color scale from white to red indicates an increasing number of inter-chromosomal interactions. The maximum value of interactions detected in each investigated chromosome pair is shown and graphically represented by red. The map region showing the interactions between the two cytobands containing translocation breakpoints is boxed in black, and the respective chromosomal coordinates are reported.

a

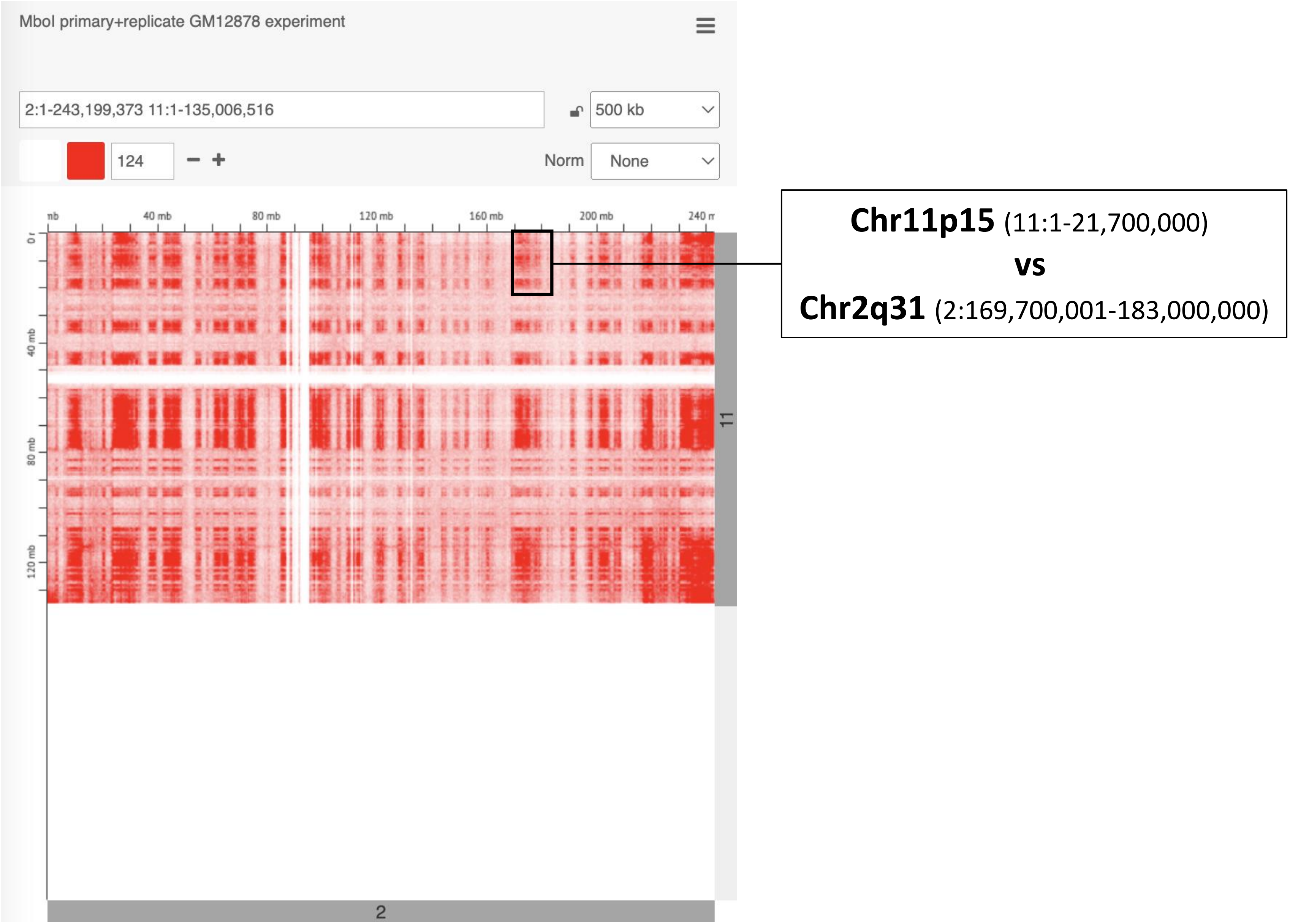

MboI primary+replicate GM12878 experiment
2:1-243,199,373 11:1-135,006,516
500 kb
124
Norm
None
40 mb
80 mb
120 mb
160 mb
200 mb
11
2
Chr11p15 (11:1-21,700,000)
vs
Chr2q31 (2:169,700,001-183,000,000)

b

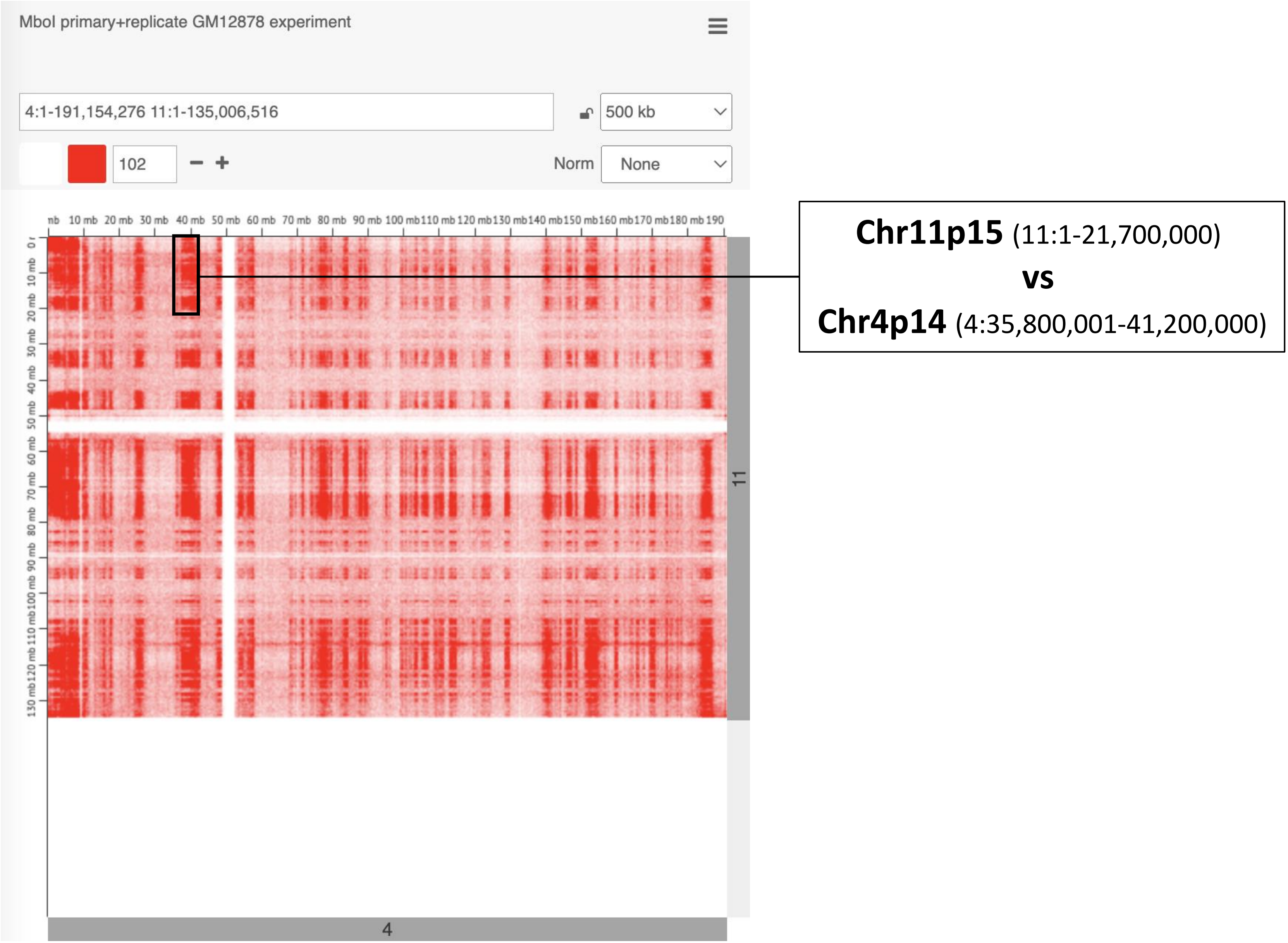

MboI primary+replicate GM12878 experiment
4:1-191,154,276 11:1-135,006,516
500 kb
102
Norm
None
11
4
Chr11p15 (11:1-21,700,000)
vs
Chr4p14 (4:35,800,001-41,200,000)

c

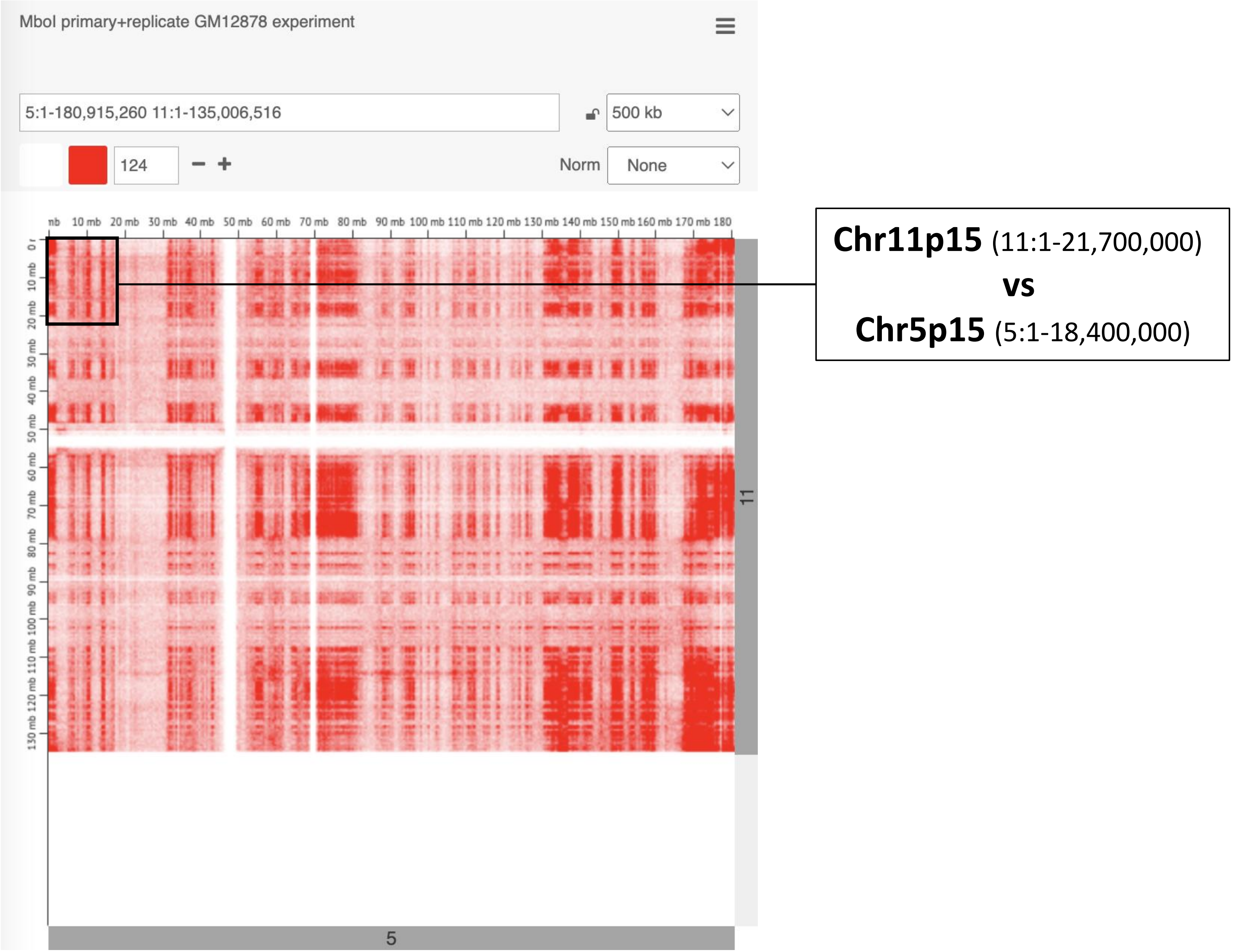

MboI primary+replicate GM12878 experiment
5:1-180,915,260 11:1-135,006,516
500 kb
124
Norm
None
11
5
Chr11p15 (11:1-21,700,000)
vs
Chr5p15 (5:1-18,400,000)

d

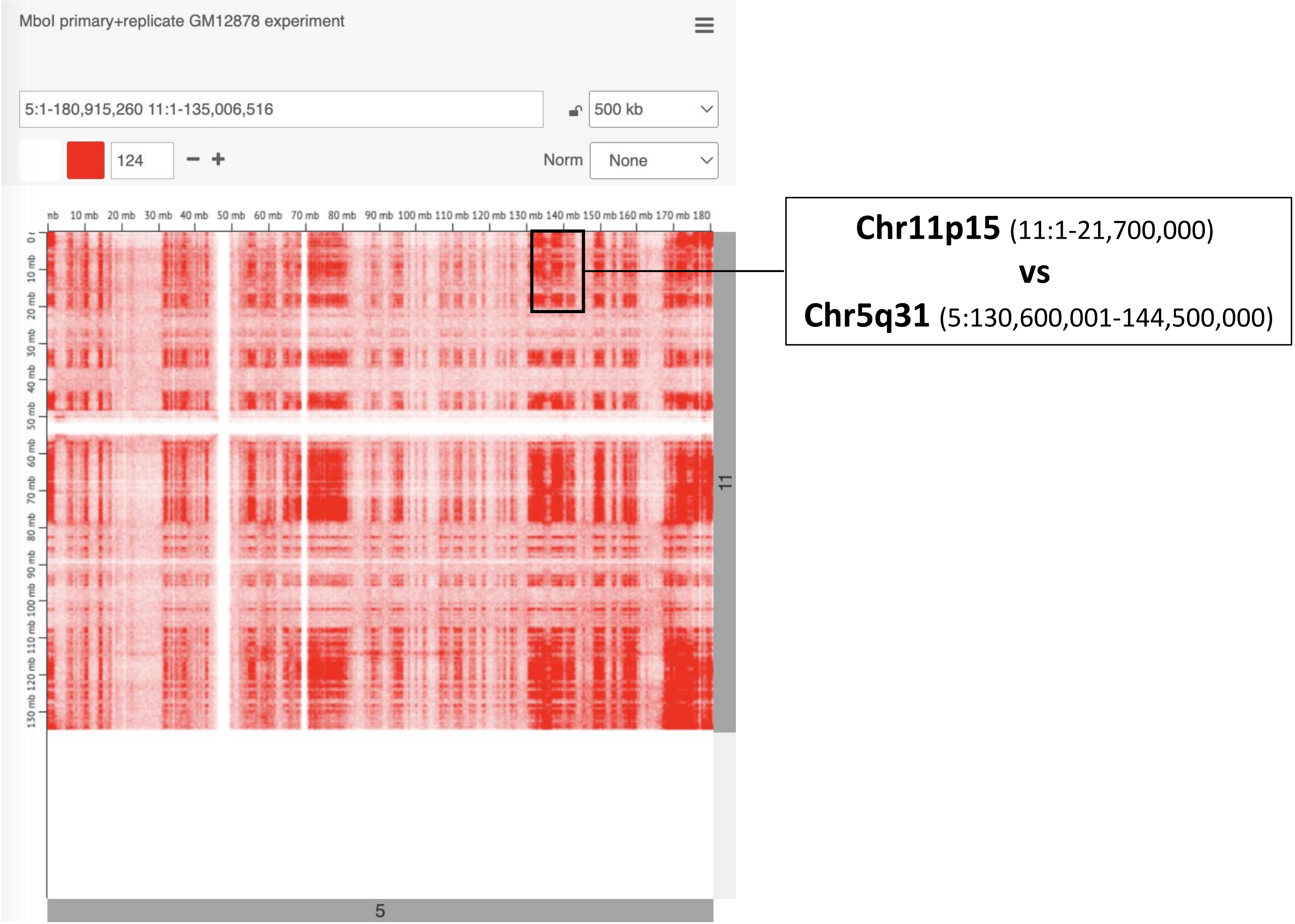

MboI primary+replicate GM12878 experiment
5:1-180,915,260 11:1-135,006,516
500 kb
124
Norm
None
Chr11p15 (11:1-21,700,000)
vs
Chr5q31 (5:130,600,001-144,500,000)
11
5

e

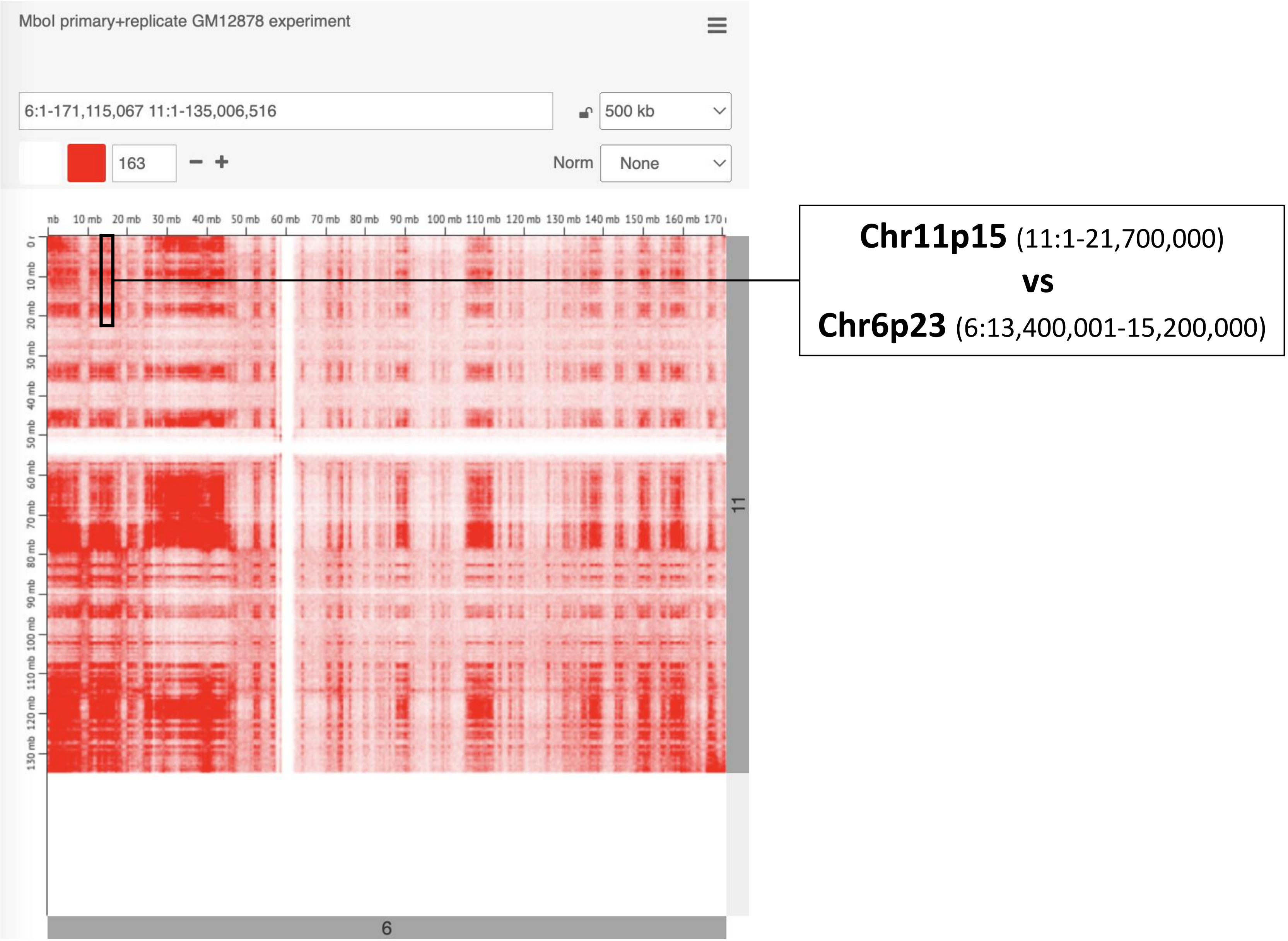
MboI primary+replicate GM12878 experiment
6:1-171,115,067 11:1-135,006,516
500 kb
163
Norm
None
10 mb 20 mb 30 mb 40 mb 50 mb 60 mb 70 mb 80 mb 90 mb 100 mb 110 mb 120 mb 130 mb 140 mb 150 mb 160 mb
11
6
Chr11p15 (11:1-21,700,000)
vs
Chr6p23 (6:13,400,001-15,200,000)

f

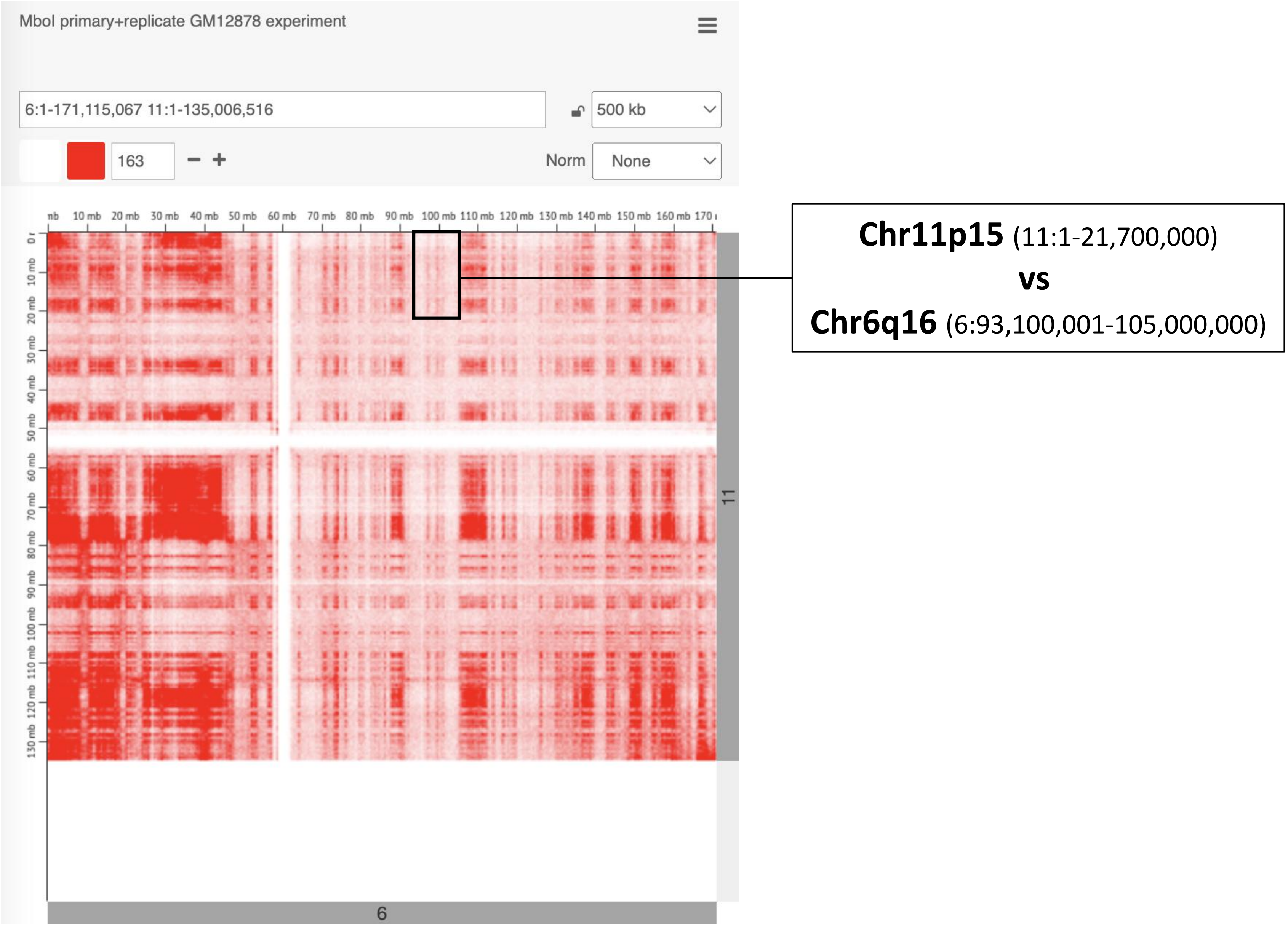
MboI primary+replicate GM12878 experiment
6:1-171,115,067 11:1-135,006,516
500 kb
163
Norm
None
11
6
Chr11p15 (11:1-21,700,000)
vs
Chr6q16 (6:93,100,001-105,000,000)

g

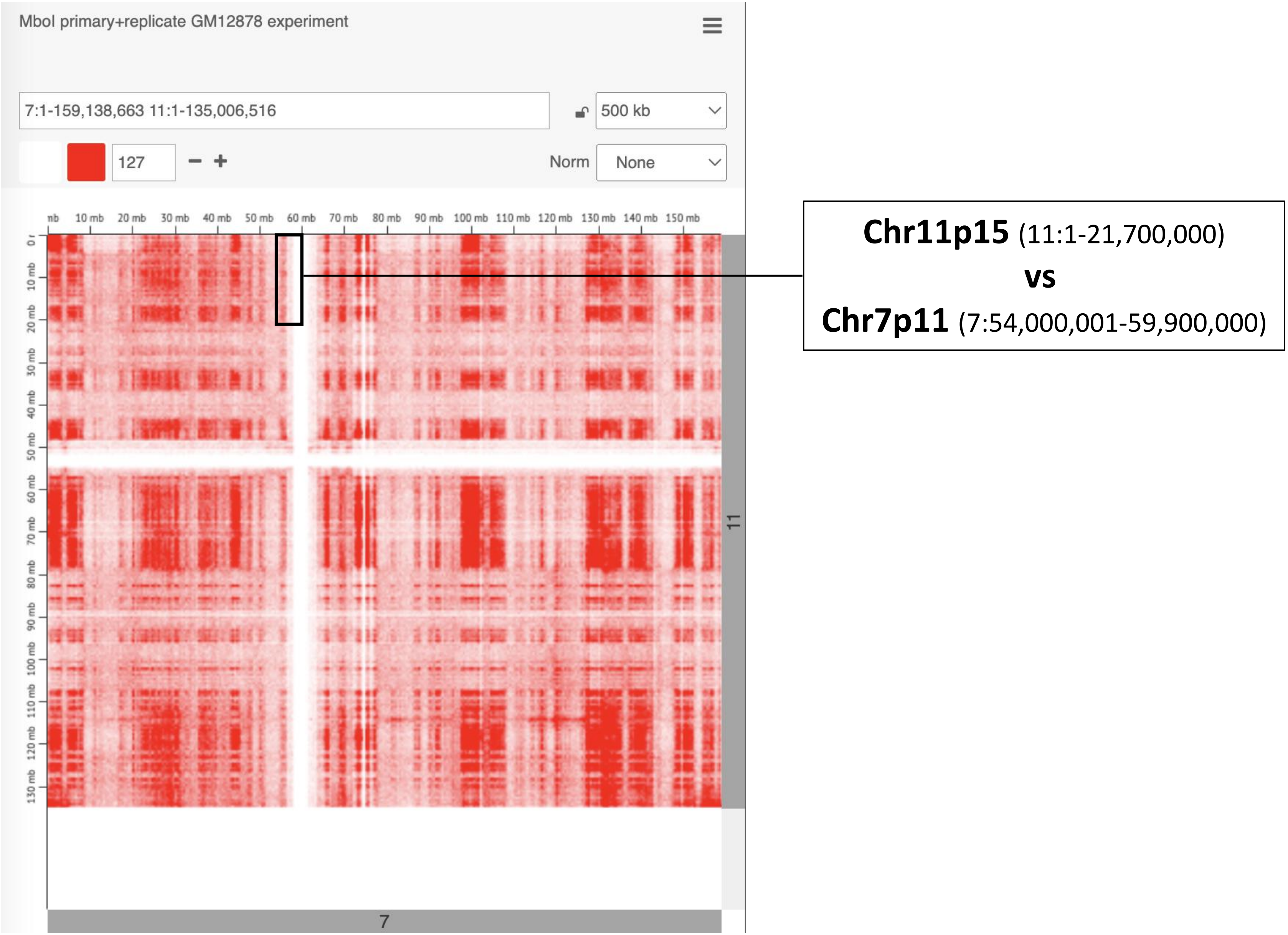
MboI primary+replicate GM12878 experiment
7:1-159,138,663 11:1-135,006,516
500 kb
127
Norm
None
10 mb
20 mb
30 mb
40 mb
50 mb
60 mb
70 mb
80 mb
90 mb
100 mb
110 mb
120 mb
130 mb
140 mb
150 mb
11
7
Chr11p15 (11:1-21,700,000)
vs
Chr7p11 (7:54,000,001-59,900,000)

h

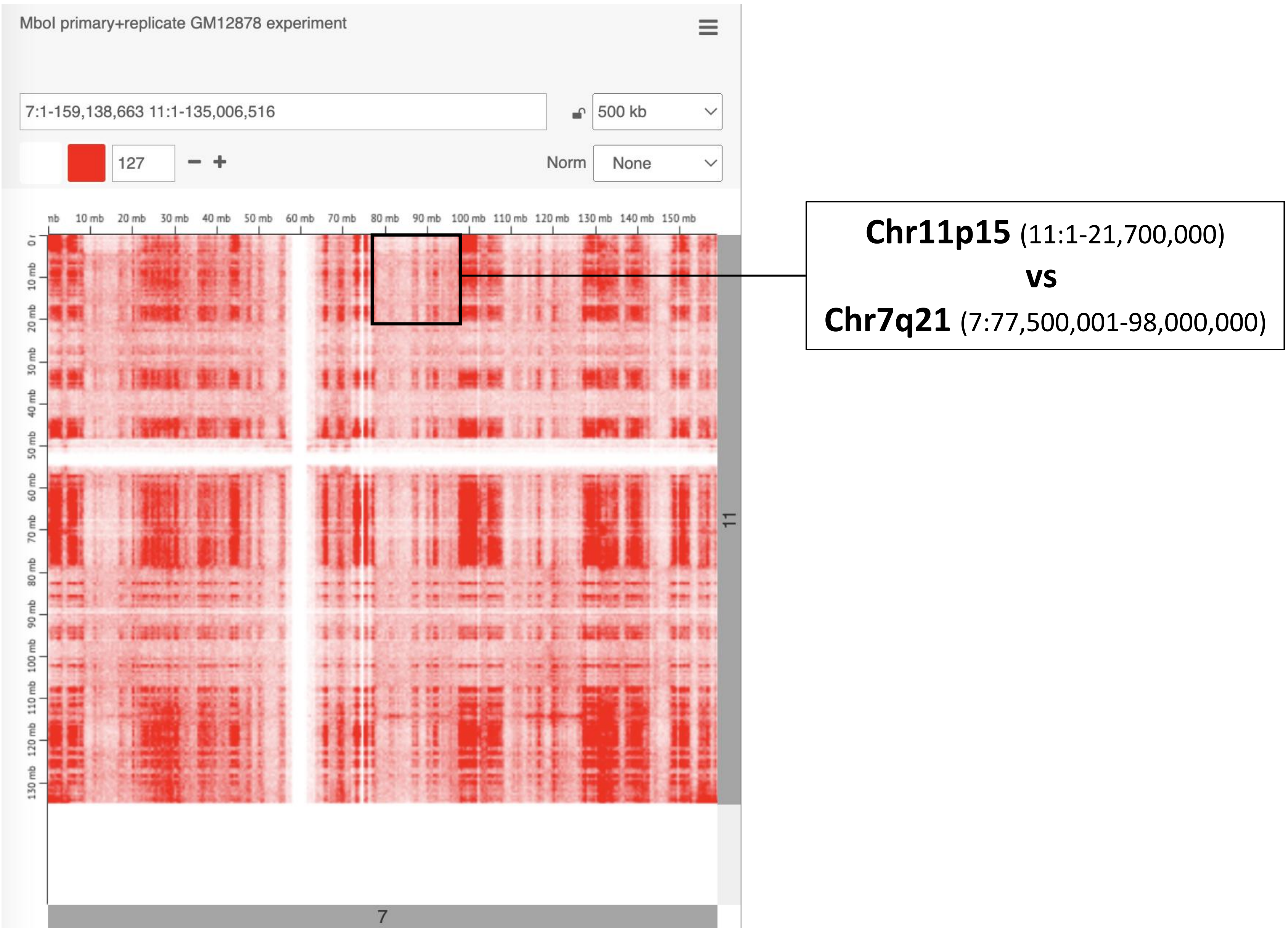
MboI primary+replicate GM12878 experiment
7:1-159,138,663 11:1-135,006,516
500 kb
127
Norm
None
10 mb 20 mb 30 mb 40 mb 50 mb 60 mb 70 mb 80 mb 90 mb 100 mb 110 mb 120 mb 130 mb 140 mb 150 mb
11
7
Chr11p15 (11:1-21,700,000)
vs
Chr7q21 (7:77,500,001-98,000,000)

i

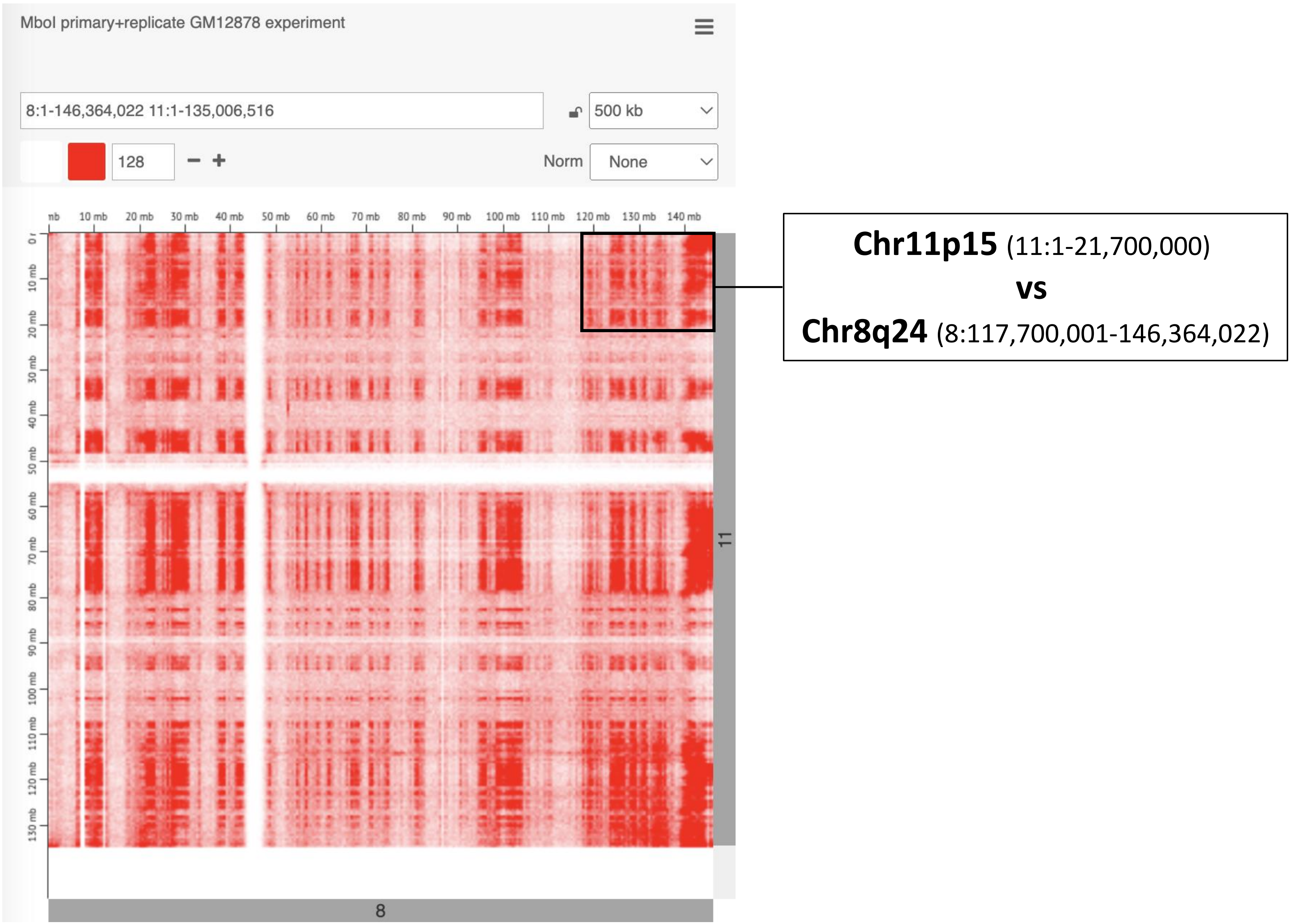
MboI primary+replicate GM12878 experiment
8:1-146,364,022 11:1-135,006,516
500 kb
128
Norm
None
10 mb
20 mb
30 mb
40 mb
50 mb
60 mb
70 mb
80 mb
90 mb
100 mb
110 mb
120 mb
130 mb
140 mb
11
8
Chr11p15 (11:1-21,700,000)
vs
Chr8q24 (8:117,700,001-146,364,022)

j

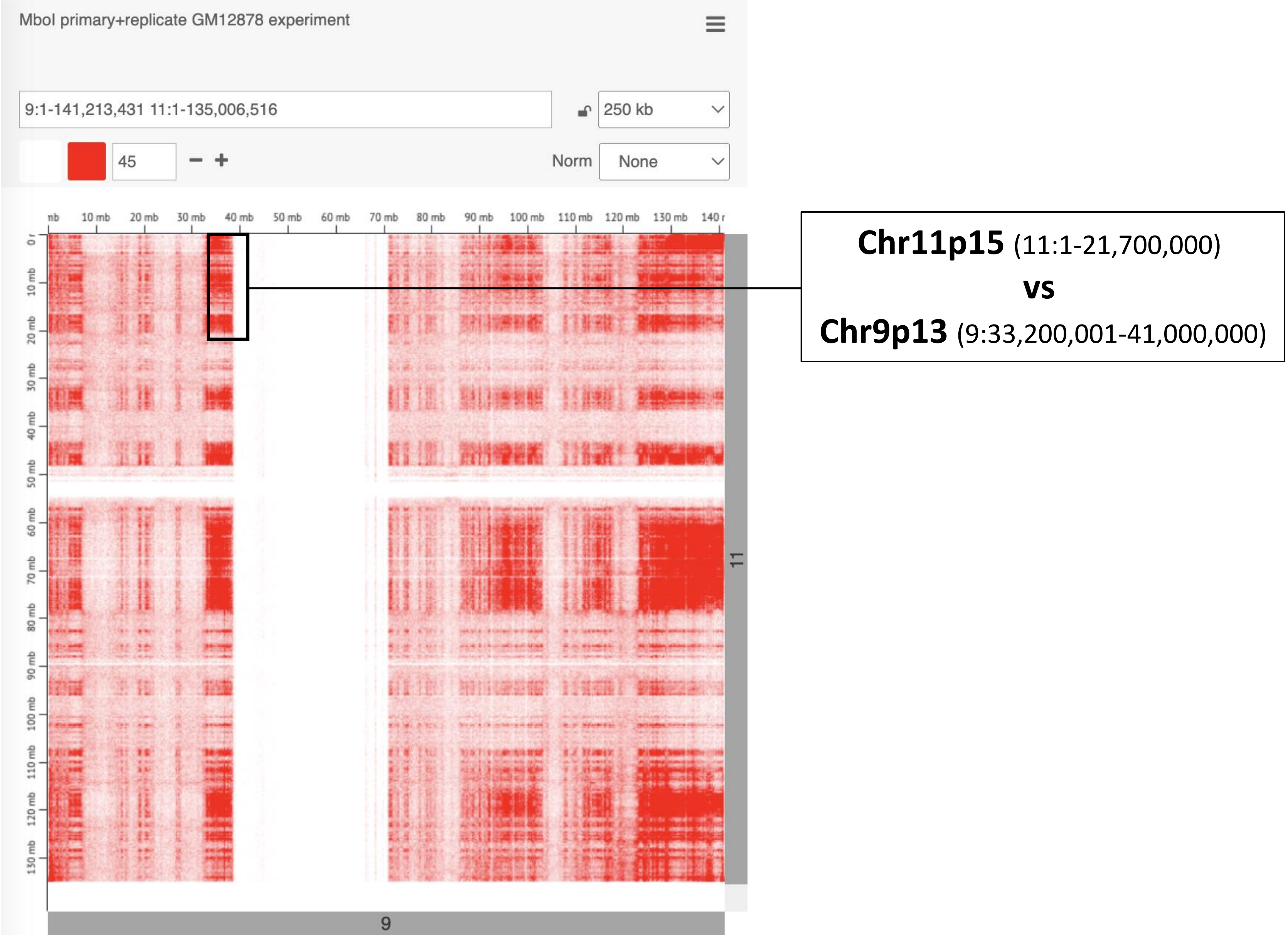

MboI primary+replicate GM12878 experiment
9:1-141,213,431 11:1-135,006,516
250 kb
45
Norm
None
10 mb
20 mb
30 mb
40 mb
50 mb
60 mb
70 mb
80 mb
90 mb
100 mb
110 mb
120 mb
130 mb
9
11
Chr11p15 (11:1-21,700,000)
vs
Chr9p13 (9:33,200,001-41,000,000)

k

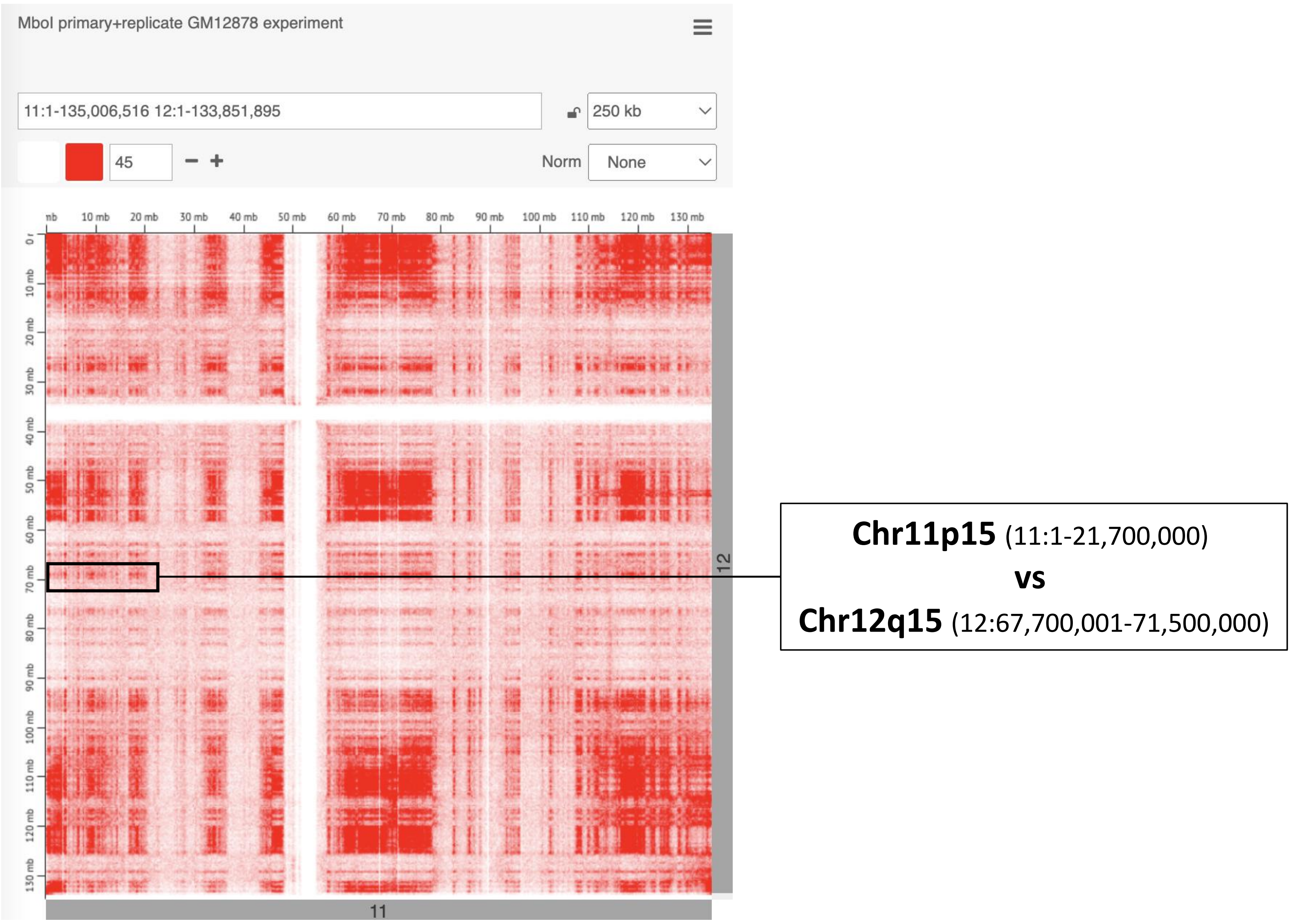

Mbol primary+replicate GM12878 experiment
11:1-135,006,516 12:1-133,851,895
250 kb
45
Norm
None
10 mb
20 mb
30 mb
40 mb
50 mb
60 mb
70 mb
80 mb
90 mb
100 mb
110 mb
120 mb
130 mb
12
11
Chr11p15 (11:1-21,700,000)
vs
Chr12q15 (12:67,700,001-71,500,000)

I

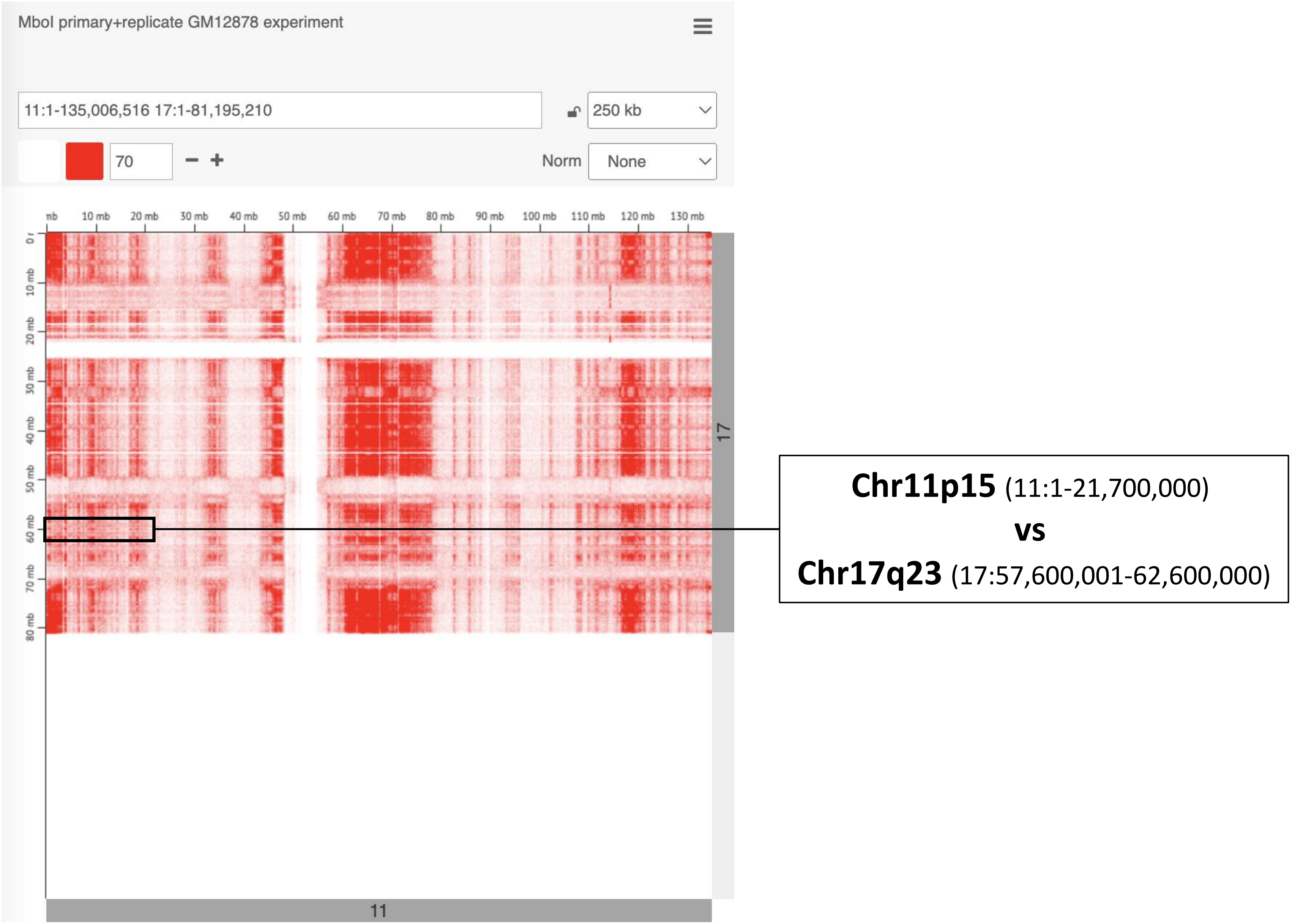
MboI primary+replicate GM12878 experiment
11:1-135,006,516 17:1-81,195,210
250 kb
70
Norm
None
10 mb
20 mb
30 mb
40 mb
50 mb
60 mb
70 mb
80 mb
90 mb
100 mb
110 mb
120 mb
130 mb
17
11
Chr11p15 (11:1-21,700,000)
vs
Chr17q23 (17:57,600,001-62,600,000)

m

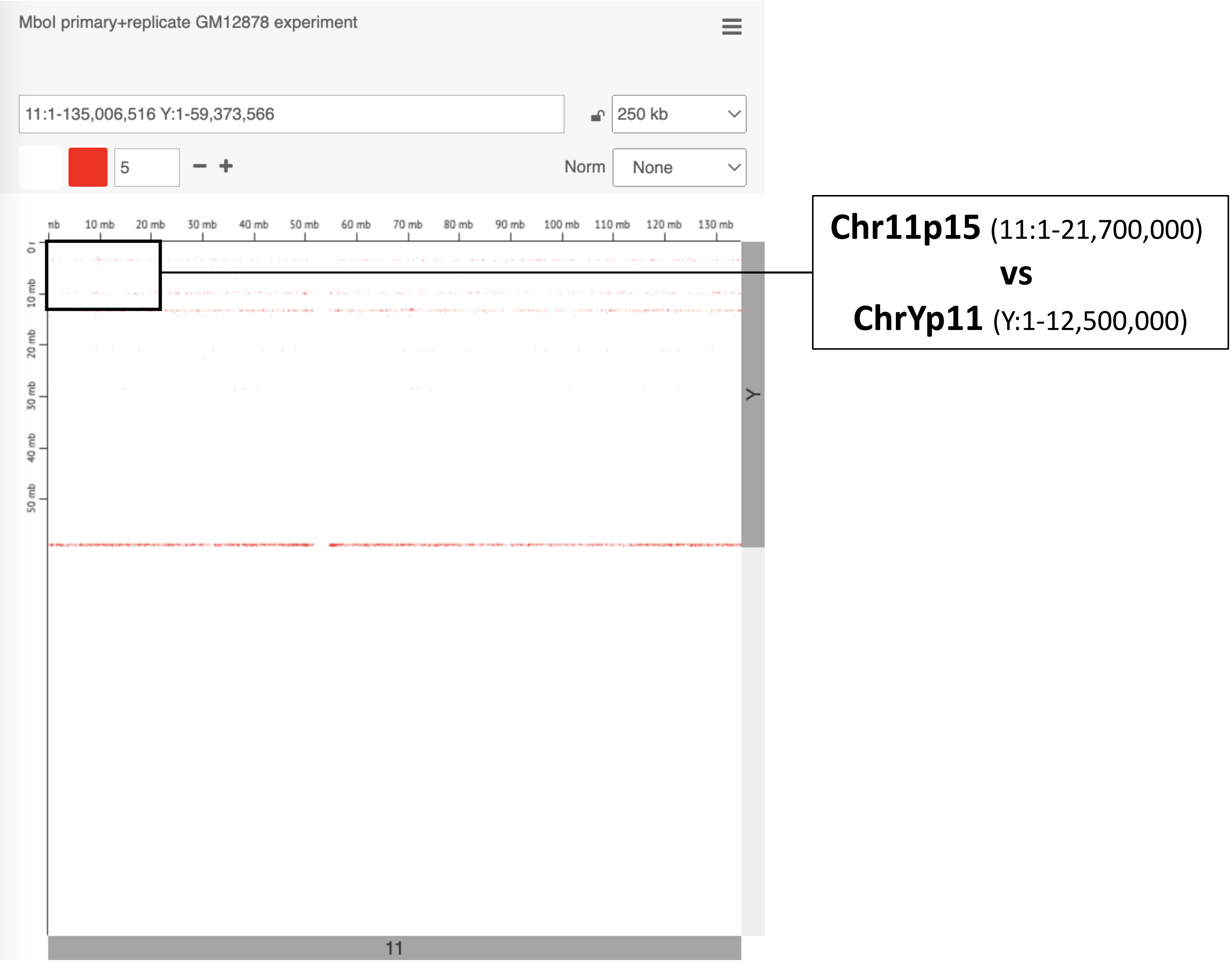

MboI primary+replicate GM12878 experiment
11:1-135,006,516 Y:1-59,373,566
250 kb
5
Norm
None
10 mb
20 mb
30 mb
40 mb
50 mb
60 mb
70 mb
80 mb
90 mb
100 mb
110 mb
120 mb
130 mb
Y
11
Chr11p15 (11:1-21,700,000)
vs
ChrYp11 (Y:1-12,500,000)

n

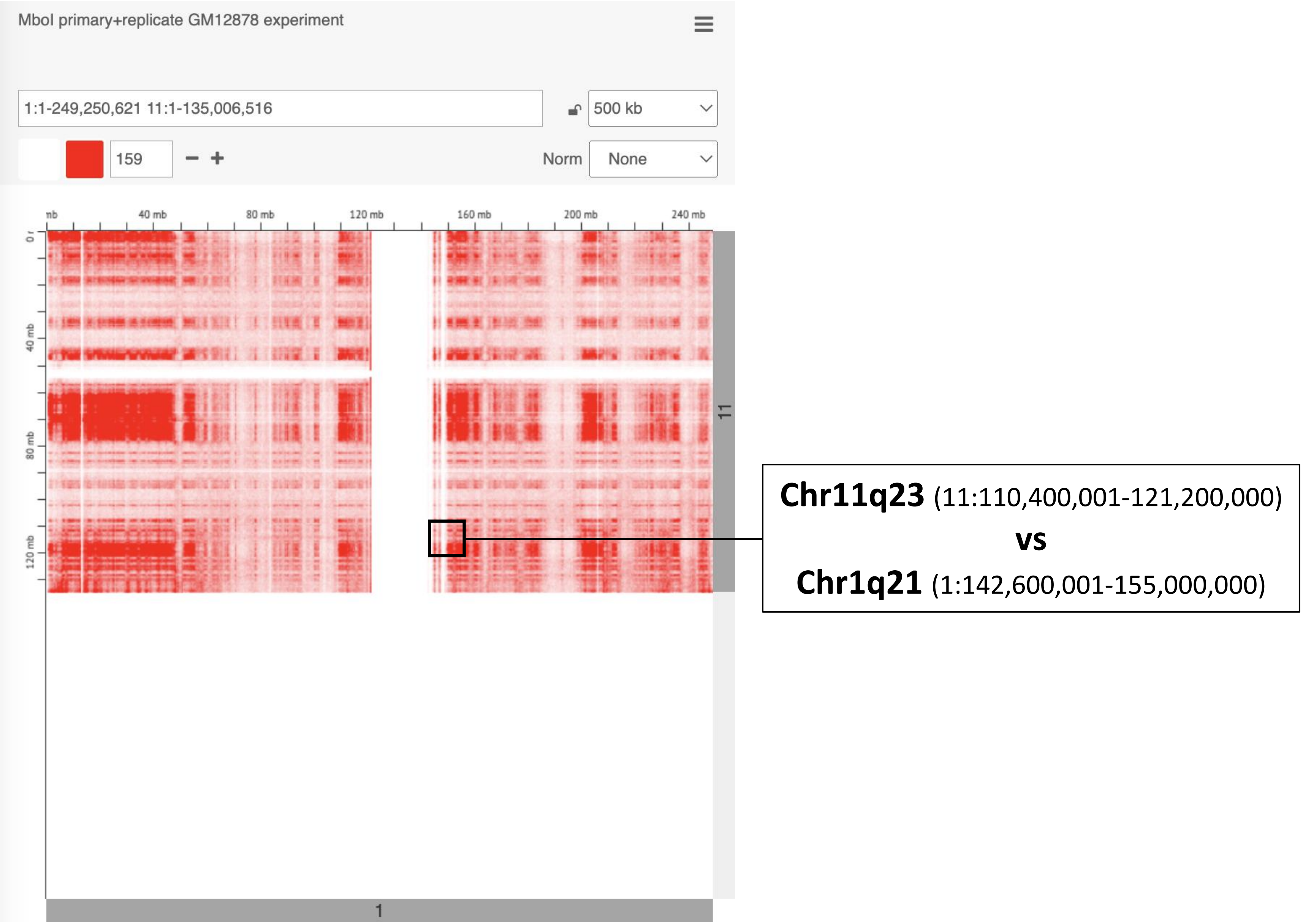

MboI primary+replicate GM12878 experiment
1:1-249,250,621 11:1-135,006,516
500 kb
159
Norm
None
40 mb
80 mb
120 mb
160 mb
200 mb
240 mb
11
1
Chr11q23 (11:110,400,001-121,200,000)
vs
Chr1q21 (1:142,600,001-155,000,000)

o

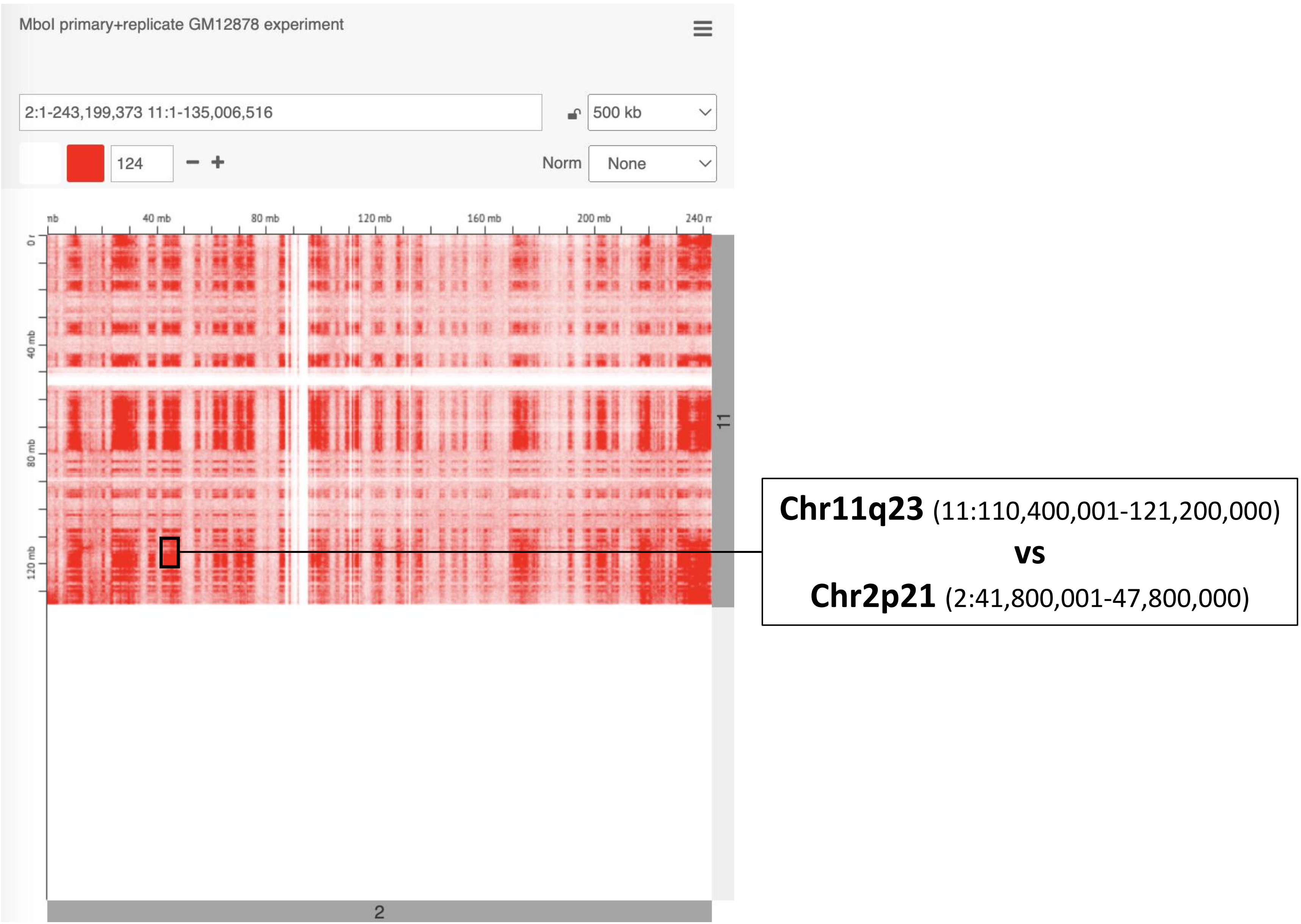

MboI primary+replicate GM12878 experiment
2:1-243,199,373 11:1-135,006,516
500 kb
124
Norm
None
40 mb
80 mb
120 mb
160 mb
200 mb
11
2
Chr11q23 (11:110,400,001-121,200,000)
vs
Chr2p21 (2:41,800,001-47,800,000)

p

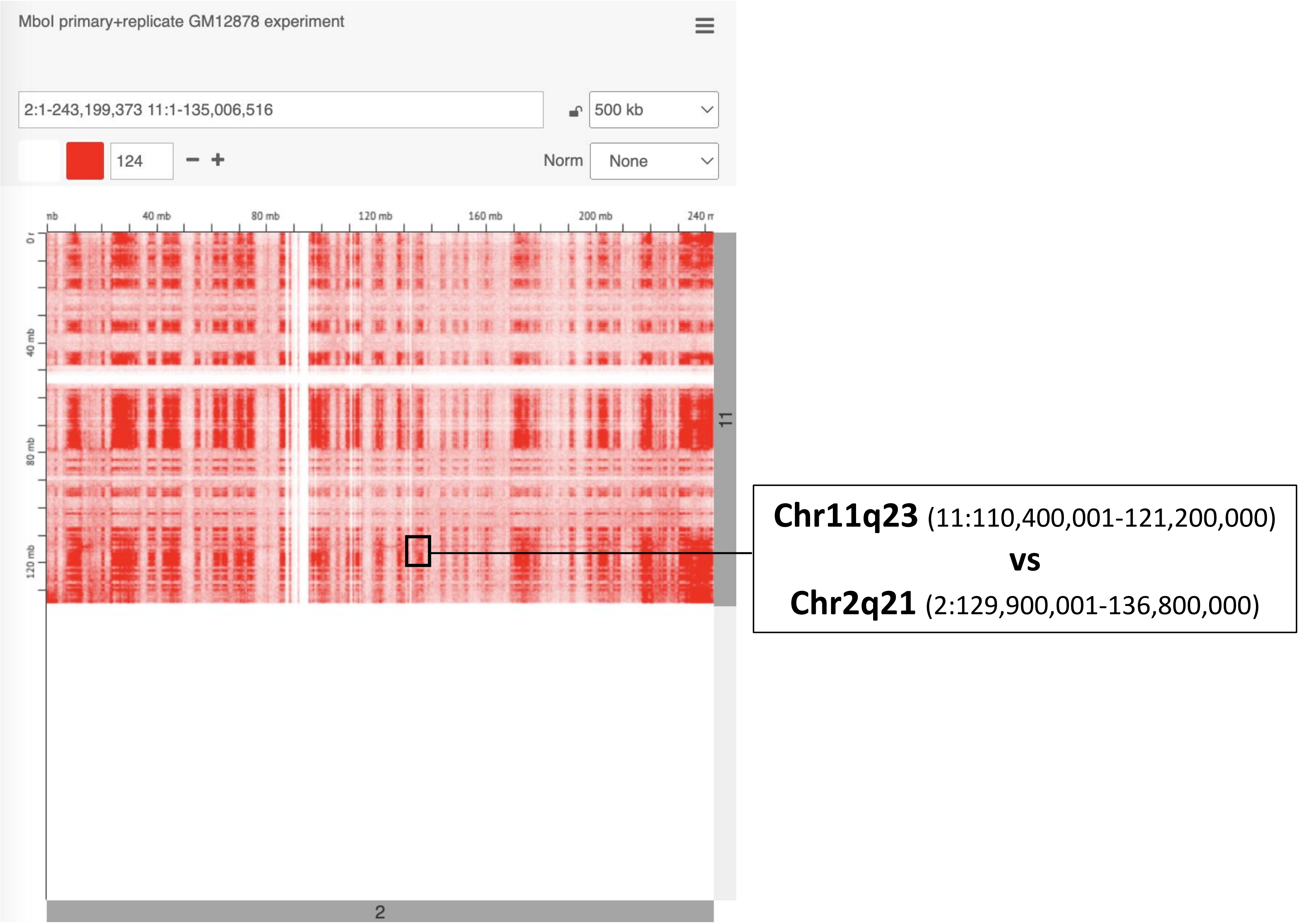
MboI primary+replicate GM12878 experiment
2:1-243,199,373 11:1-135,006,516
500 kb
124
Norm
None
40 mb
80 mb
120 mb
160 mb
200 mb
40 mb
80 mb
120 mb
11
2
Chr11q23 (11:110,400,001-121,200,000)
vs
Chr2q21 (2:129,900,001-136,800,000)

q

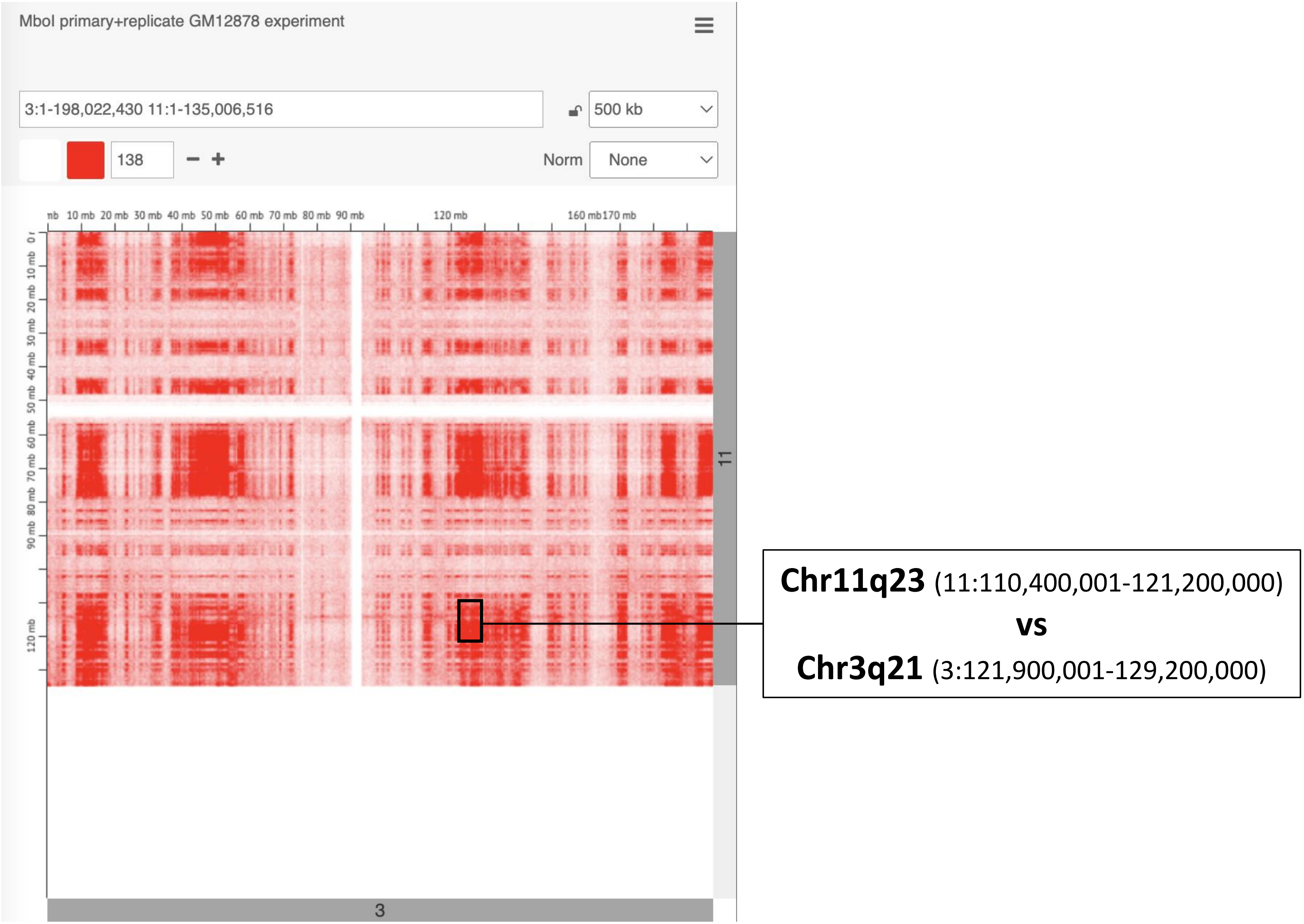
MboI primary+replicate GM12878 experiment
3:1-198,022,430 11:1-135,006,516
500 kb
138
Norm
None
10 mb 20 mb 30 mb 40 mb 50 mb 60 mb 70 mb 80 mb 90 mb
120 mb
160 mb170 mb
11
3
Chr11q23 (11:110,400,001-121,200,000)
vs
Chr3q21 (3:121,900,001-129,200,000)

r

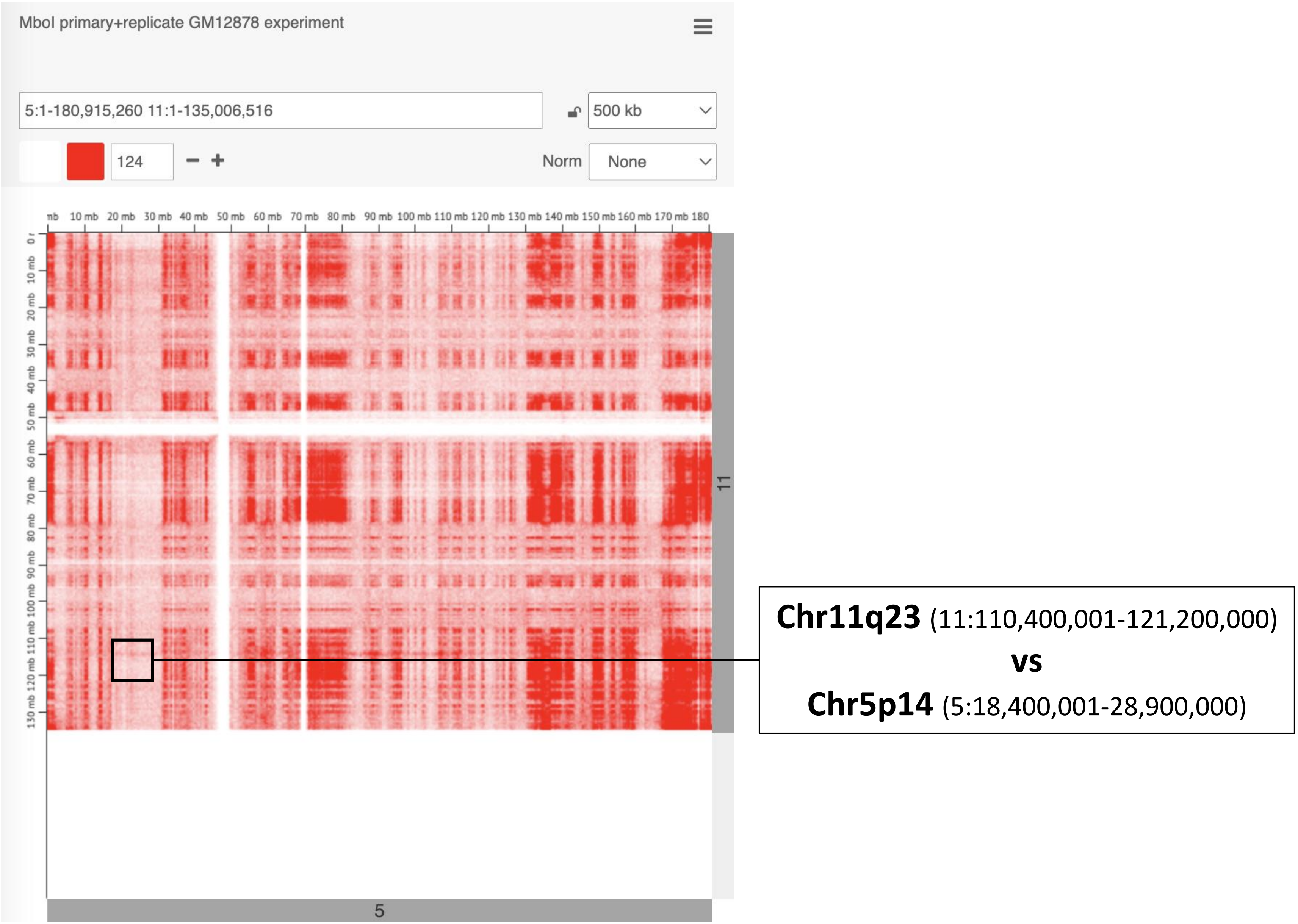

MboI primary+replicate GM12878 experiment
5:1-180,915,260 11:1-135,006,516
500 kb
124
Norm
None
5
11
Chr11q23 (11:110,400,001-121,200,000)
vs
Chr5p14 (5:18,400,001-28,900,000)

S

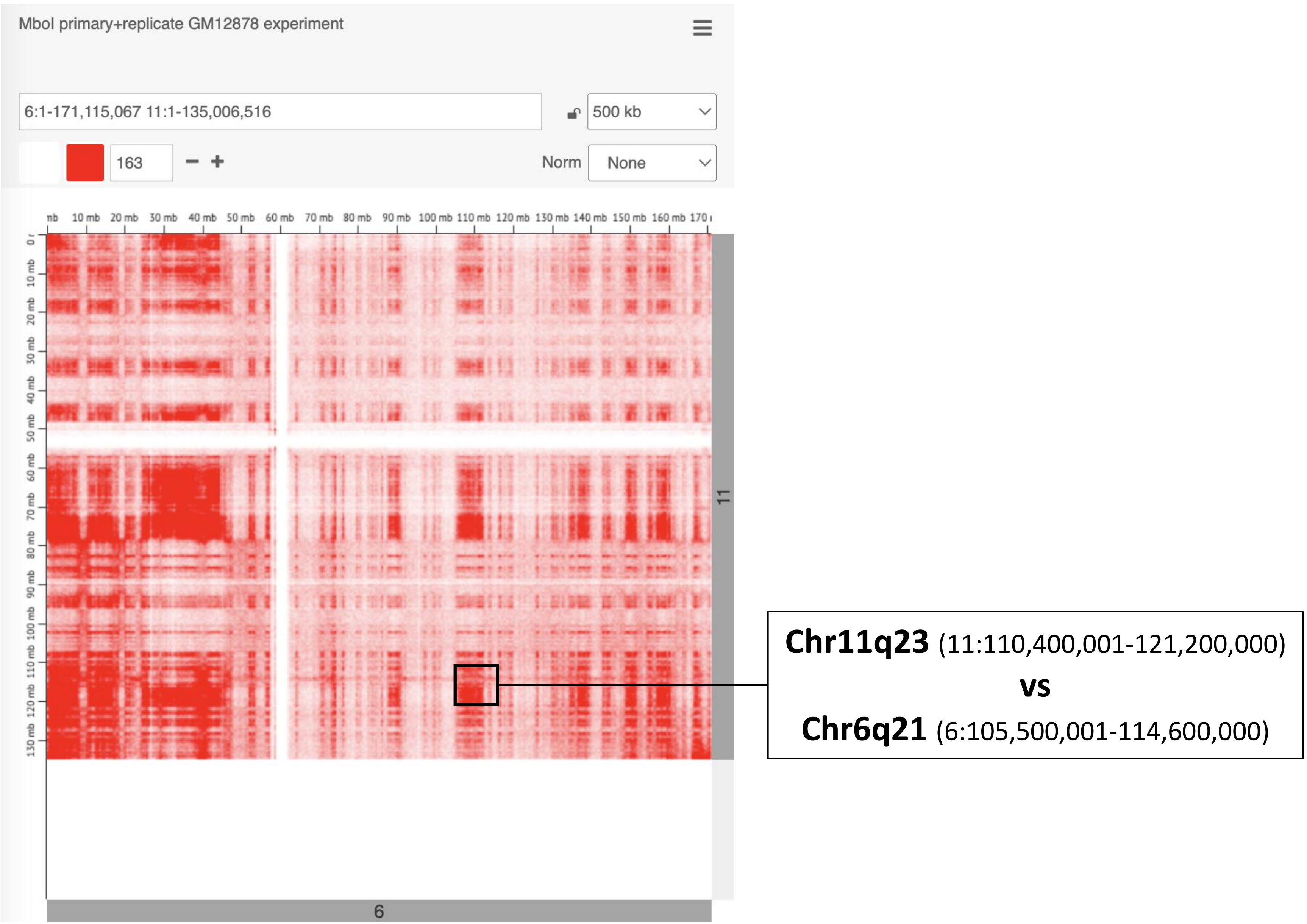

MboI primary+replicate GM12878 experiment
6:1-171,115,067 11:1-135,006,516
500 kb
163
Norm
None
11
6
Chr11q23 (11:110,400,001-121,200,000)
vs
Chr6q21 (6:105,500,001-114,600,000)

t

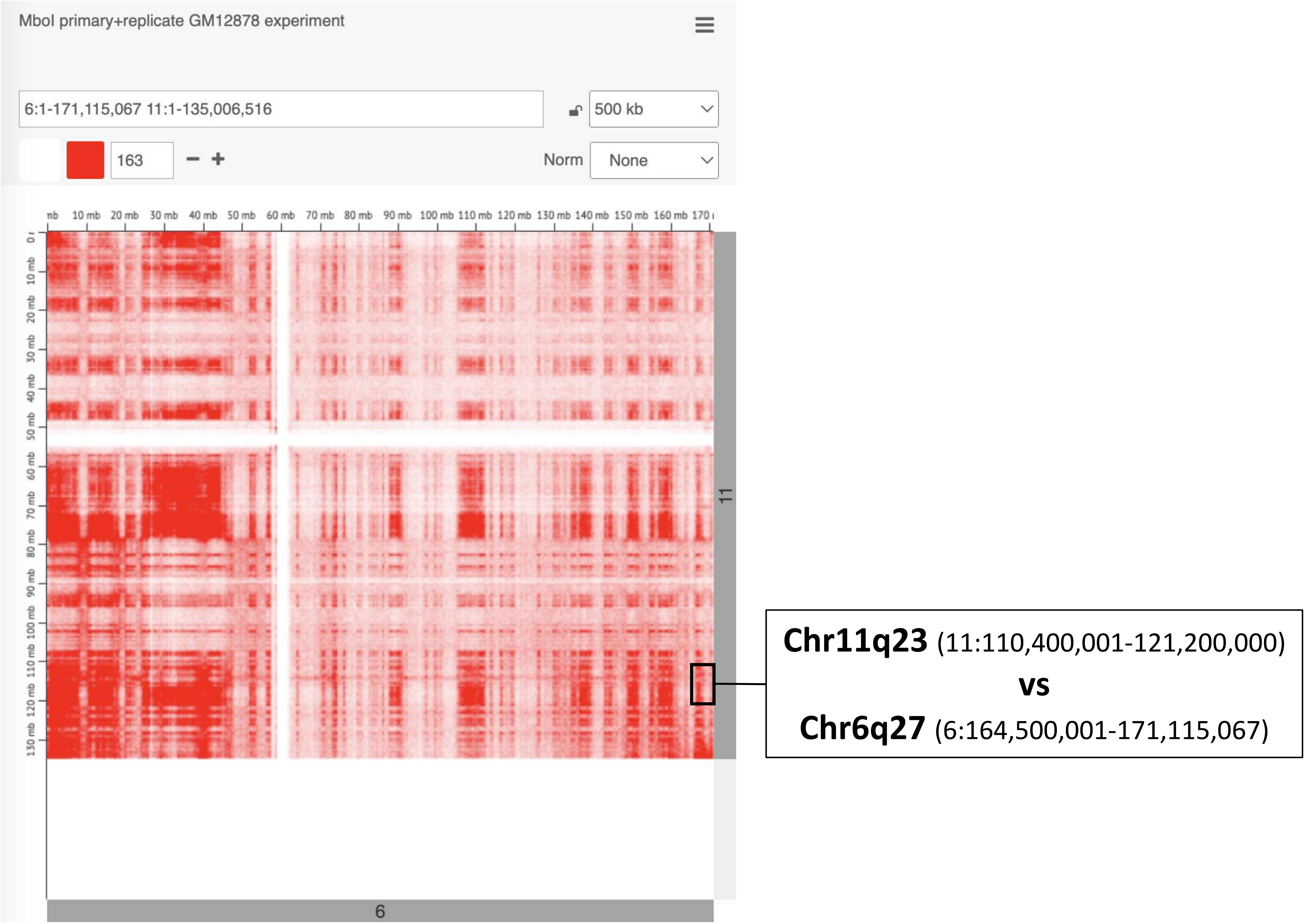

Mbol primary+replicate GM12878 experiment
6:1-171,115,067 11:1-135,006,516
500 kb
163
Norm
None
10 mb 20 mb 30 mb 40 mb 50 mb 60 mb 70 mb 80 mb 90 mb 100 mb 110 mb 120 mb 130 mb 140 mb 150 mb 160 mb 170
10 mb 20 mb 30 mb 40 mb 50 mb 60 mb 70 mb 80 mb 90 mb 100 mb 110 mb 120 mb 130 mb
11
6
Chr11q23 (11:110,400,001-121,200,000)
vs
Chr6q27 (6:164,500,001-171,115,067)

u

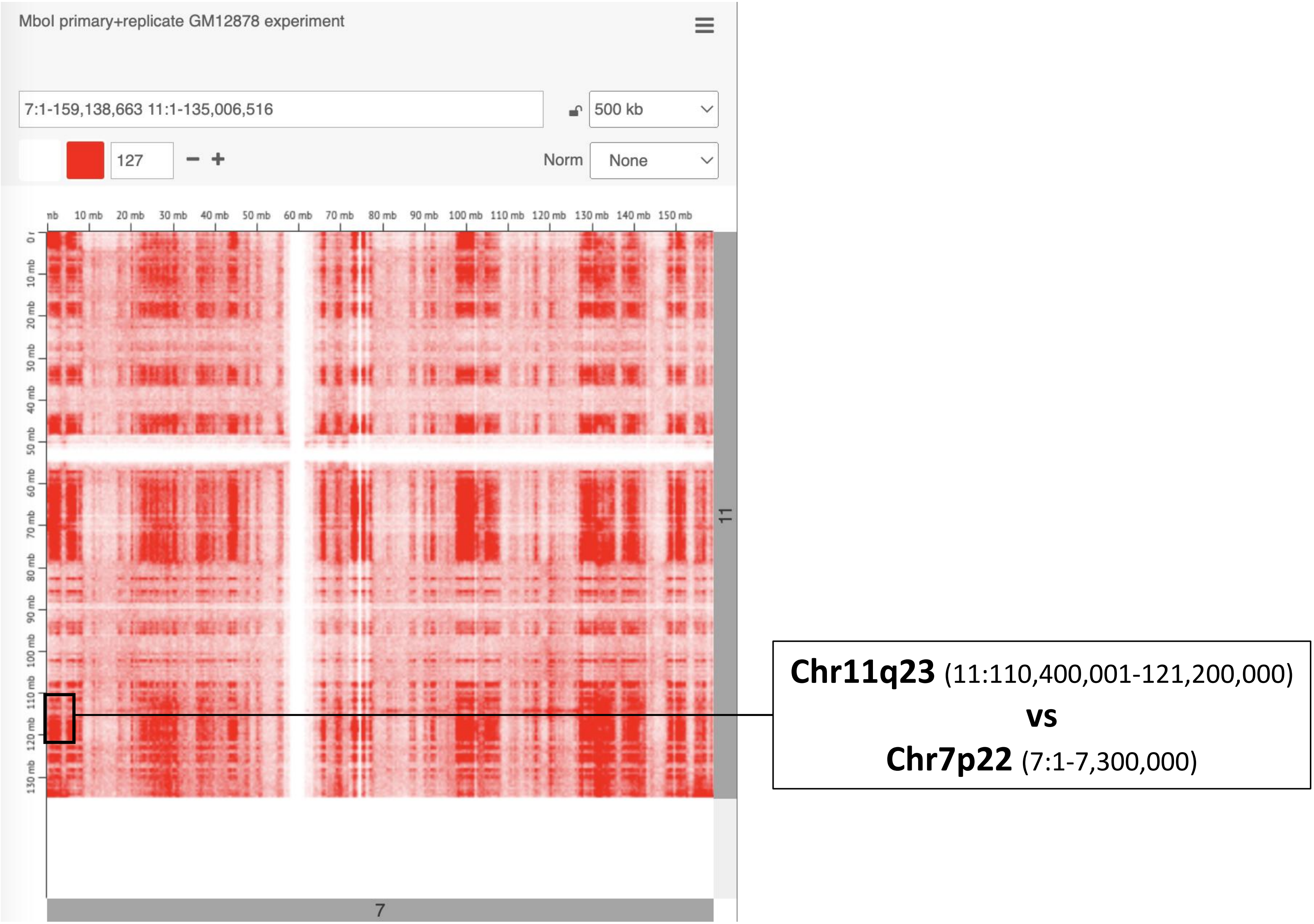

MboI primary+replicate GM12878 experiment
7:1-159,138,663 11:1-135,006,516
500 kb
127
Norm
None
10 mb
20 mb
30 mb
40 mb
50 mb
60 mb
70 mb
80 mb
90 mb
100 mb
110 mb
120 mb
130 mb
140 mb
150 mb
11
7
Chr11q23 (11:110,400,001-121,200,000)
vs
Chr7p22 (7:1-7,300,000)

V

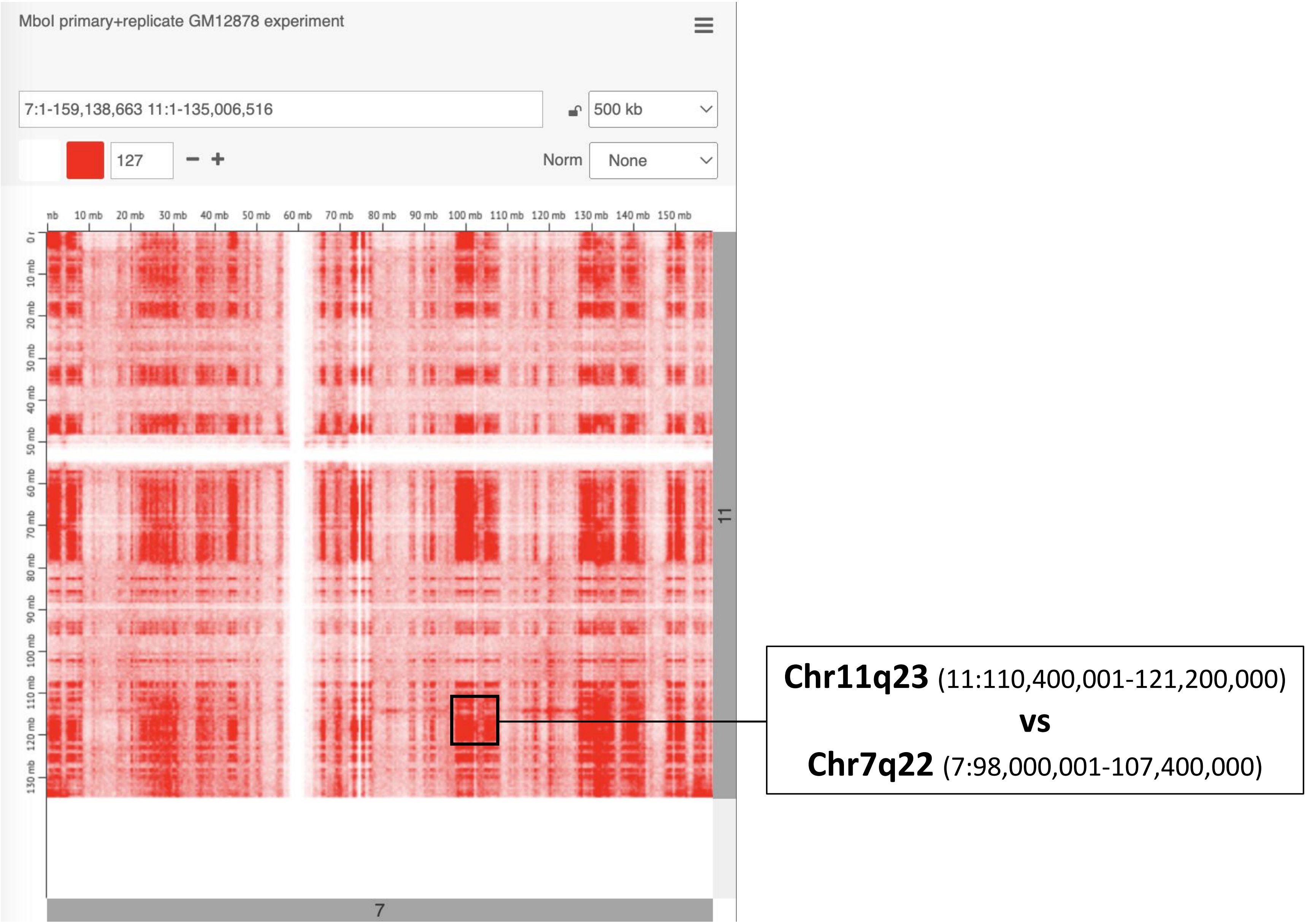

MboI primary+replicate GM12878 experiment
7:1-159,138,663 11:1-135,006,516
500 kb
127
Norm
None
7
11
Chr11q23 (11:110,400,001-121,200,000)
vs
Chr7q22 (7:98,000,001-107,400,000)

w

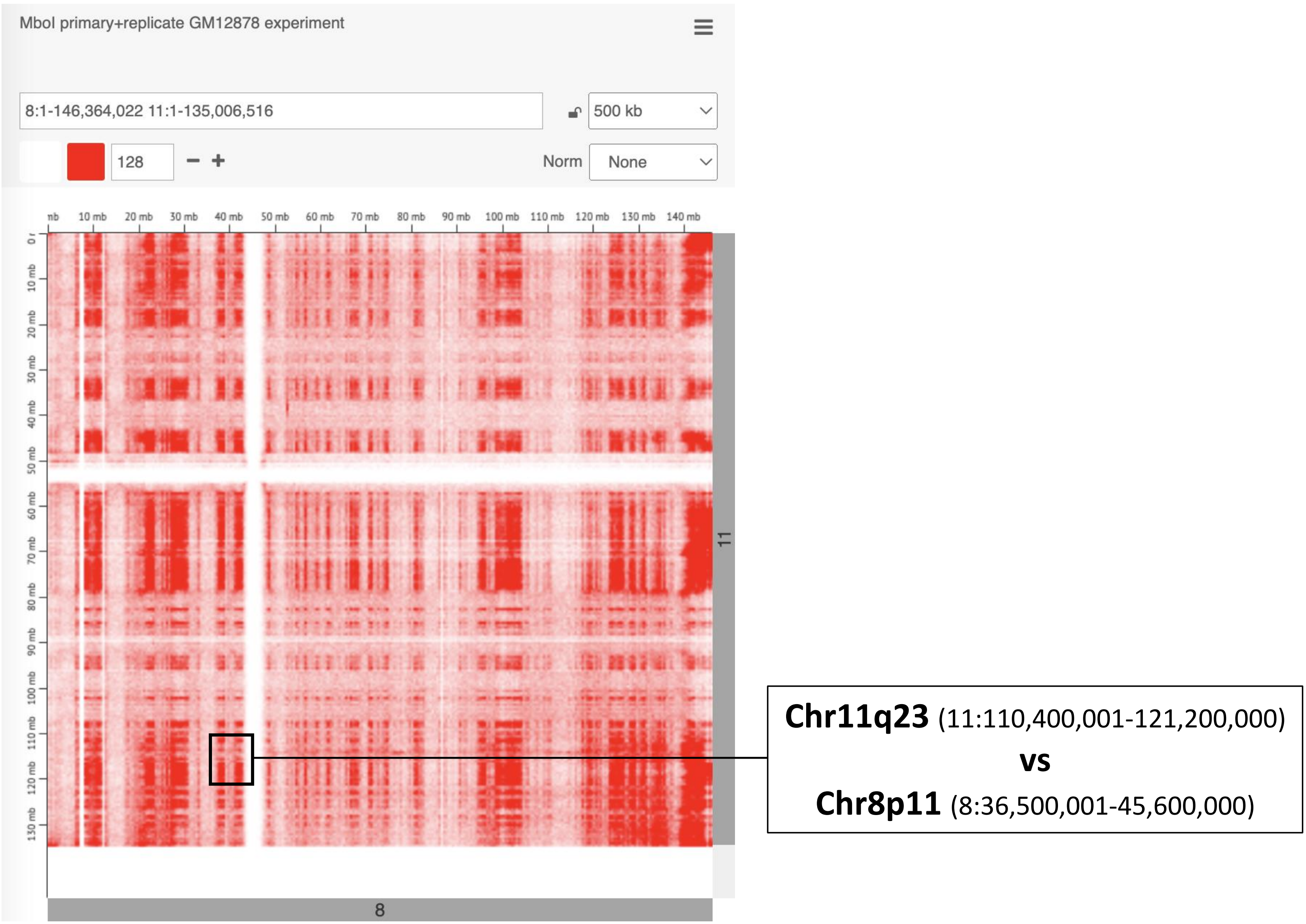
MboI primary+replicate GM12878 experiment
8:1-146,364,022 11:1-135,006,516
500 kb
128
Norm
None
11
8
Chr11q23 (11:110,400,001-121,200,000)
vs
Chr8p11 (8:36,500,001-45,600,000)

X

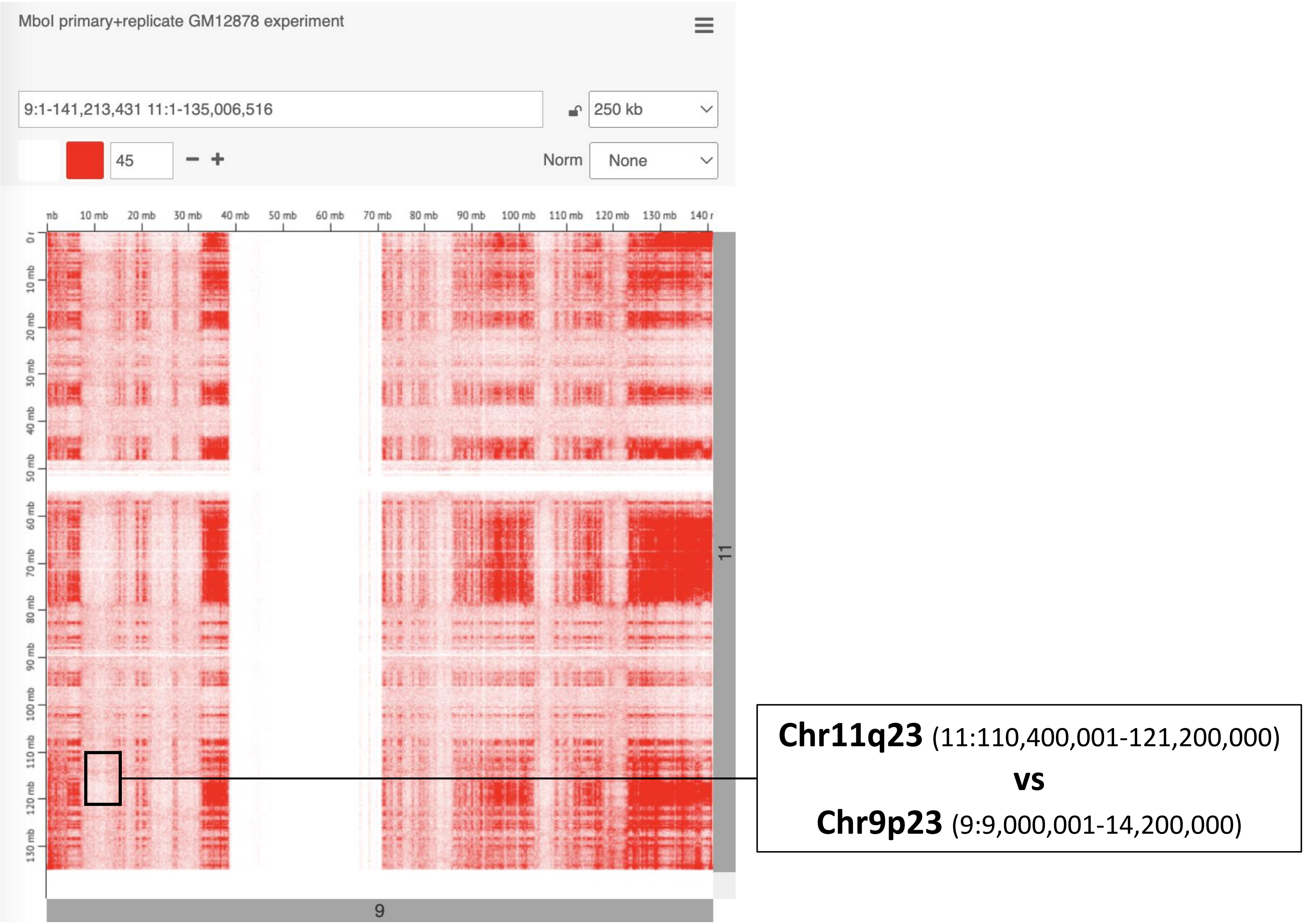

MboI primary+replicate GM12878 experiment
9:1-141,213,431 11:1-135,006,516
250 kb
45
Norm
None
10 mb
20 mb
30 mb
40 mb
50 mb
60 mb
70 mb
80 mb
90 mb
100 mb
110 mb
120 mb
130 mb
11
9
Chr11q23 (11:110,400,001-121,200,000)
vs
Chr9p23 (9:9,000,001-14,200,000)

y

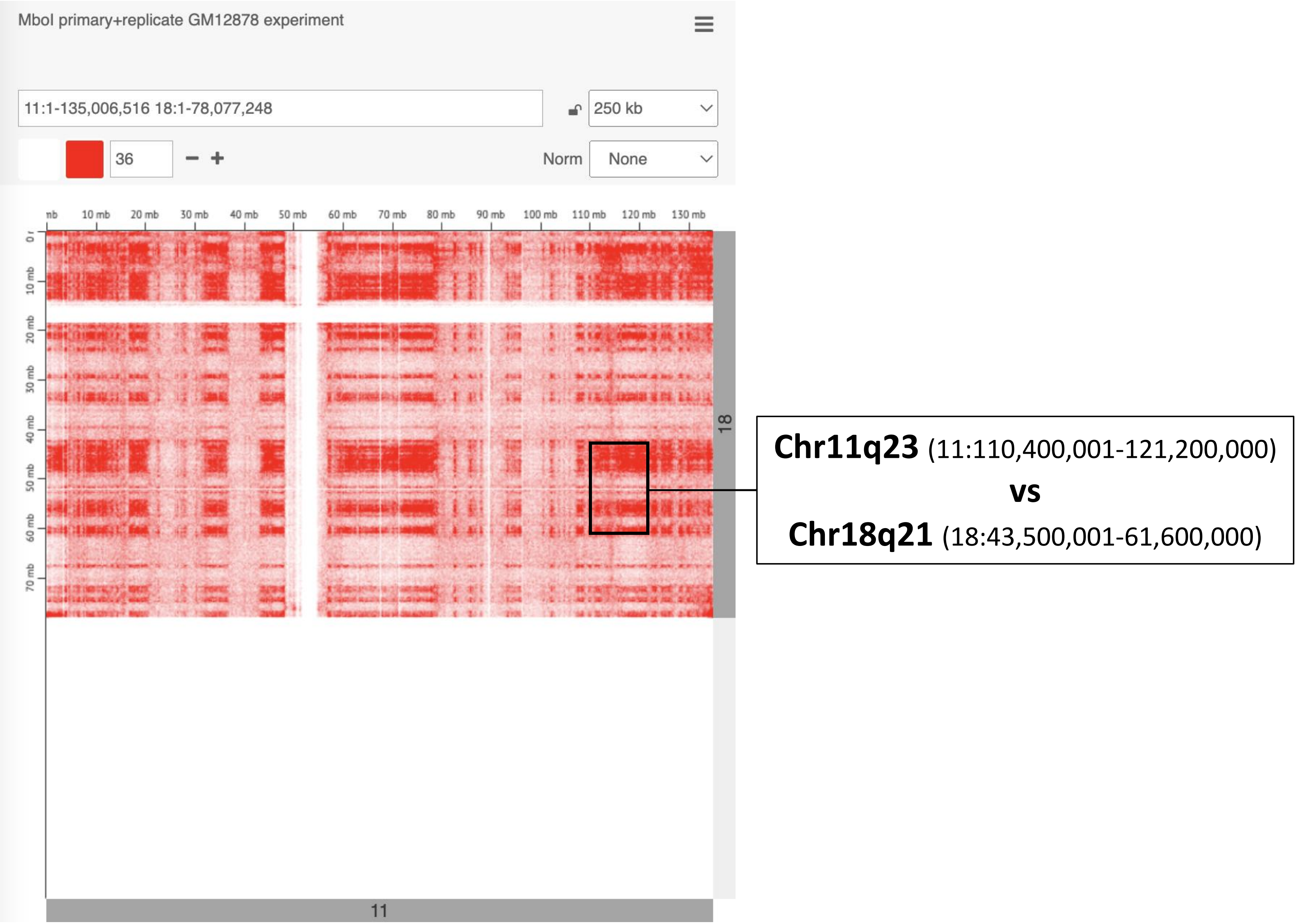

MboI primary+replicate GM12878 experiment
11:1-135,006,516 18:1-78,077,248
250 kb
36
Norm
None
10 mb
20 mb
30 mb
40 mb
50 mb
60 mb
70 mb
80 mb
90 mb
100 mb
110 mb
120 mb
130 mb
18
11
Chr11q23 (11:110,400,001-121,200,000)
vs
Chr18q21 (18:43,500,001-61,600,000)

Z

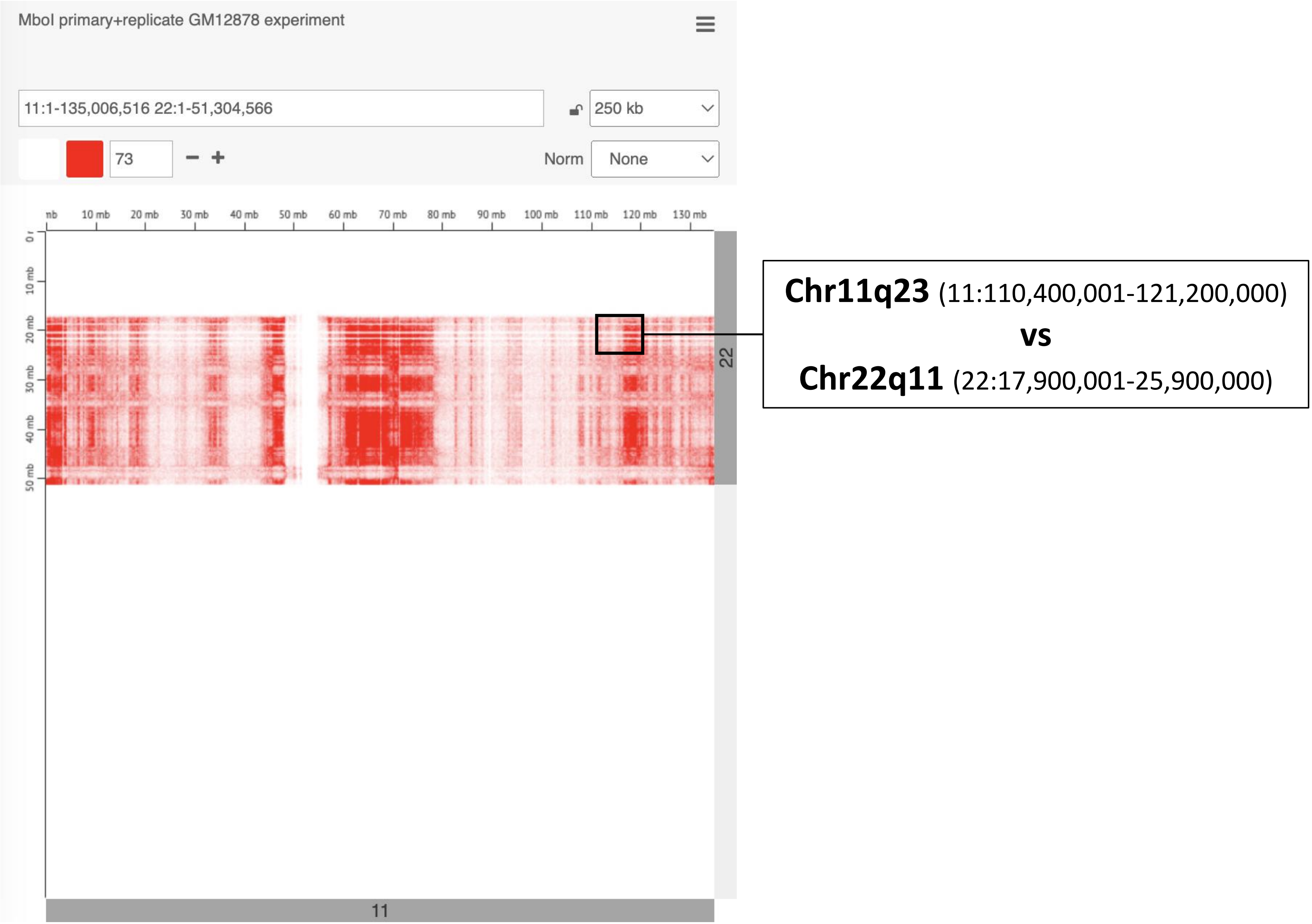

MboI primary+replicate GM12878 experiment
11:1-135,006,516 22:1-51,304,566
250 kb
73
Norm
None
10 mb
20 mb
30 mb
40 mb
50 mb
60 mb
70 mb
80 mb
90 mb
100 mb
110 mb
120 mb
130 mb
22
11
Chr11q23 (11:110,400,001-121,200,000)
vs
Chr22q11 (22:17,900,001-25,900,000)

Table S1: Sex distribution of patients showing chromosome 11 anomalies identified by conventional chromosome analysis.

Sex	Translocations	Inversions	Insertion	Deletions	Total
male	72	3	0	1	76
female	66	11	1	1	79
total	138	14	1	2	155

Table S2: Sex distribution of patients showing chromosome 11 anomalies identified by array-CGH.

Sex	Deletions	Duplications	Total
male	23	18	41
female	12	14	26
total	35	32	67

Table S3. Inheritance of translocations.

chromosome	paternal	maternal	de novo	unknown	total
1	2	1	0	4	7
2	2	0	1	6	9
3	0	1	4	1	6
4	0	2	0	5	7
5	0	3	1	2	6
6	0	1	0	4	5
7	1	2	0	6	9
8	2	4	0	4	10
9	1	1	1	2	5
10	0	2	0	4	6
12	1	1	1	0	3
13	0	2	0	1	3
14	0	0	0	3	3
15	1	0	0	1	2
16	0	0	0	1	1
17	1	1	0	1	3
18	0	2	1	0	3
21	2	0	0	0	2
22	4	16	2	23	45
X	0	0	1	1	2
Y	0	0	1	0	1
total	17	39	13	69	138

Table S4. Distribution of translocation breakpoints on chromosome 11 partners. Based on Glusman's mapping (see ref. 4 in the main text), OR gene families are present or absent[1].

Chromosome	Bkps in cytoband with ORs	Bkps in cytoband without ORs	Tot bkps (%)	Chromosome lenght (Mb)	Average number of bkps per 100 Mb
1	2	5	7 (5.1)	249	2.8
2	3	6	9 (6.5)	243	3.7
3	3	3	6 (4.3)	198	3.0
4	1	6	7 (5.1)	191	3.7
5	0	6	6 (4.3)	181	3.3
6	3	2	5 (3.6)	171	2.9
7	1	8	9 (6.5)	159	5.7
8	0	10	10 (7.2)	146	6.8
9	2	3	5 (3.6)	141	3.5
10	1	5	6 (4.3)	136	4.4
12	0	3	3 (2.2)	134	2.2
13	1	2	3 (2.2)	115	2.6
14	2	1	3 (2.2)	107	2.8
15	1	1	2 (1.4)	103	1.9
16	0	1	1 (0.7)	90	1.1
17	3	0	3 (2.2)	81	3.7
18	1	2	3 (2.2)	78	3.85
19	0	0	0	59	0.0
20	0	0	0	63	0.0
21	2	0	2 (0.7)	48	4.2
22	0	45	45 (32.6)	51	88.2
X	0	2	2 (1.4)	155	1.3
Y	0	1	1 (0.7)	59	1.7
total	26 (18.8%)	112 (81.2%)	138		

Bkps: breakpoints

Table S5. Cytobands involved in the main collected translocations, visualized on the Hi-C maps

Chromosome 11 cytoband (chromosome coordinates, hg19)	Partner chromosome cytoband (chromosome coordinates, hg19)	Figure S5 panel showing interchromosomal interactions
Chr11p15 (11:1-21,700,000)	Chr2q31 (2:169,700,001-183,000,000)	a
	Chr4p14 (4:35,800,001-41,200,000)	b
	Chr5p15 (5:1-18,400,000)	c
	Chr5q31 (5:130,600,001-144,500,000)	d
	Chr6p23 (6:13,400,001-15,200,000)	e
	Chr6q16 (6:93,100,001-105,000,000)	f
	Chr7p11 (7:54,000,001-59,900,000)	g
	Chr7q21 (7:77,500,001-98,000,000)	h
	Chr8q24 (8:117,700,001-146,364,022)	i
	Chr9p13 (9:33,200,001-41,000,000)	j
	Chr12q15 (12:67,700,001-71,500,000)	k
	Chr17q23 (17:57,600,001-62,600,000)	l
	ChrYp11 (Y:1-12,500,000)	m
Chr11q23 (11:110,400,001-121,200,000)	Chr1q21 (1:142,600,001-155,000,000)	n
	Chr2p21 (2:41,800,001-47,800,000)	o
	Chr2q21 (2:129,900,001-136,800,000)	p
	Chr3q21 (3:121,900,001-129,200,000)	q
	Chr5p14 (5:18,400,001-28,900,000)	r
	Chr6q21 (6:105,500,001-114,600,000)	s
	Chr6q27 (6:164,500,001-171,115,067)	t
	Chr7p22 (7:1-7,300,000)	u
	Chr7q22 (7:98,000,001-107,400,000)	v
	Chr8p11 (8:36,500,001-45,600,000)	w
	Chr9p23 (9:9,000,001-14,200,000	x
	Chr18q21 (18:43,500,001-61,600,000)	y
	Chr22q11 (22:17,900,001-25,900,000)	z

Supplemental Methods.

1) Array Comparative Genomic Hybridization (Array-CGH)

The *Cytogenetics, Molecular Genetics and Medical Genetics Unit, Toma Advanced Biomedical Assays* used several platform of analysis.

1) Samples until 2016. A genome-wide BAC platform (ConstitutionalChip 4.0, PerkinElmer Wallac, Turku, Finland) with a dye-swap approach. The average spatial resolution is 600 Kbp and the reference DNAs were commercial pools of human male or female DNAs (Promega Corporation, Madison, Wisconsin, USA). The referred samples were analysed using a dye-swap experiment by reversal dye approach hybridizing the sample DNA against a sex matched human commercial DNA reference (Promega). Data were analysed with the OneClickCGH 4.3.3 Software (PerkinElmer).
2) Samples from 2016 to 2020. Post-natal samples were run on a 135K-feature, whole-genome oligonucleotide-based microarray (Roche NimbleGen, Madison, Wis., USA). Data were analysed and displayed with Genoglyphix analysis software 2.6 (Signature Genomics, a subsidiary of PerkinElmer, Inc., Spokane, Wash., USA). Pre-natal samples were run on an oligo genome-wide CGX Array 37K (PerkinElmer). Resolution: in critical regions 1 probe/10Kb, in backbone 1 probe/100Kb. Design: whole-genome oligonucleotide-based microarray analysing the backbone and more than 980 regions including >200 critical regions for known microdeletion/microduplication syndromes, 41 subtelomeres, 43 pericentromeric regions, >200 transcription factors and >200 developmental genes. Genoglyphix 3.0 software (Signature Genomics, PerkinElmer) is set to use a segmentation algorithm flagging segments of copy-number gain or loss involving a minimum of 5 consecutive probes and a log2 of the normalized ratio of the sample: control signal intensities (log2 ratio) of ±0.3 which provides an average effective resolution of 40Kb in the critical region and 400Kb in the backbone.
3) Samples from 2020 onwards. Post-natal samples were run on an oligo genome-wide GenetiSure Cyto 4x180K CGH+SNP (Agilent). Labelling of genomic DNA and hybridization is conducted by aCGH kit (Agilent). Microarray analysis is performed by Cytogenomics software v5.0.2.5 (Agilent). The platform analyses 3644 genes, with the following resolution: 1 probe/7.3 kb in critical regions, 1 probe/57.1 kb in backbone, 1/25 kb in subtelomeric region and 1/10.5 Kb in PAR region. The software is set to use ADM2 algorithm flagging segments of copy-number gain or loss involving a minimum of 5 consecutive probes with an average effective resolution of 29.2 kb in the critical regions and 228.4 kb in the backbone and 8Mb for LOH. Pre-natal samples were run on an oligo genome-wide GenetiSure Cyto 8x60K CGH (Agilent). Labelling of genomic DNA and hybridization is conducted by aCGH kit (Agilent). Microarray analysis is performed by Cytogenomics software v5.1.2.1 (Agilent). The platform analyses 3644 genes, with the following resolution: 1 probe every 7.1 kb in critical regions, 1 every 67.4 kb in backbone, 1 every 31.8 kb in subtelomeric region and 1 every 13.5 Kb in PAR regions. The software is set to use ADM2 algorithm flagging segments of copy-number gain or loss involving a minimum of 5 consecutive probes with an average effective resolution of 28.4 kb in the critical regions and 269.6 kb in the backbone.

2) **Juicebox Aiden Lab Tool** (http://www.aidenlab.org/juicebox/), a Hi-C data visualization software, was used to investigate the possible proximity of the chromosomal territories between chromosome 11 and its partner chromosomes involved in the collected translocations. Experiment ENCSR410MDC (ENCFF718AWL), with reference assembly hg19, carried out on Homo sapiens GM12878 cell line and whose fastq raw data files had been deposited in ENCODE by Erez, Aiden and Baylor Lab, was selected as reference dataset. The interaction regions between each of the two main cytobands of chromosome 11 involved in the translocations (11p15 and 11q23) and those of their respective partner chromosomes were visualized on the Hi-C maps (Table S5 and Figure S4). Genomic coordinates of the cytobands were obtained from UCSC Genome Browser on Human (GRCh37/hg19) (https://genome.ucsc.edu/). We observed territorial proximity for several of the cytobands involved in the translocations, as indicated by the deep red staining of their interactions on the Hi-C heat maps (Figure S5 a, b, d, e, i, j, n, o, p, q, s, t, u, v, w, y).
