# Supplementary material for: Olfactory receptor genes and chromosome 11 structural aberrations: Players or spectators?
Source: HGG Adv. 2023 Dec 30;5(2):100261. doi: 10.1016/j.xhgg.2023.100261 (PMC10820794; doi:10.1016/j.xhgg.2023.100261)
Supplement: Document S2. Article plus supplemental information [file mmc2.pdf]

HGG Advances

ARTICLE

# Olfactory receptor genes and chromosome 11 structural aberrations: Players or spectators?

Serena Redaelli,[1] Francesca Romana Grati,[2] Viviana Tritto,[3] Giuliana Giannuzzi,[4] Maria Paola Recalcati,[5] Elena Sala,[6] Nicoletta Villa,[6] Francesca Crosti,[6] Gaia Roversi,[1,6] Francesca Malvestiti,[2] Valentina Zanatta,[2] Elena Repetti,[2] Ornella Rodeschini,[5] Chiara Valtorta,[5] Ilaria Catusi,[5] Lorenza Romitti,[7] Emanuela Martinoli,[3] Donatella Conconi,[1] Leda Dalprà,[1,6] Marialuisa Lavitrano,[1] Paola Riva,[3,8] and Angela Bentivegna[1,8,9,*]

## Summary

The largest multi-gene family in metazoans is the family of olfactory receptor (OR) genes. Human ORs are organized in clusters over most chromosomes and seem to include >0.1% the human genome. Because 369 out of 856 OR genes are mapped on chromosome 11 (HSA11), we sought to determine whether they mediate structural rearrangements involving this chromosome. To this aim, we analyzed 220 specimens collected during diagnostic procedures involving structural rearrangements of chromosome 11. A total of 222 chromosomal abnormalities were included, consisting of inversions, deletions, translocations, duplications, and one insertion, detected by conventional chromosome analysis and/or fluorescence *in situ* hybridization (FISH) and array comparative genomic hybridization (array-CGH). We verified by bioinformatics and statistical approaches the occurrence of breakpoints in cytobands with or without OR genes. We found that OR genes are not involved in chromosome 11 reciprocal translocations, suggesting that different DNA motifs and mechanisms based on homology or non-homology recombination can cause chromosome 11 structural alterations. We also considered the proximity between the chromosomal territories of chromosome 11 and its partner chromosomes involved in the translocations by using the deposited Hi-C data concerning the possible occurrence of chromosome interactions. Interestingly, most of the breakpoints are located in regions highly involved in chromosome interactions. Further studies should be carried out to confirm the potential role of chromosome territories' proximity in promoting genome structural variation, so fundamental in our understanding of the molecular basis of medical genetics and evolutionary genetics.

## Introduction

Structural variants represent a type of genome mutation that can be balanced or unbalanced on the basis of a possible loss or gain of a functional genomic portion. If there is an imbalance, the aberration may be embryonically lethal, or, in the best-case scenario, it may have negative effects on a child's development or on an adult's ability to reproduce.[1] Structural variants are known to derive from three major mutational mechanisms: non-homologous end joining (NHEJ), non-allelic homologous recombination (NAHR), and replication-based microhomology-mediated break-induced replication (MMBIR). All three homologous (NAHR) and non-homologous/microhomologous (NHEJ and MMBIR) events are crucial for genomic DNA rearrangements and genome evolution.[1] Genome architectural motifs, such as repeated sequences, frequently encourage structural variations both in DNA-recombination-based events and in replication processes.[2] For several years, attention has been focused on possible chromosomal regions that contain highly homologous and repeated sequences and fragile or unstable loci, predisposing them to susceptibility to chromosomal rearrangements. Among the repeated sequences, those including the family of olfactory receptor (OR) genes stand out for their abundance, being one of the largest multi-gene families in metazoans. Human ORs were frequently found to be spread in clusters over most chromosomes, suggesting that the "olfactory subgenome" (the OR genes and their genomic environment) may include >0.1% the human genome.[3,4] All human chromosomes, with the exception of HSA20 and HSAY,[5,6] include OR genes, but HSA11 is by far the richest in OR genes, as they represent >10% the whole genes in chromosome 11. The HSA11 OR regions are enriched in LINE-1 retrotransposons, repetitive elements that contribute significantly to structural variation and may play a specific role in olfactory neurons' nuclear architecture.[7] In addition, Ou and colleagues demonstrated that the interchromosomal low-copy repeat (LCR) harboring the OR gene cluster in 11p15.4 is a novel

[1]School of Medicine and Surgery, University of Milano-Bicocca, 20900 Monza, Italy; [2]R&D, Cytogenetics, Molecular Genetics and Medical Genetics Unit, Toma Advanced Biomedical Assays S.p.A. (ImpactLab), 21052 Busto Arsizio, Italy; [3]Department of Medical Biotechnology and Translational Medicine, University of Milan, 20122 Milan, Italy; [4]Department of Biosciences, University of Milan, 20122 Milan, Italy; [5]IRCCS Istituto Auxologico Italiano, Medical Cytogenetics Laboratory, 20095 Cusano Milanino, Italy; [6]UC Medical Genetics, Fondazione IRCCS San Gerardo dei Tintori, 20900 Monza, Italy; [7]Pathology and Cytogenetics Laboratory, Clinical Pathology Department, Fondazione IRCCS Ca' Granda Ospedale Maggiore Policlinico, 20162 Milan, Italy
[8]These authors contributed equally
[9]Lead contact
*Correspondence: angela.bentivegna@unimib.it
https://doi.org/10.1016/j.xhgg.2023.100261.
© 2024 The Authors. This is an open access article under the CC BY license (http://creativecommons.org/licenses/by/4.0/).

genomic instability region that mediates the relatively common recurrent constitutional non-Robertsonian translocation t(4;11) by NAHR.[8] Moreover, in their computationally determined genome-wide "recurrent translocation map," some of the potential interchromosomal NAHR pairs represent OR gene repeats. Another well-known example of OR-mediated recurrent translocation is the t(4;8)(p16;p23), where heterozygous sub-microscopic inversion polymorphisms of the OR region at 8p23 play a crucial role in the generation of chromosomal imbalances through unusual meiotic exchanges.[9]

Based on this knowledge, we wondered if OR genes could trigger human chromosomal rearrangements. In particular, since 369 (43%) out of 856 OR genes are found on HSA11,[10] in this study, we examined structural rearrangements involving this chromosome in a series of cases collected during diagnostic procedures in five laboratories. A total of 222 chromosomal abnormalities involving chromosome 11 were gathered, including translocations, inversions, deletions, duplications, and one insertion. We searched for a possible association with the presence of OR genes as mediators and/or sites of breakage causing structural rearrangements by using conventional and molecular cytogenetics and a bioinformatics-statistical approach, suggesting that OR genes are not preferentially involved.

## Subjects and methods

### General sample data

The 220 samples were collected from five medical genetics laboratories with oversight by the respective institutional review boards and after written informed consent was obtained from parents or legal guardians. A total of 222 chromosomal abnormalities were identified, because two cases carry a double rearrangement: 155 were determined by conventional chromosome analysis of the karyotype and include 138 translocations, 14 inversions, 2 deletions, and 1 insertion (Table S1); the 65 remaining cases were found using array comparative genomic hybridization (array-CGH) and include 32 duplications and 35 deletions (Table S2). All investigations were carried out following precise clinical indications, such as suspicion of fetal abnormalities reported by ultrasound analysis in prenatal cases or complex pediatric pictures or reproductive problems in adults. In any case, no statistically significant differences between the two sexes were observed (chi-squared $p > 0.05$; Table S3). Some examples of identified alterations are shown in Figure S1.

### Chromosome analysis

Standard methods were applied to conduct chromosome analysis by QFQ and GTG banding as previously reported.[11] Detailed methods for fluorescence *in situ* hybridization (FISH) and array-CGH analysis are available in the supplemental methods.

### Statistics

We obtained the location of OR genes and coordinates of chromosome 11 cytogenetic bands in the hg19 reference from the UCSC genome browser (UCSC genes and chromosome band tracks, last access date: June 16, 2022). We considered as "OR cytoband" those cytobands where at least one OR gene was mapped and as "no OR cytoband" those where no OR gene was mapped. We excluded 11q23 from the "no OR cytoband" and calculated the total size (in base pairs) of cytobands in the two groups. We counted the number of translocations with chromosome 11 breakpoint in either cytoband group, excluding cases with breakpoint at 11q23, as t(11;22)(q23;q11) rearrangements are common and known to be mediated by AT-rich palindromes,[12] and cases with breakpoint at the centromere. We also excluded translocation cases for which a lack of information about the sub-band hampered the assignment to either group. The null distributions of copy-number variation (CNV) breakpoints were generated by performing 1,000 permutations along chromosome 11, excluding gaps, by using BEDTools v.2.30.0.[13] Statistical analyses were performed in R v.4.0.3.[14]

## Results

### Structural aberrations evidenced by conventional cytogenetic analysis

#### *Interchromosomal rearrangements: Translocations*

Our survey evidenced a general predisposition of chromosome 11 to rearrange with virtually all chromosomes without correlation between chromosome size and number of breakpoints (Figure 1). Notably, for some translocation partners, we identified breakpoints both in regions containing OR genes and in non-OR regions (Figure 2). Only chromosomes 19 and 20 do not show rearrangements, the latter being curiously one of the only two chromosomes without OR genes. The disproportion of the involvement of chromosome 22 compared to the other chromosomes emerged, with 45 cases of the well-known 11q;22q translocation, caused by the presence of palindromic AT-rich repeats (PATRRs) on 11q23 (PATRR11) and on 22q11 (PATRR22).[15] Ten (22.2%) out of 45 cases with 11q;22q translocation were identified in prenatal diagnosis as unbalanced with an extra der(22), inherited from a balanced mother. Only one case, a miscarriage, presented an imbalance with a supernumerary der(11) of paternal origin and the lack of a chromosome 22 (46,XX,+der(11)t(11;22)(q23;q11)pat,−22). In another miscarriage, the translocation 11;22 was balanced, but trisomy 22 was present (47,XX,t(11;22)(q23;q11.2),+22); the parents refused further investigation.

In order to identify a possible association with the presence of OR genes as mediators and/or sites of breakage, we first classified breakpoints from a cytomorphological and a cytogenetic point of view, according to the presence or absence of OR cluster genes in the cytobands, on the basis of Glusman's mapping[4] (Table S4; Figure S2). Only 18.8% breakpoints fall within a cytoband with OR genes. Regarding the partner of chromosome 11 translocations, we observed no correlation between chromosome size and the number of breakpoints. Considering the breakpoints on chromosome 11, excluding 45 cases of recurrent translocation 11q;22q, about 40% fall in regions that contain OR cluster genes (Table 1; Figure 4).

Then, we moved toward a more accurate statistical analysis, checking the location of OR genes by the coordinates

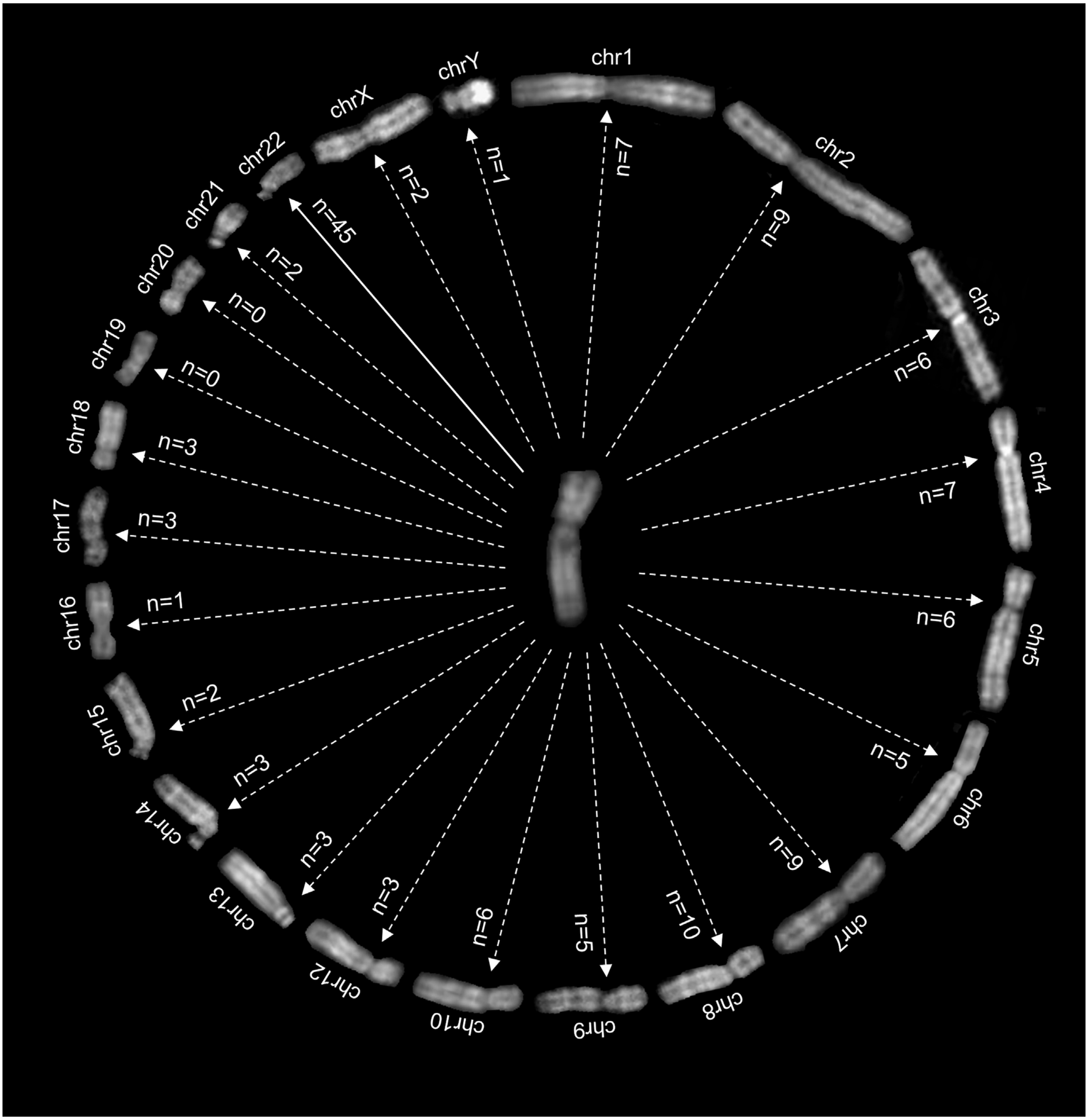


**Figure 1. Chromosome 11 translocation predisposition**
Chromosomes in QFQ bands are arranged in a circle, with chromosome 11 in the middle. The arrows indicate the number of translocations collected in this specific study.

of chromosome 11 cytogenetic bands in the hg19 reference from the UCSC genome browser. In particular, we classify those that have the chromosome 11 breakpoint located in a cytoband containing at least one OR gene from those that have the chromosome 11 breakpoints located in a cytoband without OR genes. We excluded 45 cases of recurrent translocations involving the 11q23 cytoband and also cases for which a lack of information about the sub-band hampered the assignment to either group. Our final dataset consisted of 62 translocations: 15 with chromosome 11 breakpoints mapped to an "OR cytoband" and 47 with chromosome 11 breakpoints mapped to a "no OR cytoband." This collection did not show an enrichment of breakpoints in OR cytobands (Fisher's exact test $p = 0.14$, odds ratio $= 0.63$, two-sided). Unfortunately, information on familiarity was available in about 50% cases (Table S1). Maternal transmission was observed to be twice as high as paternal transmission, and the rate of new mutations was not negligible. Given that physical interaction and pairing of double-strand breaks (DSBs) appear to be

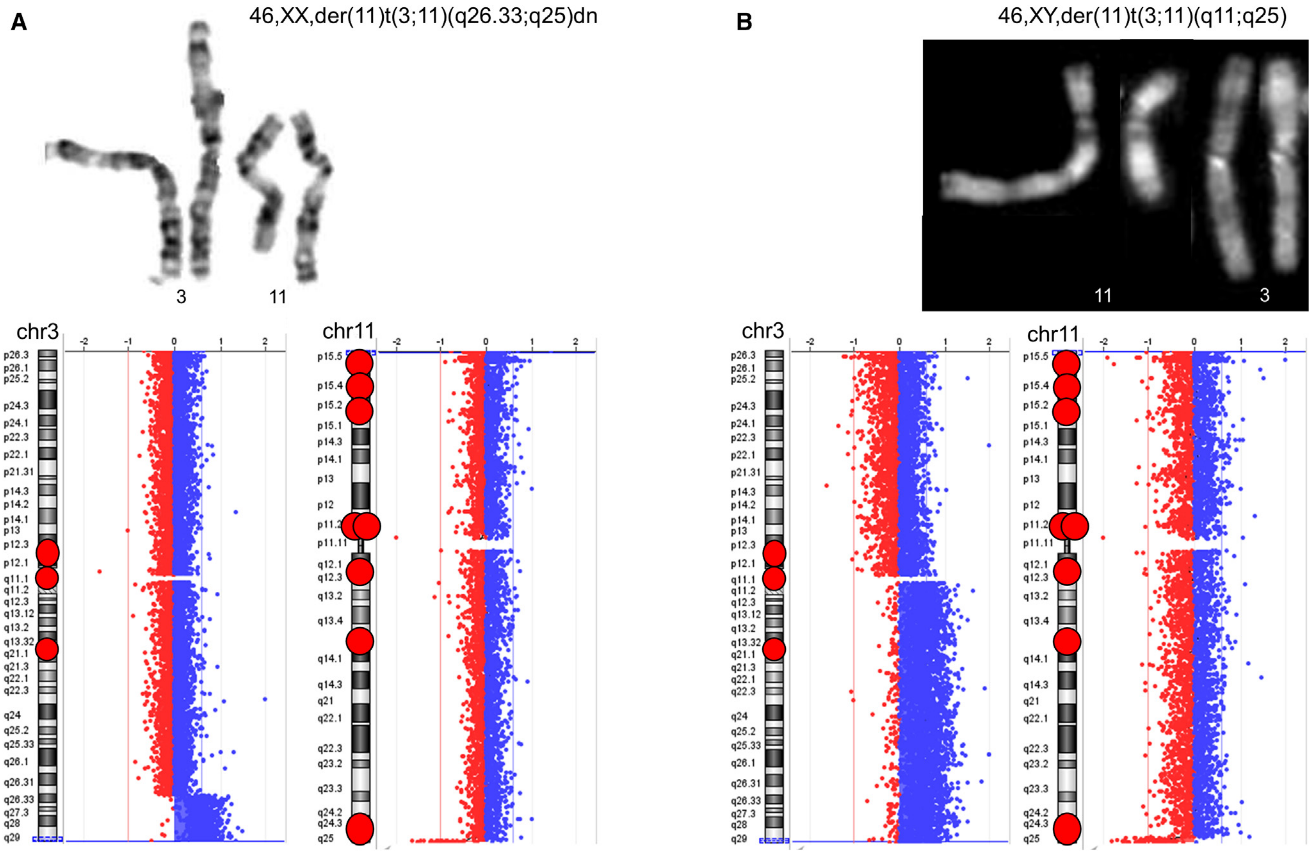


**Figure 2. Two examples of translocation derivatives**
In both cases, the partner of chromosome 11 is chromosome 3. In (A), the breakpoint on chromosome 3 falls in a cytoband devoid of OR gene clusters; however, in (B), a OR gene cluster is present in the cytoband of the breakpoint of chromosome 3. The chromosome 11 breakpoints fall in the same cytoband containing OR genes, in both cases. The pairs of chromosomes (top) and their genomic profiles (bottom) are shown.

necessary for the inaccurate ligation of the two broken chromosomes,[16] chromosome 11 and its translocation partners could co-localize in the normal cells' nuclei prior to rearrangement, according to the "contact first" hypothesis that the three-dimensional genome architecture contributes to rearrangements between chromosomes showing spatial interactions.[17] Our analysis focused on the proximity of chromosomal regions between chromosome 11 and its companion chromosomes, taking into account translocations with breakpoints in cytobands 11p15 and 11q23, which account for approximately 33% all translocations studied. By using the Hi-C data visualization software (Juicebox Aiden Lab Tool[18]) of the human GM12878 cell line, we obtained a qualitative output showing the interactions between the specific 11p15 or 11q23 region and the whole genome. Then, we selected the chromosomal partner regions involved in the translocations and the proximity for most of the cytobands was inferred (see supplemental methods, Table S5, and Figures S4A–S4Z).

***Interchromosomal rearrangements: Insertions***

The only insertion case reported in this study, 46,XX,ins(11;2)(p14;q14.3q31), was identified in the karyotype of a woman, pregnant at the 13th week of gestation, whose sister was a carrier of the same anomaly (Figure S1A). The insertion event was confirmed by FISH using 11p and 11q telomeric probes. The pregnancy was normal.

***Intrachromosomal rearrangements: Inversions and deletions***

We evidenced 14 inversions: 12 pericentric and 2 paracentric (Figure 3, blue and green lines, respectively). In contrast to translocations, in virtually all but one of the 14 diagnosed inversion cases, the breakpoints were in cytobands containing OR genes.

In this study, only 2 deletions were detectable by conventional cytogenetics (Figure 3, brown lines); one concerns the p arm, *de novo*, and one the q arm. The breakpoints do not appear to be affected by OR genes, but the numbers are too low to draw any conclusions.

## CNVs detected by array-CGH

A total of 67 CNVs were detected, of which 35 were deletions and 32 were duplications, in 65 investigated patients. As a matter of fact, in two cases, a double CNV was observed: a double duplication and a duplication followed by a deletion, both on chromosome 11 (Figure S3). We next assessed the location of the 134 breakpoints of 35 deletions and 32 duplications and found that six were within

**Table 1. Distribution of the translocation breakpoints along the cytobands of chromosome 11**

| Chromosome 11 | Cytoband | OR genes | N° breakpoints | % |
|---|---|---|---|---|
| p arm | 15 | + | 15 | 16.1 |
| | 14 | − | 0 | 0.0 |
| | 13 | − | 10 | 10.7 |
| | 12 | − | 2 | 2.1 |
| | 11.2 | + | 5 | 5.4 |
| | 11.1 | + | 3 | 3.2 |
| Centromere | / | / | / | / |
| q arm | 11 | + | 5 | 5.4 |
| | 12 | − | 1 | 1.1 |
| | 13 | + | 7 | 7.5 |
| | 14 | − | 4 | 4.3 |
| | 21 | − | 9 | 9.7 |
| | 22 | − | 5 | 5.4 |
| | 23 | − | 15[a] | 16.1 |
| | 24 | + | 3 | 3.2 |
| | 25 | − | 9 | 9.7 |

Based on Glusman's mapping, OR gene families are present or absent.[4]
[a]45 recurrent translocations (11q; 22q) are not included.

10 kbp of an OR gene. We generated 1,000 permutations of the 134 breakpoints that showed a mean of 3.5 breakpoints within 10 kbp of an OR gene. While this value suggested a possible enrichment of breakpoints near OR genes, it did not reach statistical significance ($p = 0.136$). Taken together, we did not observe an enrichment of chromosome 11 rearrangement breakpoints near OR genes.

In eight cases, duplications were found together with other genomic abnormalities on other chromosomes. Also for deletions, in nine cases, they resulted in combination with a duplication and/or a further deletion in other chromosomes. Following is the description of some interesting cases.

A case of prenatal diagnosis with fetal abnormalities observed at morphological ultrasound (pregnancy from egg donation) showed a deletion: (arr[GRCh37] 11q14.1(79726995_83738395)x1) (size 4,011 kbp). Since the deletion was not present in the father, we can state that the deletion was derived from the donated egg cell. Assuming a phenotypically normal oocyte donor, we cannot attribute the pathogenicity of the fetal abnormalities to this deletion. Furthermore, in this region, no OMIM genes associated with known diseases are present.

In the double duplication case (Figure S3, case "*"), (arr [GRCh37] 11p12p11.12(38353874_51327199)x2∼3,11q 11q12.1(54829323_58291307)x2∼3), the presence of a small supernumerary marker chromosome derived from chromosome 11, in mosaic condition (40%), could only be inferred. In fact, centromeric regions are not represented by specific probes, and they cannot be visualized by array-CGH analyses. Unfortunately, patient material for FISH analysis was not available, making it impossible to test this hypothesis.

In the duplication and terminal deletion case (Figure S3, case "**"), (arr[GRCh37] 11q23.3q24.1(119982356_122568533)x3,11q24.1q25(122621163_134868407)x1), a possible inverted duplication and deletion of 11qter, or inv dup del(11qter) has been assumed, but similarly to the case described above, it was not possible to confirm.

A deletion of 1,530 kbp in 11q25 (from nt133403830 to nt134934196), in addition to a duplication of 16 Mb in 5q34q35.3, was observed in two brothers with a clinical phenotype. A possible unbalanced translocation inherited from one parent could explain the same CNVs observed in both brothers. Due to lack of subject material, it was not possible to continue the study.

Finally, in a 1-year-old girl with a clinical phenotype and normal standard karyotype (46,XX), array-CGH evidenced a pathological outcome with a duplication on chromosome 3 and a deletion on chromosome 11: arr[GRCh37] 3q26.33q29(179498992_197861598)x3,11q25(134446101_134934196)x1. This is a terminal deletion on chromosome 11, without gene involvement.

## Discussion

The identification of DNA motifs and molecular mechanisms mediating structural rearrangements in the human genome is challenging. Such identification is key for the prediction of genome breakage hot spots and structural variants and the development of targeted molecular diagnostics.[2] It is interesting to note that gene family regions and loci with genes or pseudogenes in "linked proximity" appear to be particularly prone to genomic instability, where LCR clusters might function as NAHR substrates. On the other hand, Alu elements can mediate template switching during microhomology replication-based repair mechanisms.[19] In both cases, rearrangements arise, generating CNV alleles constituted by deletions and duplications.[1,8,20,21] Given that chromosome 11 contains more than 50% all OR clusters (many of which dispersed throughout the human genome) and that recurrent reciprocal translocations like t(4;11) and t(8;12) are mediated by NAHR with an involvement of OR clusters (in particular for t(4;11)[8]), we wondered if OR clusters mediate chromosome 11 translocations. This idea, however, is not supported by statistical analysis that compares the presence of breakpoints in cytobands with or without OR, as well as analysis that compares breakpoint regions with or without OR sequences.

Interestingly, chromosome 11 synteny is highly conserved throughout mammalian evolution. In both primate and boreoeutherian mammalian ancestors, this chromosome was probably telocentric or acrocentric, with the centromere located at the orthologous human 11qter position. The current structure of human chromosome 11 was defined by a significant pericentric inversion and

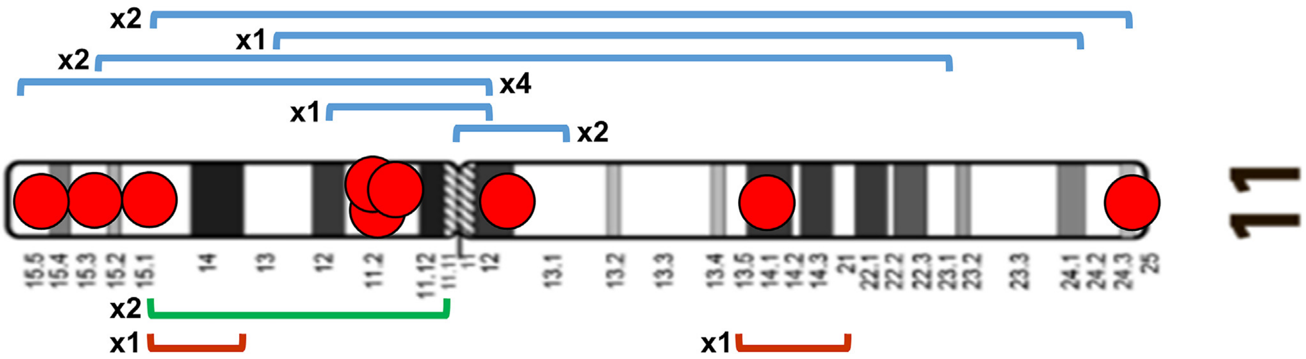


**Figure 3. Intrachromosomal rearrangements: Inversions and deletions**
Ideogram of chromosome 11, showing the breakpoints of pericentric and paracentric inversions (blue and green lines, respectively) and deletions (brown lines). Cytobands containing OR genes are indicated by red circles. The numbers indicate how many times the specific anomaly has been reported in apparently unrelated subjects.

centromere repositioning event that occurred in the common ancestor of humans and African apes.[22] The two inversion breakpoints were found at 11q13.4 and 11p15.4, the latter about 1 Mbp (million base pairs) from the 11p15.4 OR gene cluster. Interestingly, two OR genes (*OR7E12P* and *OR7E117P*) are located at the 11p15.4 inversion breakpoint (Gencode v.44). Similarly, *OR7E87P* and *OR7E4P* and *OR7E126P* and *OR7E128P* genes are located at the 11q13.4 breakpoint interval. The absence of orthologs at corresponding locations in orangutan and rhesus genomes suggests that these genes were absent in the common ape ancestor. Conversely, their presence in the chimpanzee, bonobo, and/or gorilla genomes suggests that these genes were inserted at these locations in the common ancestor of humans and African apes. However, we cannot discern whether they triggered this evolutionary rearrangement or not.

Our results are consistent with the hypothesis proposed by Chiang et al.[23] that canonical NHEJ (c-NHEJ) is the main mechanism at the basis of balanced translocations. Replication-based mechanisms such as fork stalling and template switching (FoSTeS) and MMBIR are mechanisms possibly underlying the formation of non-recurrent structural variants in humans associated with the onset of many diseases.[2,24,25] On average, about one DSB per $10^8$ bp occurs spontaneously in the genome of normal human cells, and if the repair by HR does not occur, the mechanism mainly involved in the joining of chromosome ends seems to be NHEJ.[26,27]

The visualization of the proximity between the cytobands most involved in the translocations suggests their territorial proximity. Even if we obtained only qualitative evidence, we speculate that when a break occurs, the close proximity of chromosomal territories could lead the DNA repair mechanisms generating the observed translocations. In fact, most of the cytobands involved in the translocations seem to localize in close chromosome territories. Conversely, the most common recurrent constitutional translocation, t(11;22)(q23;q11), does not show this kind of proximity of chromosome territories (Figure S4Z). For this type of aberration, the supported model would be an increased rate of cruciform structures at PATRR regions that leads to increased DSB-mediated repair via the NHEJ pathway.[15]

In conclusion, this is a retrospective, multicenter work with a large collection of specific cases on rearrangements involving chromosome 11, including translocations, inversions, deletions, duplications, and one insertion. There is no similar work in the literature, to our knowledge, with such a large collection of cases. We focused more on translocations because they are more represented in our survey and therefore also assessable from a statistical point of view. Although OR genes have been implicated in t(4;11) and t(8;12),[8] our analyses indicate that OR genes are not preferentially involved in the reciprocal translocation of chromosome 11 and its partner chromosomes. Chromosome 11 structural alterations appears to be caused by a variety of DNA motifs and mechanisms, some of which are still understudied, such as those induced by the closeness of particular chromosomal territories. Further studies aimed at sequencing breakpoint translocations should be carried out to understand the nature of the sequences involved, improve knowledge on mechanisms causing genome structural variations and possible environmental effects increasing susceptibility to genetic diseases, and provide new insights into genome evolution.

## Web Resources

Juicebox Aiden Lab Tool, http://www.aidenlab.org/juicebox/.

## Supplemental information

It can be found online at https://doi.org/10.1016/j.xhgg.2023.100261.

## Acknowledgments

This work was supported by the H2020 project and Instand-NGS4PT - Integrated and standardized NGS workflows for personalized therapy.

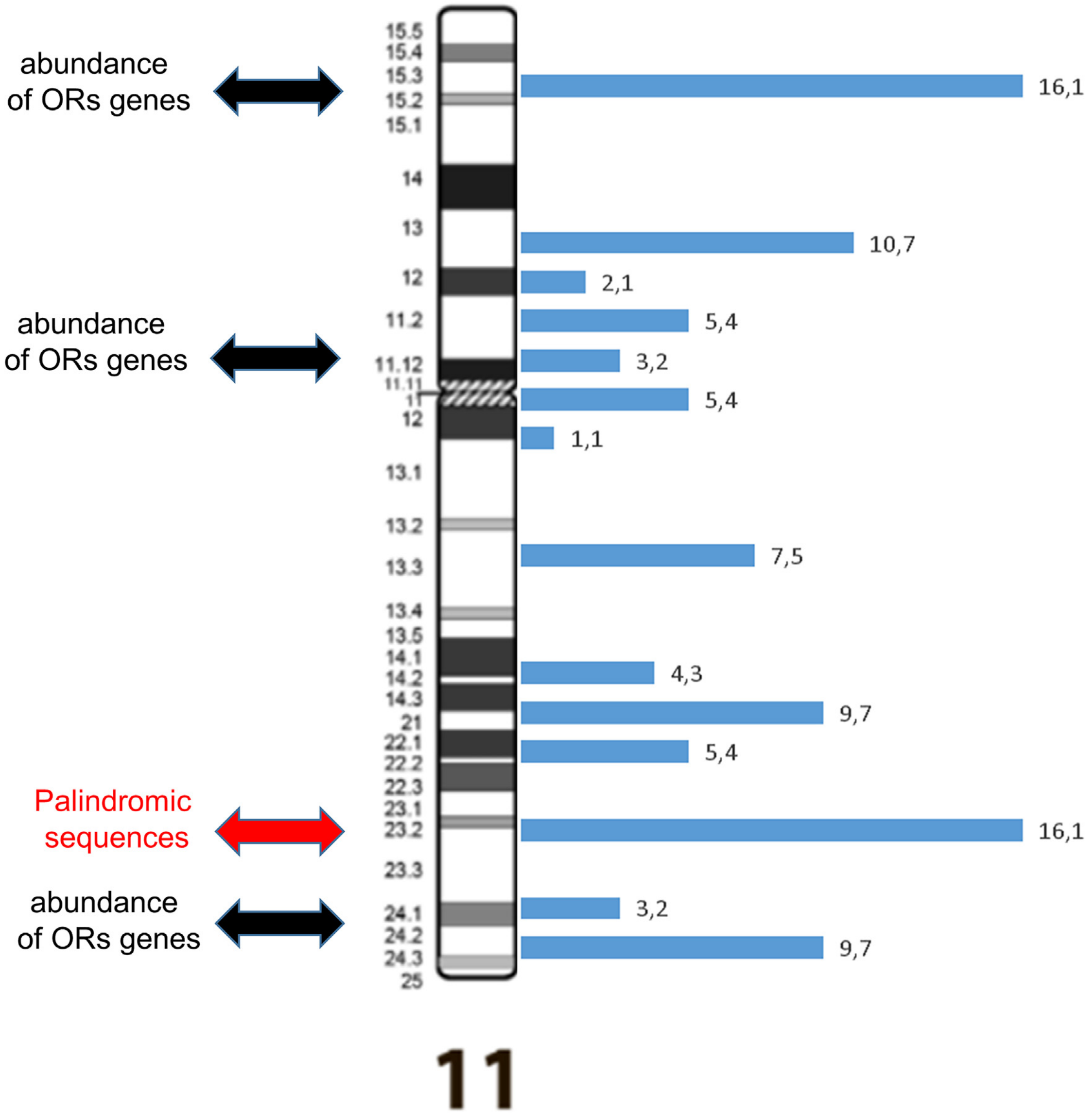


**Figure 4. Distribution of the breakpoints on chromosome 11**
The number reported on the right of the ideogram represent the percentage associated with the specific translocation from Table 1.

## Author contributions

Conceptualization, L.D., P.R., and A.B.; data curation, S.R., F.R.G., V.T., G.G., and D.C.; investigation, S.R., F.R.G., M.P.R., E.S., N.V., F.C., F.M., V.Z., E.R., O.R., C.V., I.C., L.R., and E.M.; methodology, S.R. and L.D.; resources, G.R. and M.L.; supervision, L.D., P.R., and A.B.; writing – original draft, V.T., G.G., L.D., and A.B.; writing – review & editing, S.R., D.C., L.D., P.R., and A.B. All authors have read and agreed to the published version of the manuscript.

## Declaration of interests

At the time of data collection, F.R.G. was, together with F.M., V.Z., and E.R., a full-time employee of TOMA Advanced Biomedical Assays S.p.A. (Impact Lab) without ownership shares. F.R.G. is currently a full-time employee of Menarini Silicon Biosystems, Reproductive Precision Medicine Unit.

Received: September 15, 2023
Accepted: December 28, 2023

## References

1. Lupski, J.R. (2021). Clan genomics: From OMIM phenotypic traits to genes and biology. Am. J. Med. Genet. *185*, 3294–3313.
2. Carvalho, C.M.B., and Lupski, J.R. (2016). Mechanisms underlying structural variant formation in genomic disorders. Nat. Rev. Genet. *17*, 224–238.
3. Trask, B.J., Massa, H., Brand-Arpon, V., Chan, K., Friedman, C., Nguyen, O.T., Eichler, E., van den Engh, G., Rouquier, S., Shizuya, H., and Giorgi, D. (1998). Large multi-chromosomal duplications encompass many members of the olfactory receptor gene family in the human genome. Hum. Mol. Genet. *7*, 2007–2020.
4. Glusman, G., Yanai, I., Rubin, I., and Lancet, D. (2001). The complete human olfactory subgenome. Genome Res. *11*, 685–702.
5. Deloukas, P., Matthews, L.H., Ashurst, J., Burton, J., Gilbert, J.G., Jones, M., Stavrides, G., Almeida, J.P., Babbage, A.K.,

Bagguley, C.L., et al. (2001). The DNA sequence and comparative analysis of human chromosome 20. Nature *414*, 865–871.
6. Skaletsky, H., Kuroda-Kawaguchi, T., Minx, P.J., Cordum, H.S., Hillier, L., Brown, L.G., Repping, S., Pyntikova, T., Ali, J., Bieri, T., et al. (2003). The male-specific region of the human Y chromosome is a mosaic of discrete sequence classes. Nature *423*, 825–837.
7. Ormundo, L.F., Machado, C.F., Sakamoto, E.D., Simões, V., and Armelin-Correa, L. (2020). LINE-1 specific nuclear organization in mice olfactory sensory neurons. Mol. Cell. Neurosci. *105*, 103494.
8. Ou, Z., Stankiewicz, P., Xia, Z., Breman, A.M., Dawson, B., Wiszniewska, J., Szafranski, P., Cooper, M.L., Rao, M., Shao, L., et al. (2011). Observation and prediction of recurrent human translocations mediated by NAHR between nonhomologous chromosomes. Genome Res. *21*, 33–46.
9. Giglio, S., Calvari, V., Gregato, G., Gimelli, G., Camanini, S., Giorda, R., Ragusa, A., Guerneri, S., Selicorni, A., Stumm, M., et al. (2002). Heterozygous submicroscopic inversions involving olfactory receptor-gene clusters mediate the recurrent t(4;8)(p16;p23) translocation. Am. J. Hum. Genet. *71*, 276–285.
10. Olender, T., Feldmesser, E., Atarot, T., Eisenstein, M., and Lancet, D. (2004). The olfactory receptor universe–from whole genome analysis to structure and evolution. Genet. Mol. Res. *3*, 545–553.
11. Redaelli, S., Conconi, D., Sala, E., Villa, N., Crosti, F., Roversi, G., Catusi, I., Valtorta, C., Recalcati, M.P., Dalprà, L., et al. (2022). Characterization of Chromosomal Breakpoints in 12 Cases with 8p Rearrangements Defines a Continuum of Fragility of the Region. Int. J. Mol. Sci. *23*, 3347.
12. Edelmann, L., Spiteri, E., Koren, K., Pulijaal, V., Bialer, M.G., Shanske, A., Goldberg, R., and Morrow, B.E. (2001). AT-rich palindromes mediate the constitutional t(11;22) translocation. Am. J. Hum. Genet. *68*, 1–13.
13. Quinlan, A.R., and Hall, I.M. (2010). BEDTools: a flexible suite of utilities for comparing genomic features. Bioinformatics *26*, 841–842.
14. Team, R.C. (2020). A Language and Environment for Statistical Computing (R Foundation for Statistical Computing).
15. Kurahashi, H., Inagaki, H., Ohye, T., Kogo, H., Tsutsumi, M., Kato, T., Tong, M., and Emanuel, B.S. (2010). The constitutional t(11;22): implications for a novel mechanism responsible for gross chromosomal rearrangements. Clin. Genet. *78*, 299–309.
16. Roukos, V., and Misteli, T. (2014). The biogenesis of chromosome translocations. Nat. Cell Biol. *16*, 293–300.
17. Engreitz, J.M., Agarwala, V., and Mirny, L.A. (2012). Three-dimensional genome architecture influences partner selection for chromosomal translocations in human disease. PLoS One *7*, e44196.
18. Robinson, J.T., Turner, D., Durand, N.C., Thorvaldsdóttir, H., and Mesirov, J.P. (2018). Juicebox.js provides a cloud-based visualization system for Hi-C data. Cell Syst. *6*, 256–258.e1.
19. Song, X., Beck, C.R., Du, R., Campbell, I.M., Coban-Akdemir, Z., Gu, S., Breman, A.M., Stankiewicz, P., Ira, G., Shaw, C.A., and Lupski, J.R. (2018). Predicting human genes susceptible to genomic instability associated with. Genome Res. *28*, 1228–1242.
20. Lupski, J.R. (1998). Genomic disorders: structural features of the genome can lead to DNA rearrangements and human disease traits. Trends Genet. *14*, 417–422.
21. Stankiewicz, P., and Lupski, J.R. (2002). Genome architecture, rearrangements and genomic disorders. Trends Genet. *18*, 74–82.
22. Cardone, M.F., Lomiento, M., Teti, M.G., Misceo, D., Roberto, R., Capozzi, O., D'Addabbo, P., Ventura, M., Rocchi, M., and Archidiacono, N. (2007). Evolutionary history of chromosome 11 featuring four distinct centromere repositioning events in Catarrhini. Genomics *90*, 35–43.
23. Chiang, C., Jacobsen, J.C., Ernst, C., Hanscom, C., Heilbut, A., Blumenthal, I., Mills, R.E., Kirby, A., Lindgren, A.M., Rudiger, S.R., et al. (2012). Complex reorganization and predominant non-homologous repair following chromosomal breakage in karyotypically balanced germline rearrangements and transgenic integration. Nat. Genet. *44*, 390–397. S1–S391.
24. Stankiewicz, P., and Lupski, J.R. (2010). Structural variation in the human genome and its role in disease. Annu. Rev. Med. *61*, 437–455.
25. Abyzov, A., Li, S., Kim, D.R., Mohiyuddin, M., Stütz, A.M., Parrish, N.F., Mu, X.J., Clark, W., Chen, K., Hurles, M., et al. (2015). Analysis of deletion breakpoints from 1,092 humans reveals details of mutation mechanisms. Nat. Commun. *6*, 7256.
26. Vilenchik, M.M., and Knudson, A.G. (2003). Endogenous DNA double-strand breaks: production, fidelity of repair, and induction of cancer. Proc. Natl. Acad. Sci. USA *100*, 12871–12876.
27. Mehta, A., and Haber, J.E. (2014). Sources of DNA double-strand breaks and models of recombinational DNA repair. Cold Spring Harbor Perspect. Biol. *6*, a016428.

**HGGA, Volume *5***

## Supplemental information

# Olfactory receptor genes and chromosome 11 structural aberrations: Players or spectators?

**Serena Redaelli, Francesca Romana Grati, Viviana Tritto, Giuliana Giannuzzi, Maria Paola Recalcati, Elena Sala, Nicoletta Villa, Francesca Crosti, Gaia Roversi, Francesca Malvestiti, Valentina Zanatta, Elena Repetti, Ornella Rodeschini, Chiara Valtorta, Ilaria Catusi, Lorenza Romitti, Emanuela Martinoli, Donatella Conconi, Leda Dalprà, Marialuisa Lavitrano, Paola Riva, and Angela Bentivegna**

**Supplemental Information**

**Table of contents**

Figure S1 (A-F). Examples of identified alterations in this work.

Figure S2 (A-G). Breakpoint distribution of chromosome translocations in relation to OR gene cluster locations.

Figure S3. Copy number variations detected by array CGH.

Figure S4. Hi-C heat maps showing inter-chromosomal interactions between chromosome 11 and its partner chromosomes in translocations.

Table S1. Sex distribution of patients showing chromosome 11 anomalies identified by conventional chromosome analysis.

Table S2. Sex distribution of patients showing chromosome 11 anomalies identified by array-CGH.

Table S3. Inheritance of translocation.

Table S4. Distribution of translocation breakpoints on chromosome 11 partners.

Table S5. Cytobands involved in the main collected translocations, visualized on the Hi-C maps.

Supplemental Methods.

1) Array Comparative Genomic Hybridization (Array-CGH).

2) Juicebox Aiden Lab Tool.

**Figure S1. Examples of identified alterations in this work.**

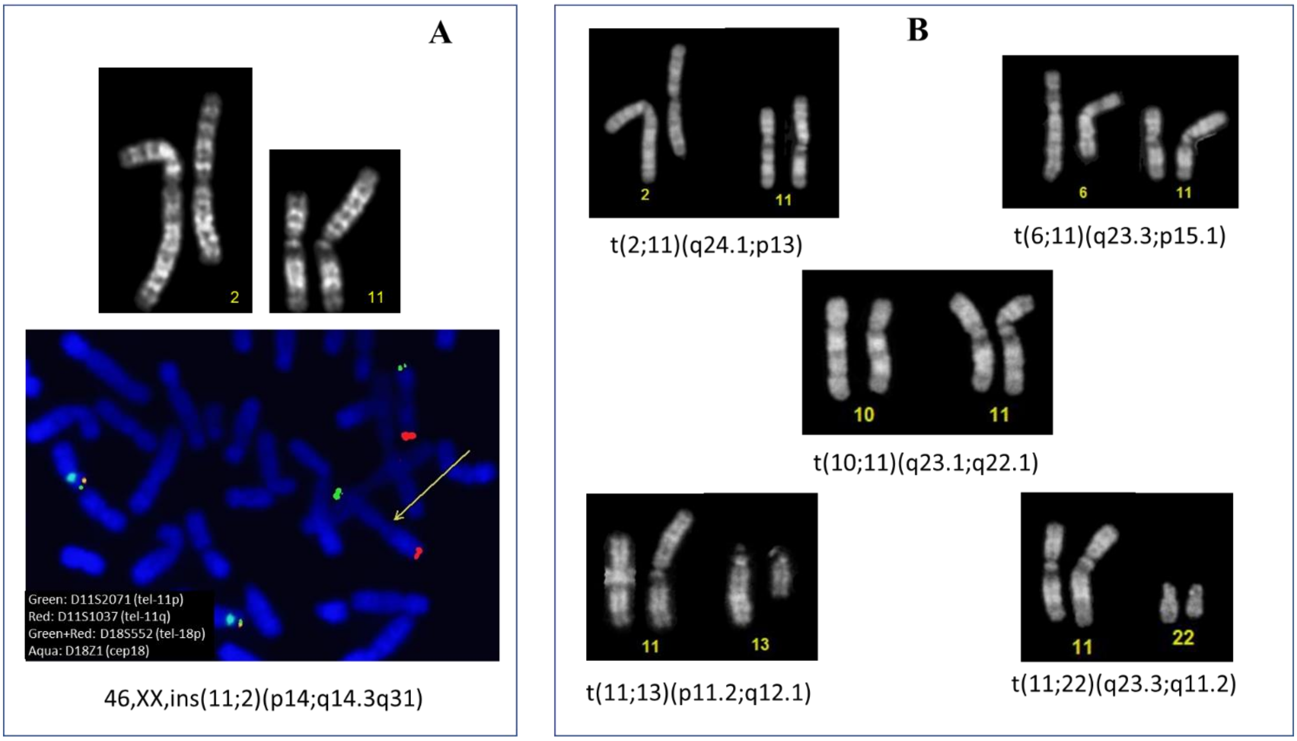


**Figure S1. A.** The unique insertion case: part of the long arm of chromosome 2 is inserted into the short arm of chromosome 11. Top: QFQ banded chromosomes 2 and 11 (normal ones on the left of each couple). Bottom: FISH with Vysis ToTelVysion Multi-Color FISH Probe Kit. Probes that recognize the specific telomeric regions of 11 (green= tel 11p; red= tel 11q) hybridize in the correct position, as can be observed in the partial metaphase. The arrow indicates the inserted chromosome. (red+green= tel 18p; aqua=CEP18). FISH analysis was performed with chromosome 11 probes and not with the whole kit.

**Figure S1. B.** QFQ banded chromosomes from cases of translocation involving chromosome 11 identified in this collection. The abnormal chromosomes are on the right of each couple.

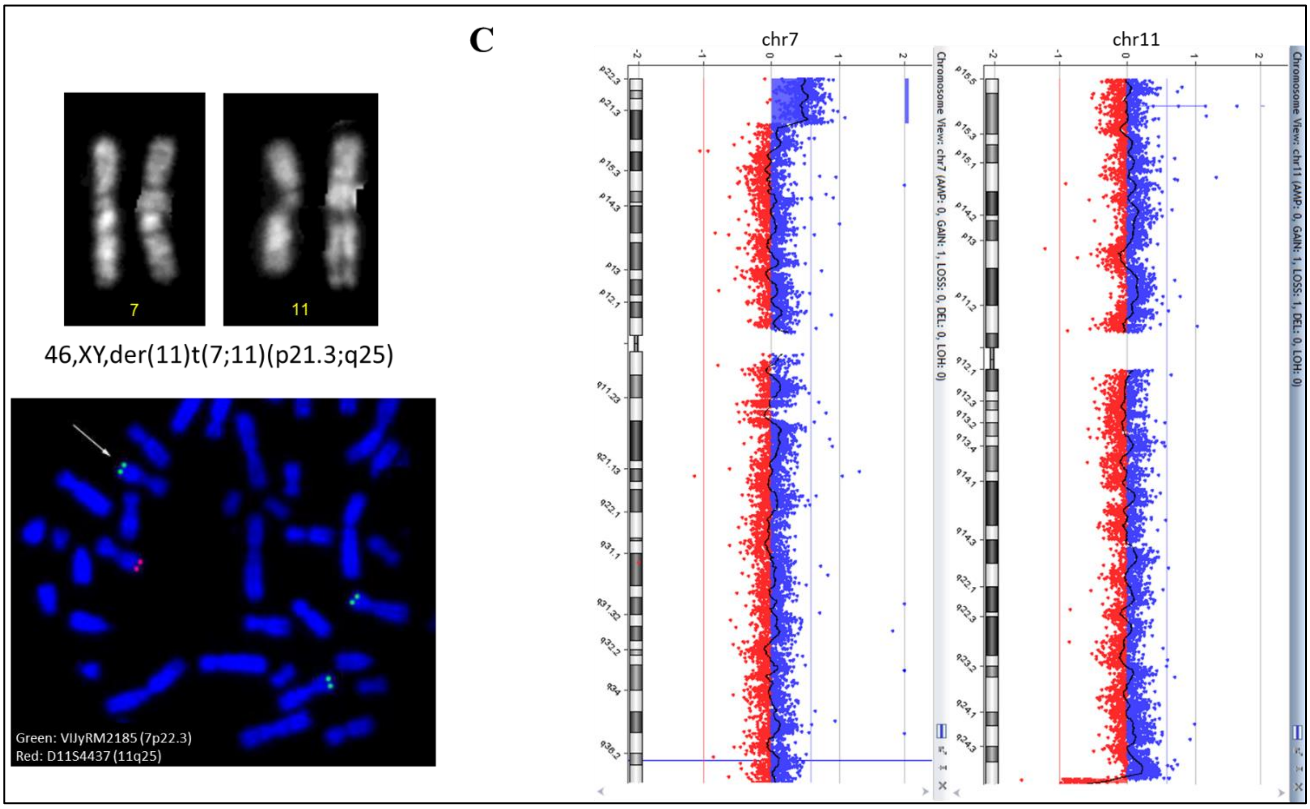


**Figure S1. C**. A derivative of translocation 7;11 evidenced by QFQ banding, FISH, and array-CGH. Top left: QFQ banded chromosomes with the derivative 7 on the right and the derivative chromosome 11 on the left, respectively. Bottom left: the partial metaphase shows FISH with a specific probe for 7p telomere, (7p22.3, VIJyRM2185, green) and a specific probe for 11q telomere, (11q25, D11S4437, red). Three green signals are observed, of which two are correctly positioned in telomeres 7p and one positioned on the derivative 11q (arrow). Normal chromosome 11 shows a 11q red signal on the qter. Right: array-CGH chromosome view of the two chromosomes involved in the translocation.

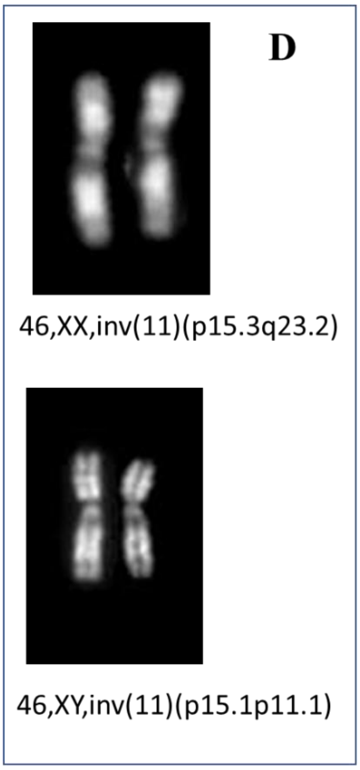


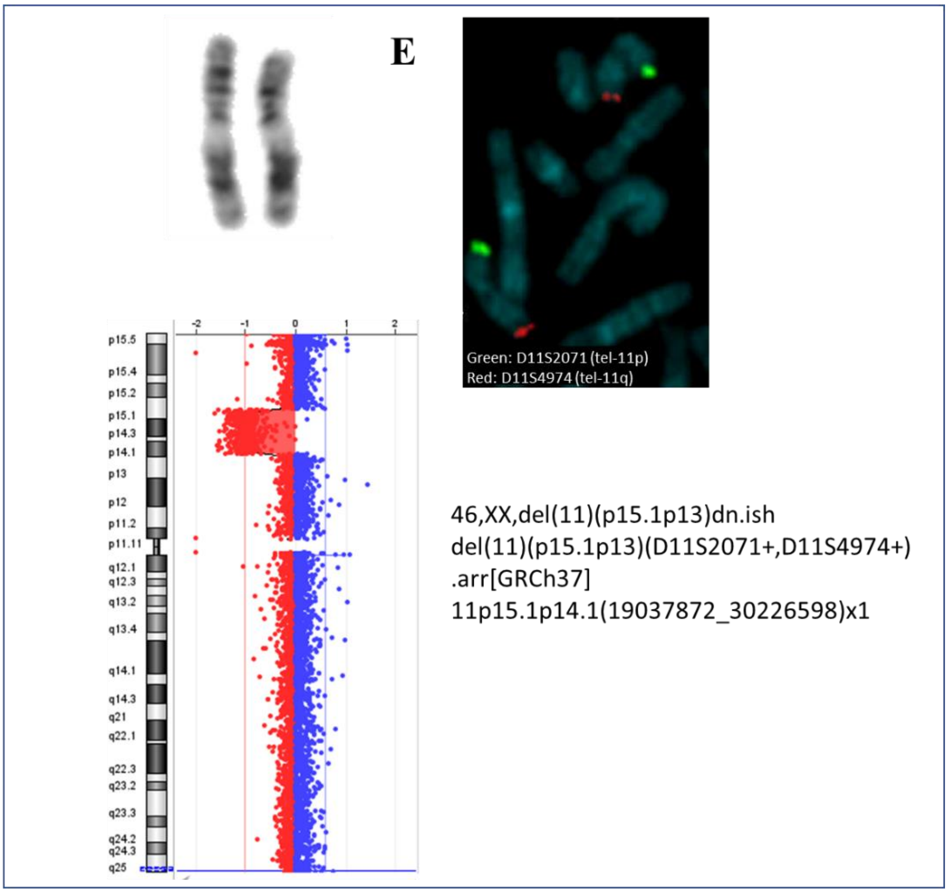


**Figure S1. D.** Top: a pericentric inversion observed in QFQ bands (the abnormal chromosome 11 is on the right of the couple); bottom: a paracentric inversion in QFQ bands (the abnormal chromosome 11 is on the left of the couple).

**Figure S1. E.** A 11p interstitial deletion identified by conventional cytogenetics (top) and confirmed by array-CGH (bottom). On the left: Chromosomes 11 are shown in GTG bands (the abnormal chromosome 11 is on the right of the couple). On the right: FISH with 11 specific telomeric probes shows normal signals (tel-11p, D11S2071, green; tel-11q, D11S4974, red).

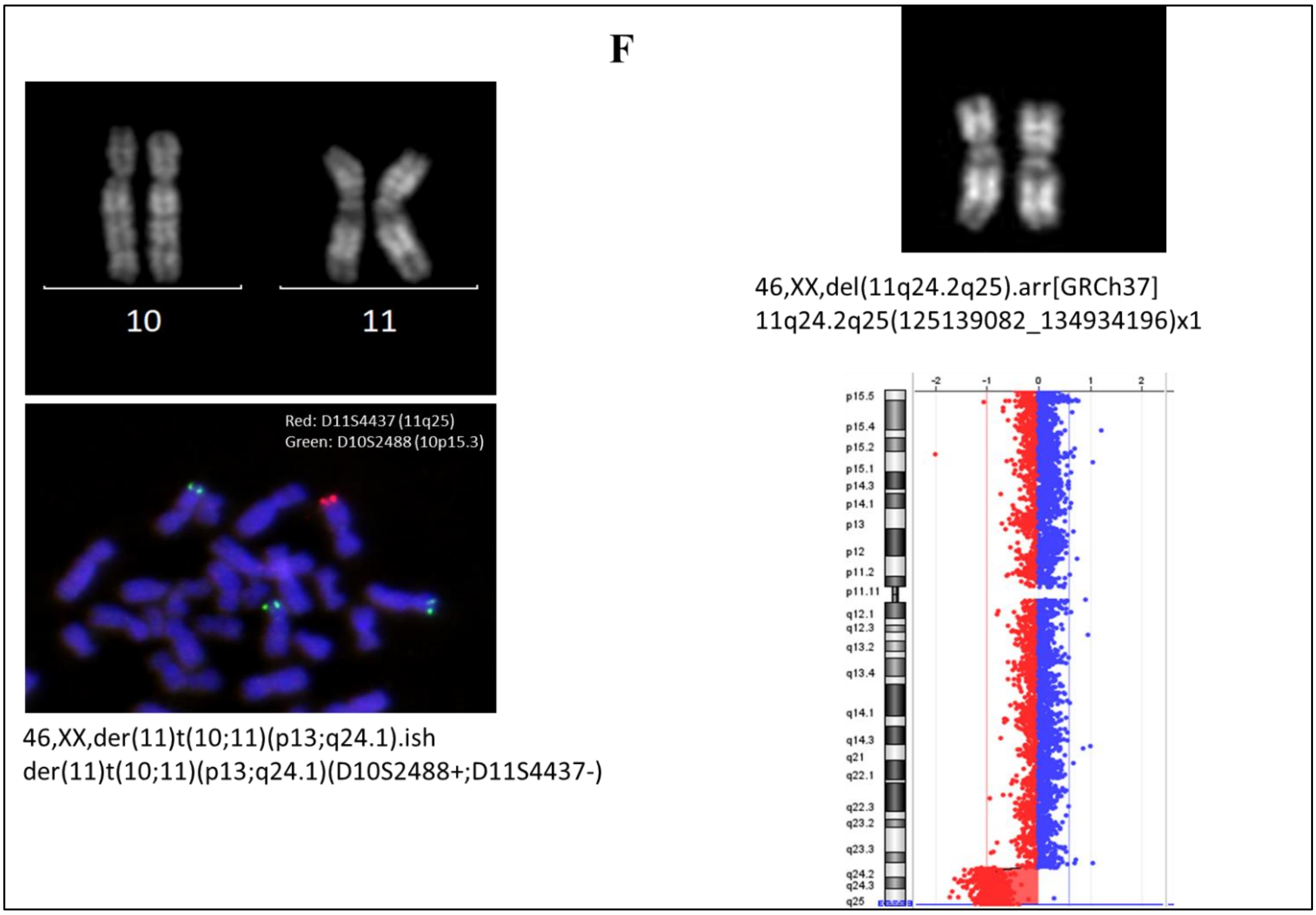


**Figure S1. F.** Two examples of chromosome alterations not detected by conventional cytogenetics. QFQ banding of chromosomes do not show any alterations in both cases (top panels). On the left, an unbalanced translocation with a derivative identified by array-CGH and confirmed by FISH. The bottom left panel shows a partial metaphase with three green signals (10p15.3, D10S2488) and one red signal (11q25, D11S4437), confirming the imbalance of the translocation. On the right, a terminal deletion identified by array-CGH (bottom).

**Figure S2. Breakpoint distribution of chromosome translocations in relation to OR gene cluster locations.**

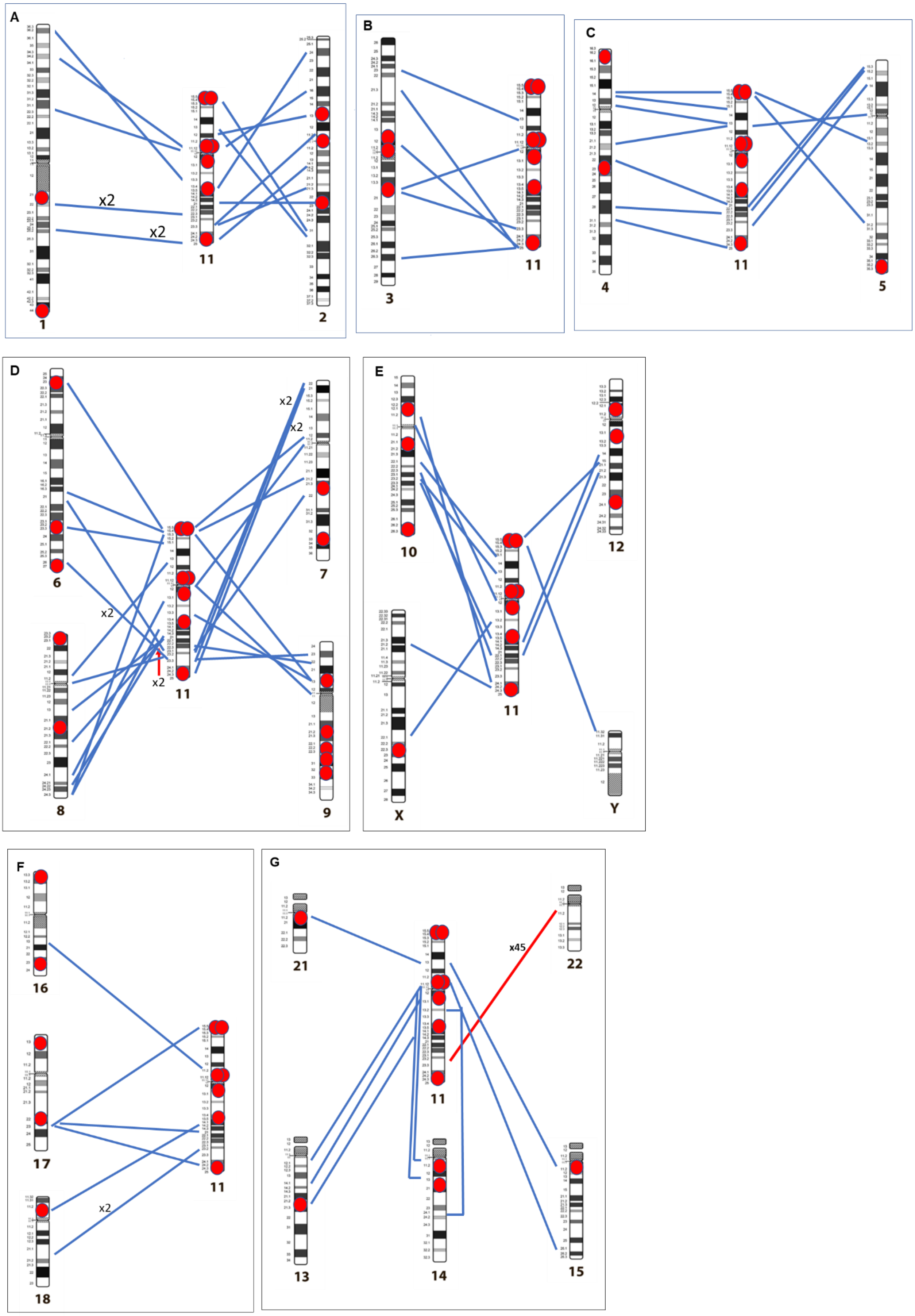

**Figure S2. A-G.** Breakpoint distribution of chromosome translocations in relation to OR gene cluster locations based on Glusman's mapping (red circles; see ref. 4 in the main text). Blue lines indicate bands involved in translocations with chromosome 11; the recurrent 11q;22q translocation is represented by a red line. “x N°” (for example x2): indicates the number of times the rearrangement is identified. See Table S4 for details.

**Figure S3. Copy number variations detected by array CGH.**

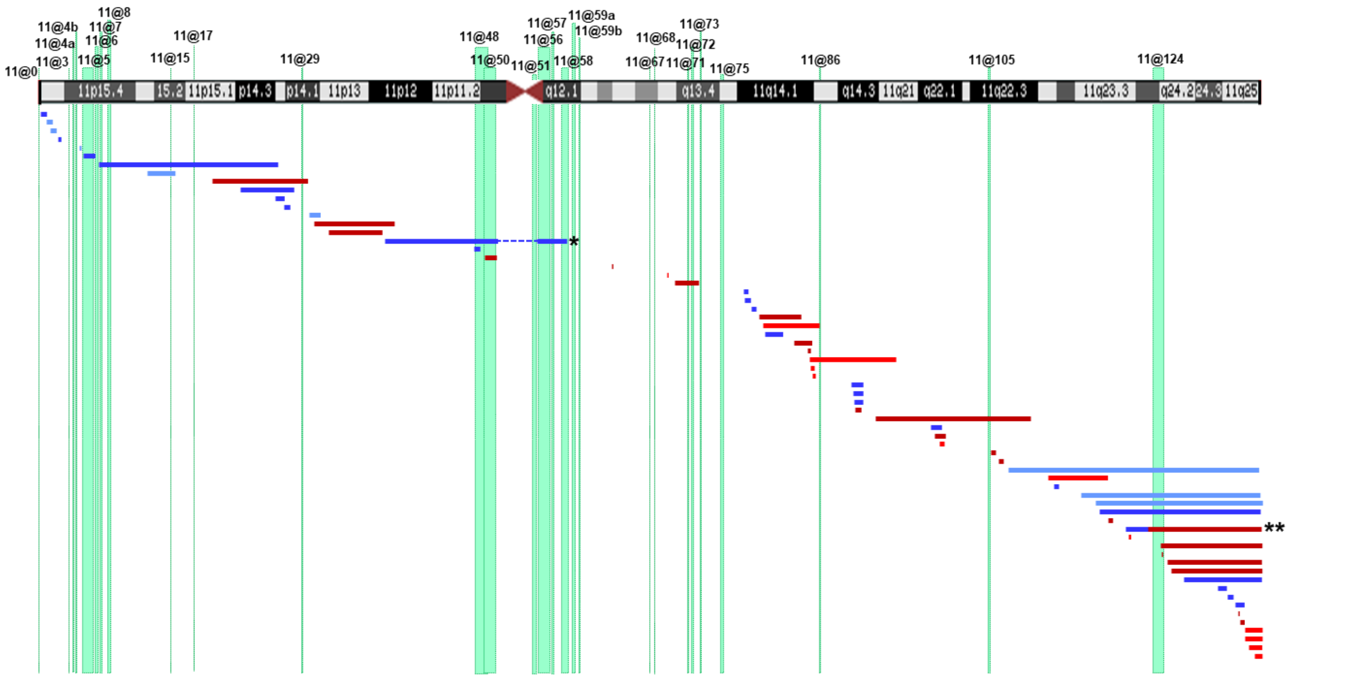


- duplication on chromosome 11
- duplication on chromosome 11 in a patient who shows a copy number alteration also on a different chromosome
- deletion on chromosome 11
- deletion on chromosome 11 in a patient who shows a copy number alteration also on a different chromosome

* patient with a supernumerary marker derived from chromosome 11, dashed line shows centromeric region (no centromeric locus specific probes in CGH-microarray format)

** duplication followed by consecutive deletion in the same patient

OR (Olfactory Receptors) gene clusters, mapped by http://bioinfo.weizmann.ac.il/HORDE

**Figure S3.** Copy number variations detected by array CGH. 11@N stands for OR gene cluster on chromosome 11 and N is the position in Mbp.

**Figure S4. Hi-C heat maps showing inter-chromosomal interactions between chromosome 11 and its partner chromosomes in translocations.** **(a)** chr11 vs chr2; **(b)** chr11 vs chr4; **(c)** chr11 vs chr5; **(d)** chr11 vs chr5; **(e)** chr11 vs chr6; **(f)** chr11 vs chr6; **(g)** chr11 vs chr7; **(h)** chr11 vs chr7; **(i)** chr11 vs chr8; **(j)** chr11 vs chr9; **(k)** chr11 vs chr12; **(l)** chr11 vs chr17; **(m)** chr11 vs chrY; **(n)** chr11 vs chr1; **(o)** chr11 vs chr2; **(p)** chr11 vs chr2; **(q)** chr11 vs chr3; **(r)** chr11 vs chr5; **(s)** chr11 vs chr6; **(t)** chr11 vs chr6; **(u)** chr11 vs chr7; **(v)** chr11 vs chr7; **(w)** chr11 vs chr8; **(x)** chr11 vs chr9; **(y)** chr11 vs chr18; **(z)** chr11 vs chr22. Each panel obtained using Juicebox Aiden Lab Tool (http://www.aidenlab.org/juicebox/) shows the selected Hi-C dataset, the cell line on which the experiment was carried out (GM12878), the resolution (250 or 500 kb), and the genomic coordinates of the two chromosomes displayed. On the x-axis is shown one of the two partner chromosomes, on the y-axis the other one. The color scale from white to red indicates an increasing number of inter-chromosomal interactions. The maximum value of interactions detected in each investigated chromosome pair is shown and graphically represented by red. The map region showing the interactions between the two cytobands containing translocation breakpoints is boxed in black, and the respective chromosomal coordinates are reported.

a

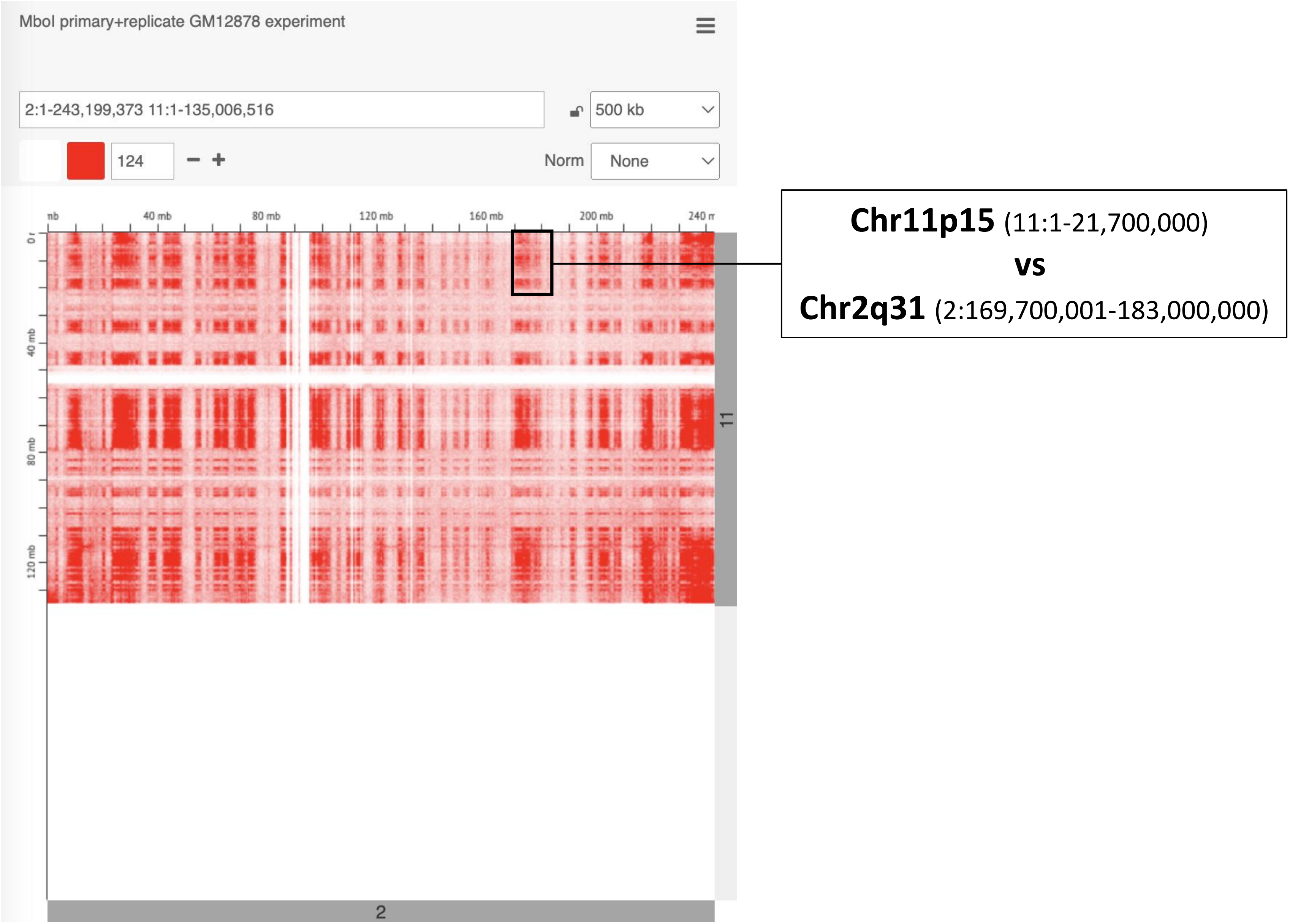
MboI primary+replicate GM12878 experiment
2:1-243,199,373 11:1-135,006,516
500 kb
124
Norm
None
40 mb
80 mb
120 mb
160 mb
200 mb
40 mb
80 mb
120 mb
11
2
Chr11p15 (11:1-21,700,000)
vs
Chr2q31 (2:169,700,001-183,000,000)

b

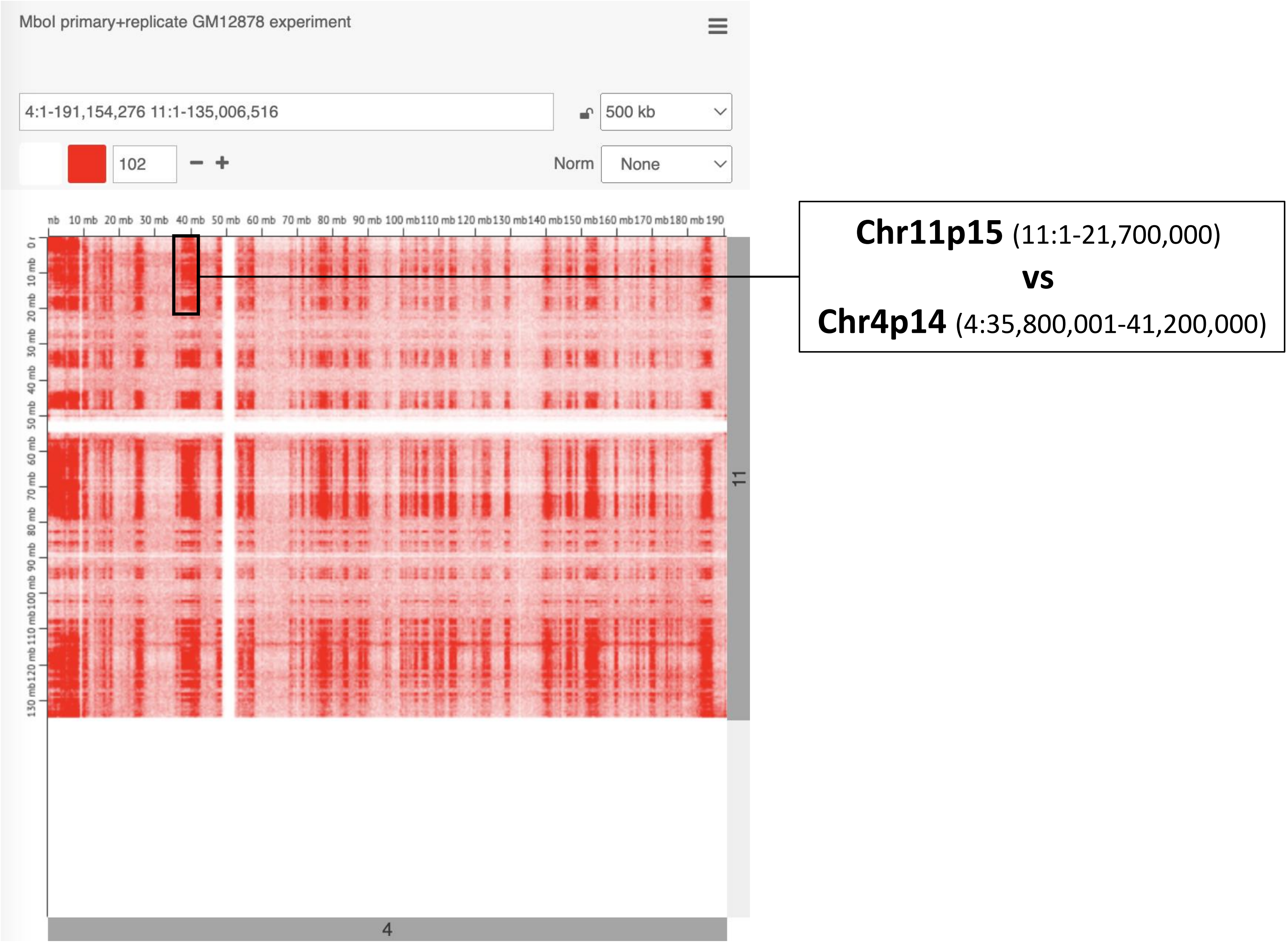
MboI primary+replicate GM12878 experiment
4:1-191,154,276 11:1-135,006,516
500 kb
102
Norm
None
11
4
Chr11p15 (11:1-21,700,000)
vs
Chr4p14 (4:35,800,001-41,200,000)

c

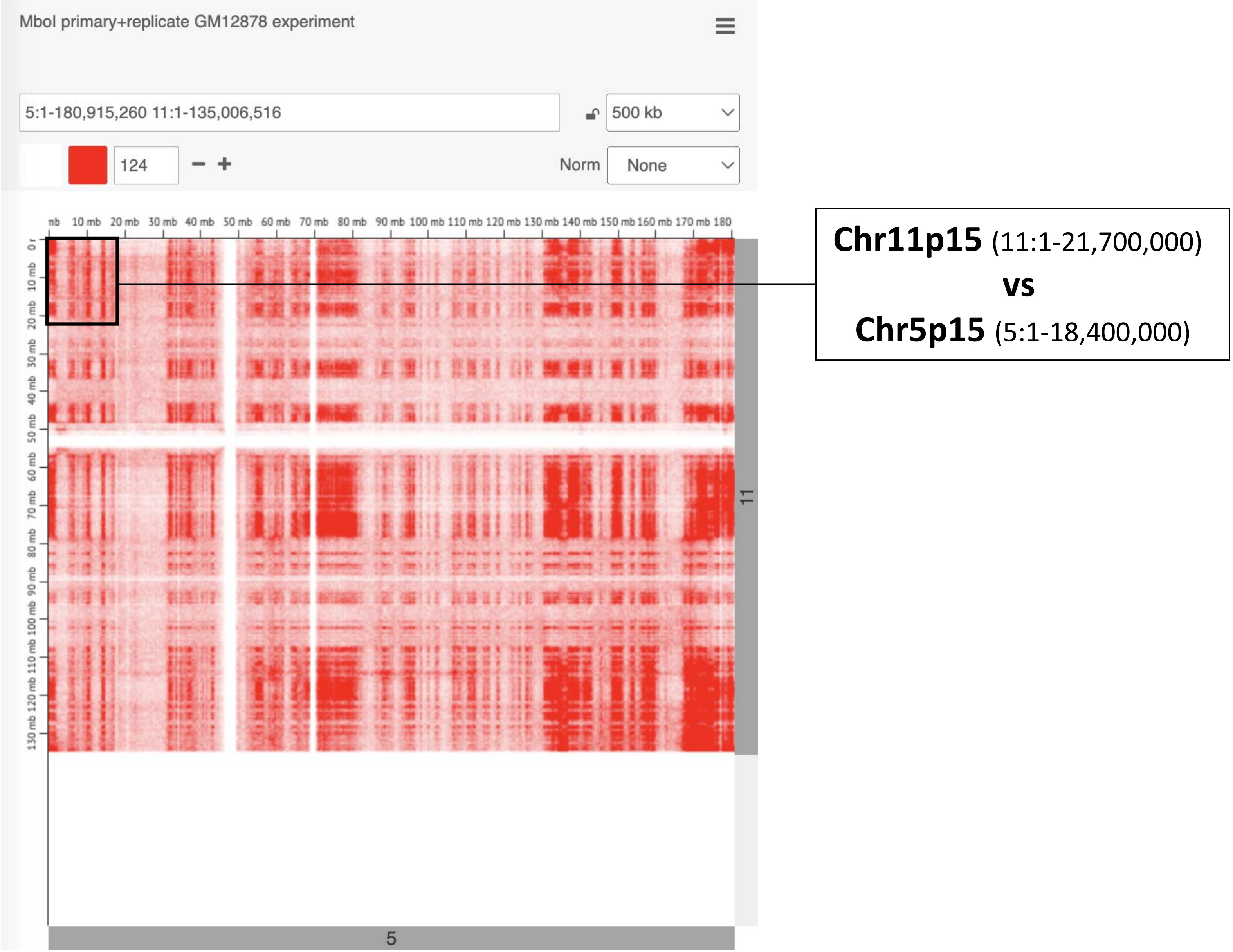

MboI primary+replicate GM12878 experiment
5:1-180,915,260 11:1-135,006,516
500 kb
124
Norm
None
10 mb 20 mb 30 mb 40 mb 50 mb 60 mb 70 mb 80 mb 90 mb 100 mb 110 mb 120 mb 130 mb 140 mb 150 mb 160 mb 170 mb 180
10 mb 20 mb 30 mb 40 mb 50 mb 60 mb 70 mb 80 mb 90 mb 100 mb 110 mb 120 mb 130 mb
11
5
**Chr11p15** (11:1-21,700,000)
**vs**
**Chr5p15** (5:1-18,400,000)

d

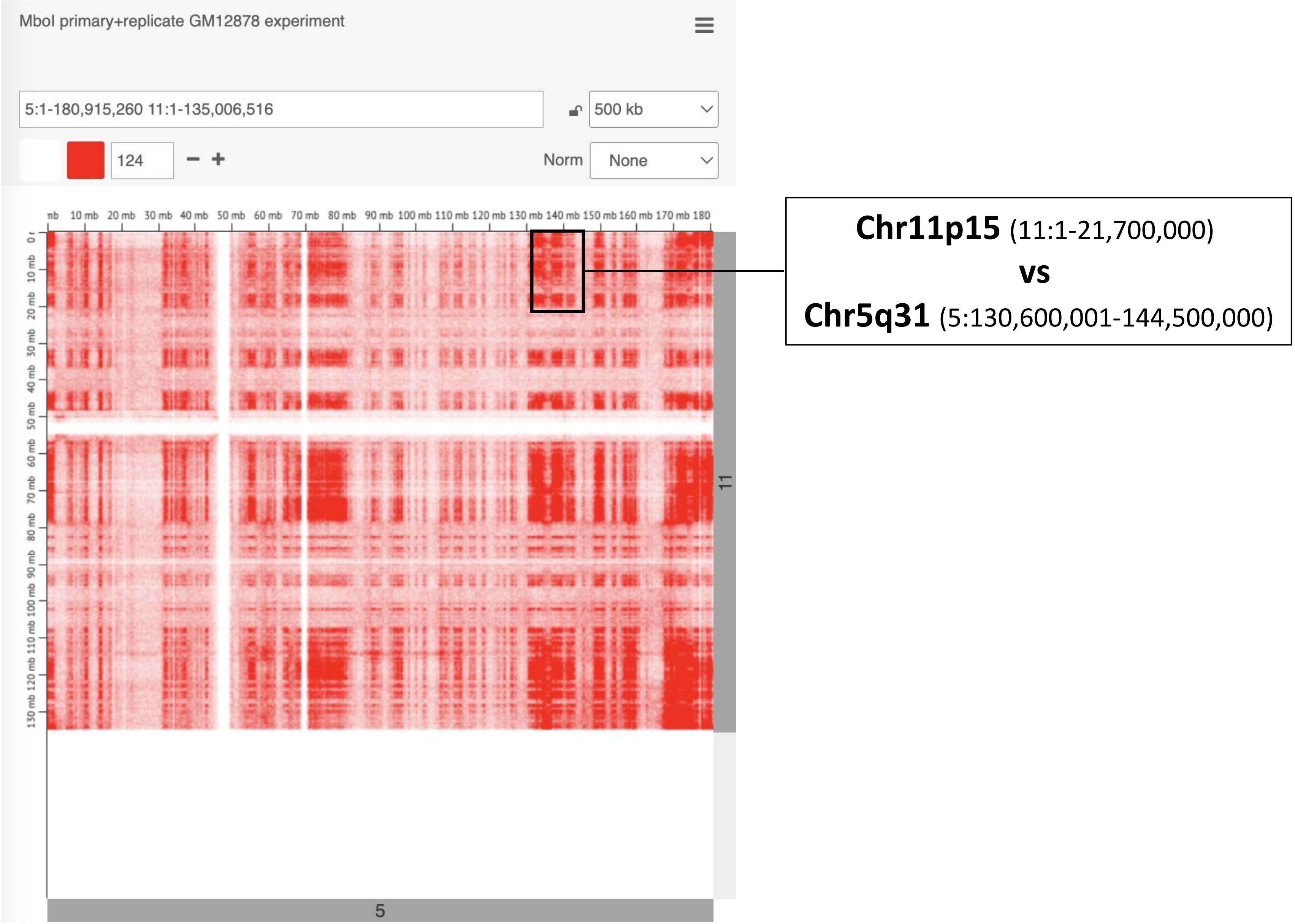

MboI primary+replicate GM12878 experiment
5:1-180,915,260 11:1-135,006,516
500 kb
124
Norm
None
11
5
Chr11p15 (11:1-21,700,000)
vs
Chr5q31 (5:130,600,001-144,500,000)

e

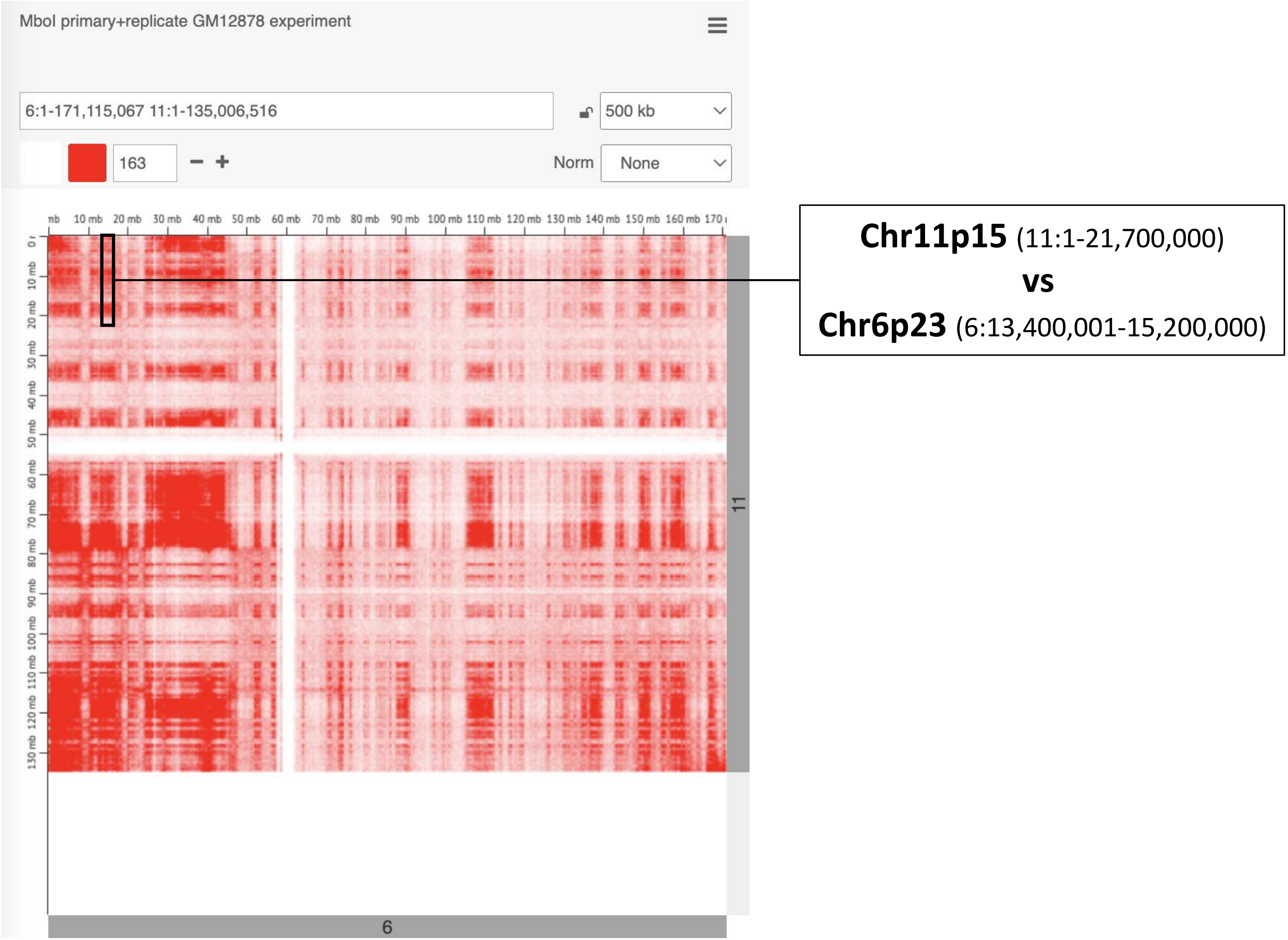
MboI primary+replicate GM12878 experiment
6:1-171,115,067 11:1-135,006,516
500 kb
163
Norm
None
10 mb
20 mb
30 mb
40 mb
50 mb
60 mb
70 mb
80 mb
90 mb
100 mb
110 mb
120 mb
130 mb
140 mb
150 mb
160 mb
11
6
Chr11p15 (11:1-21,700,000)
vs
Chr6p23 (6:13,400,001-15,200,000)

f

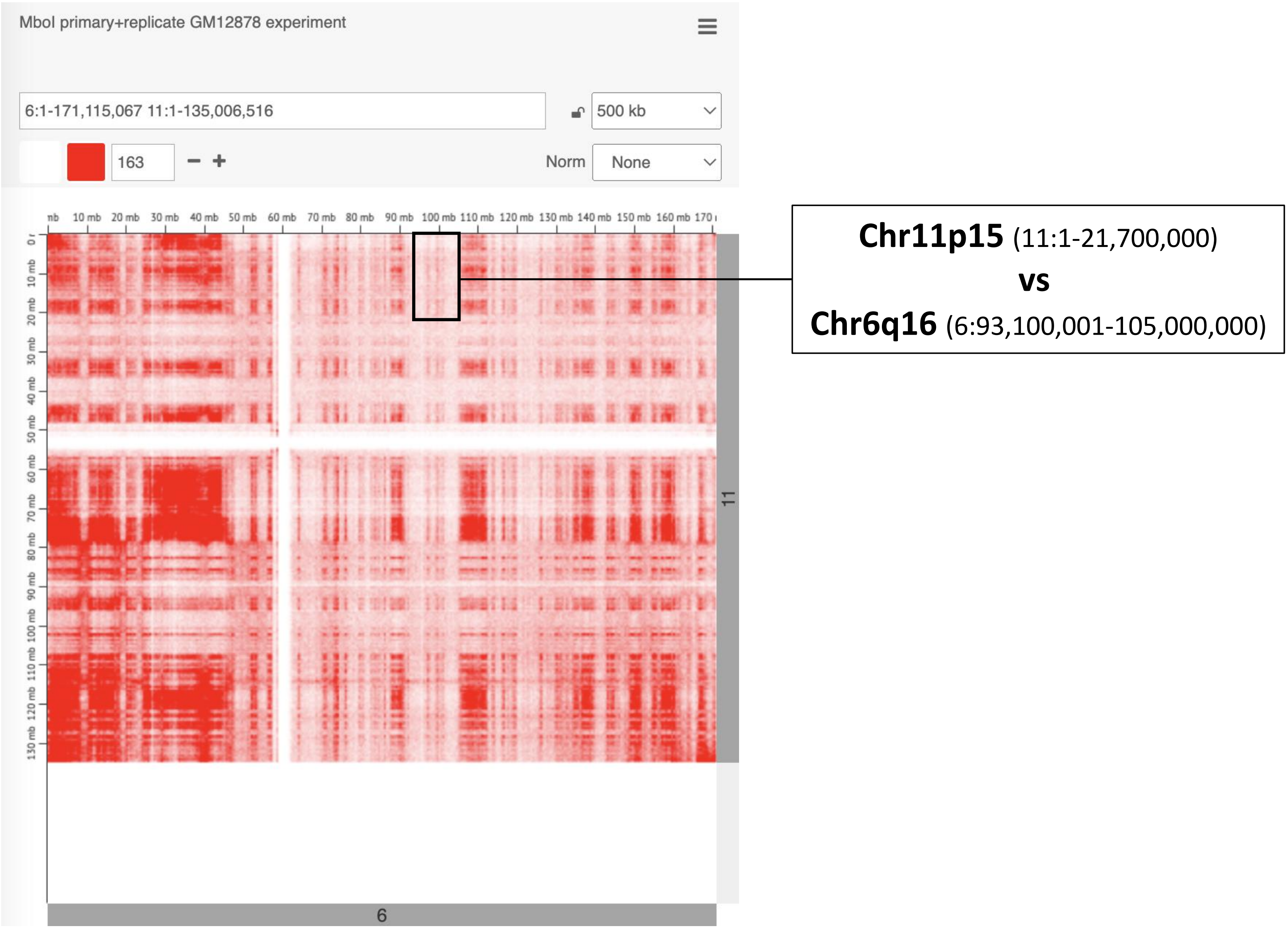
MboI primary+replicate GM12878 experiment
6:1-171,115,067 11:1-135,006,516
500 kb
163
Norm
None
Chr11p15 (11:1-21,700,000)
vs
Chr6q16 (6:93,100,001-105,000,000)
11
6

g

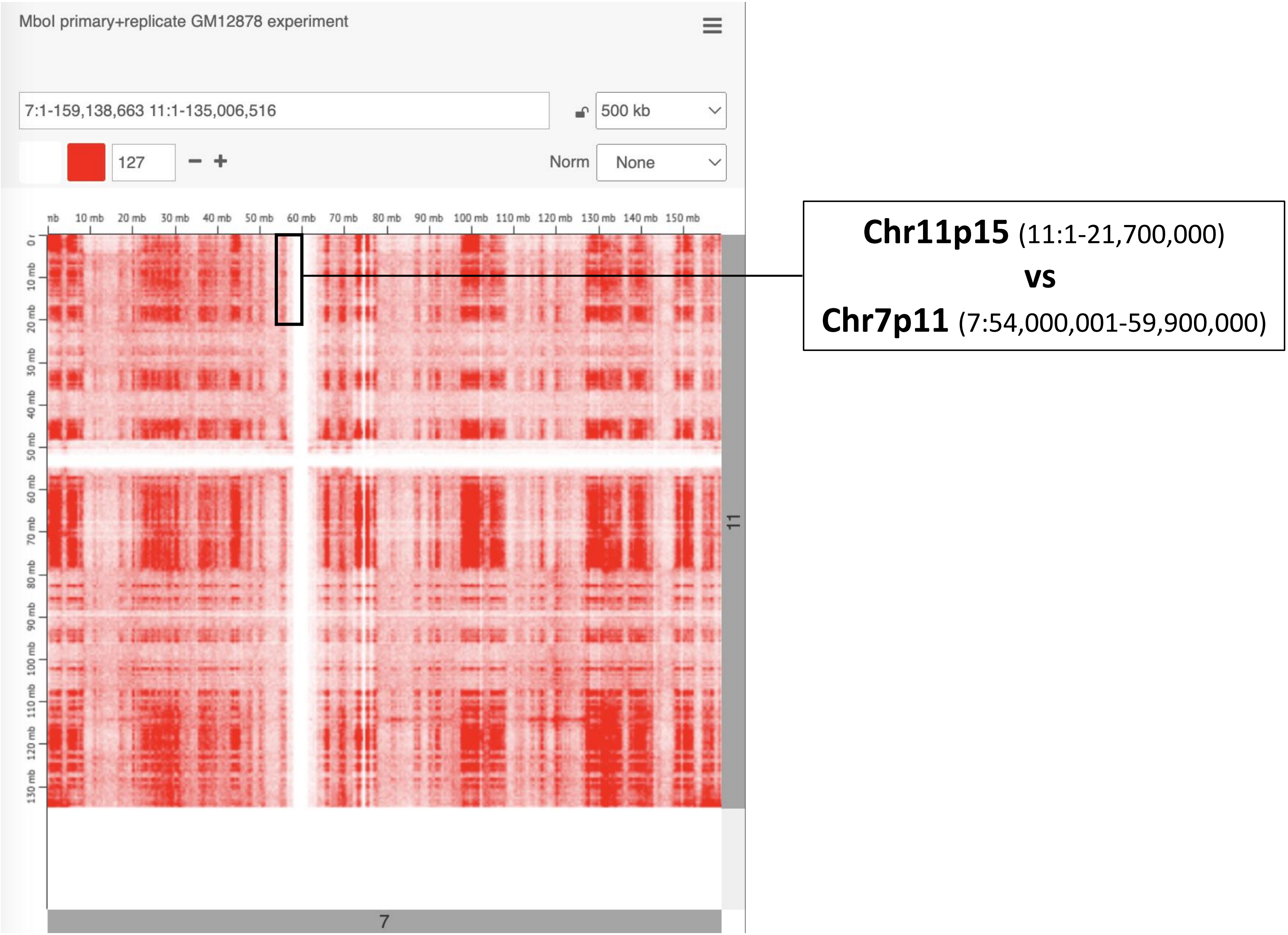
MboI primary+replicate GM12878 experiment
7:1-159,138,663 11:1-135,006,516
500 kb
127
Norm
None
10 mb 20 mb 30 mb 40 mb 50 mb 60 mb 70 mb 80 mb 90 mb 100 mb 110 mb 120 mb 130 mb 140 mb 150 mb
10 mb 20 mb 30 mb 40 mb 50 mb 60 mb 70 mb 80 mb 90 mb 100 mb 110 mb 120 mb 130 mb
11
7
Chr11p15 (11:1-21,700,000)
vs
Chr7p11 (7:54,000,001-59,900,000)

h

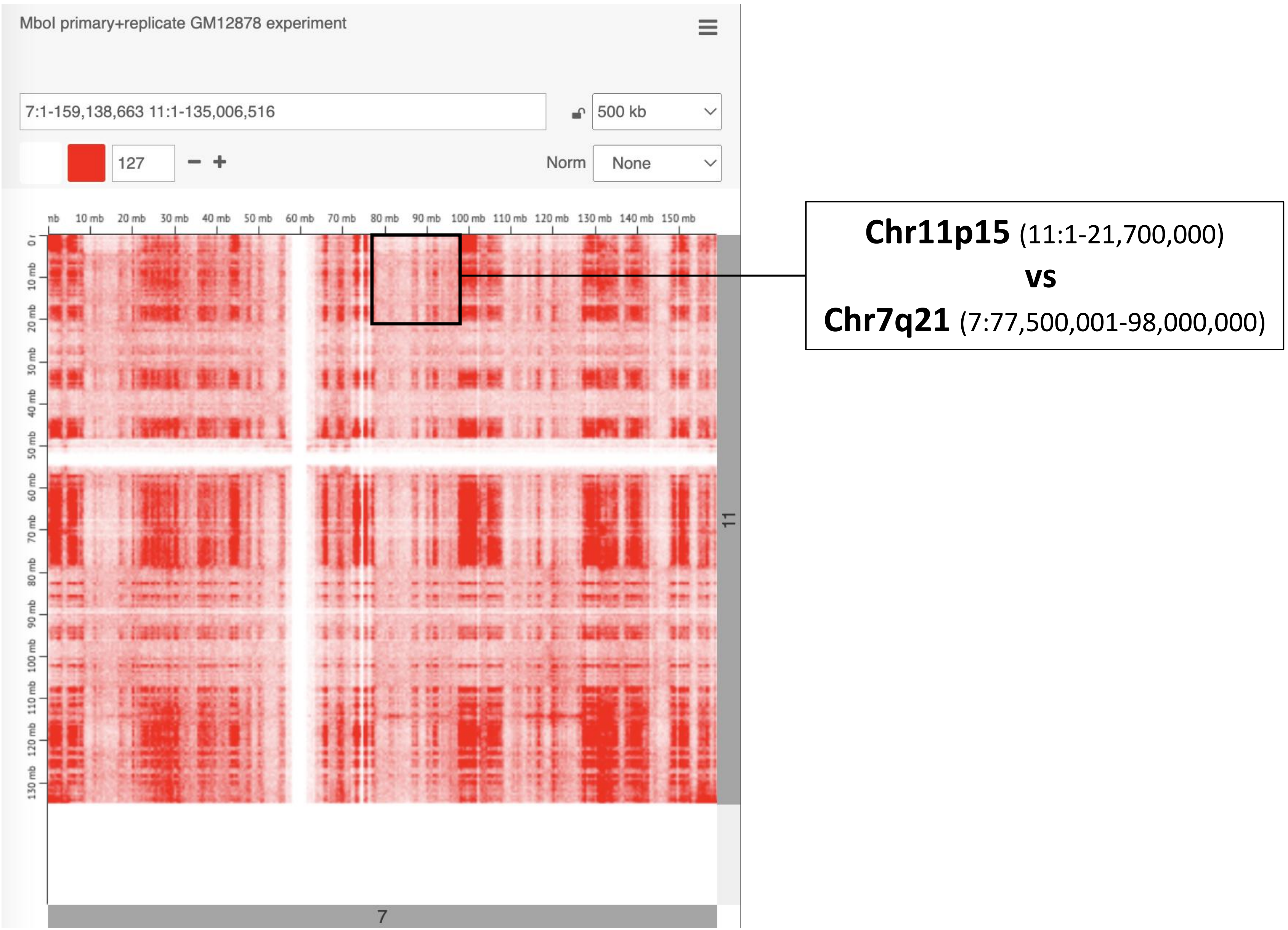

MboI primary+replicate GM12878 experiment
7:1-159,138,663 11:1-135,006,516
500 kb
127
Norm
None
7
11
Chr11p15 (11:1-21,700,000)
vs
Chr7q21 (7:77,500,001-98,000,000)

i

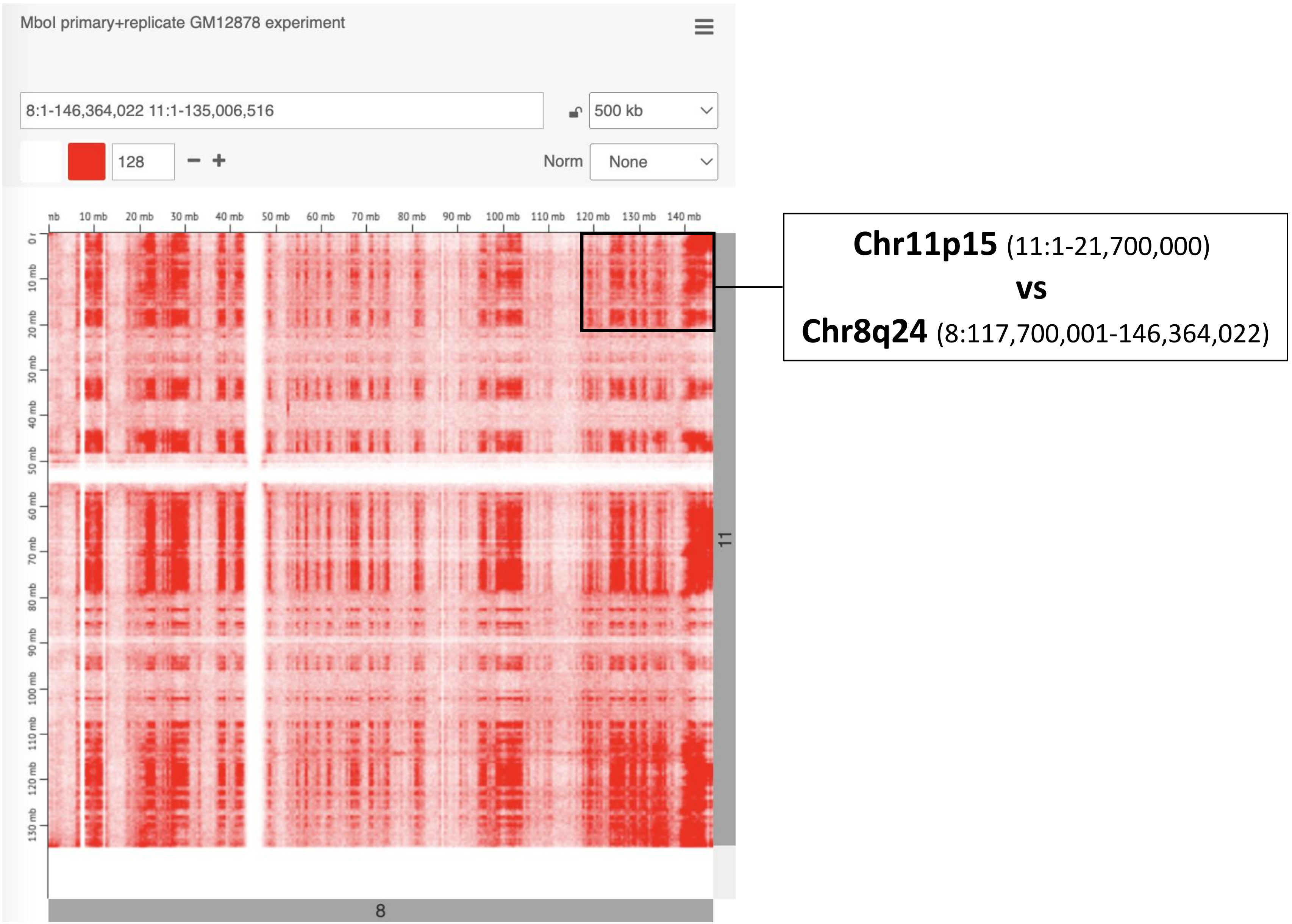
MboI primary+replicate GM12878 experiment
8:1-146,364,022 11:1-135,006,516
500 kb
128
Norm
None
10 mb
20 mb
30 mb
40 mb
50 mb
60 mb
70 mb
80 mb
90 mb
100 mb
110 mb
120 mb
130 mb
140 mb
11
8
Chr11p15 (11:1-21,700,000)
vs
Chr8q24 (8:117,700,001-146,364,022)

j

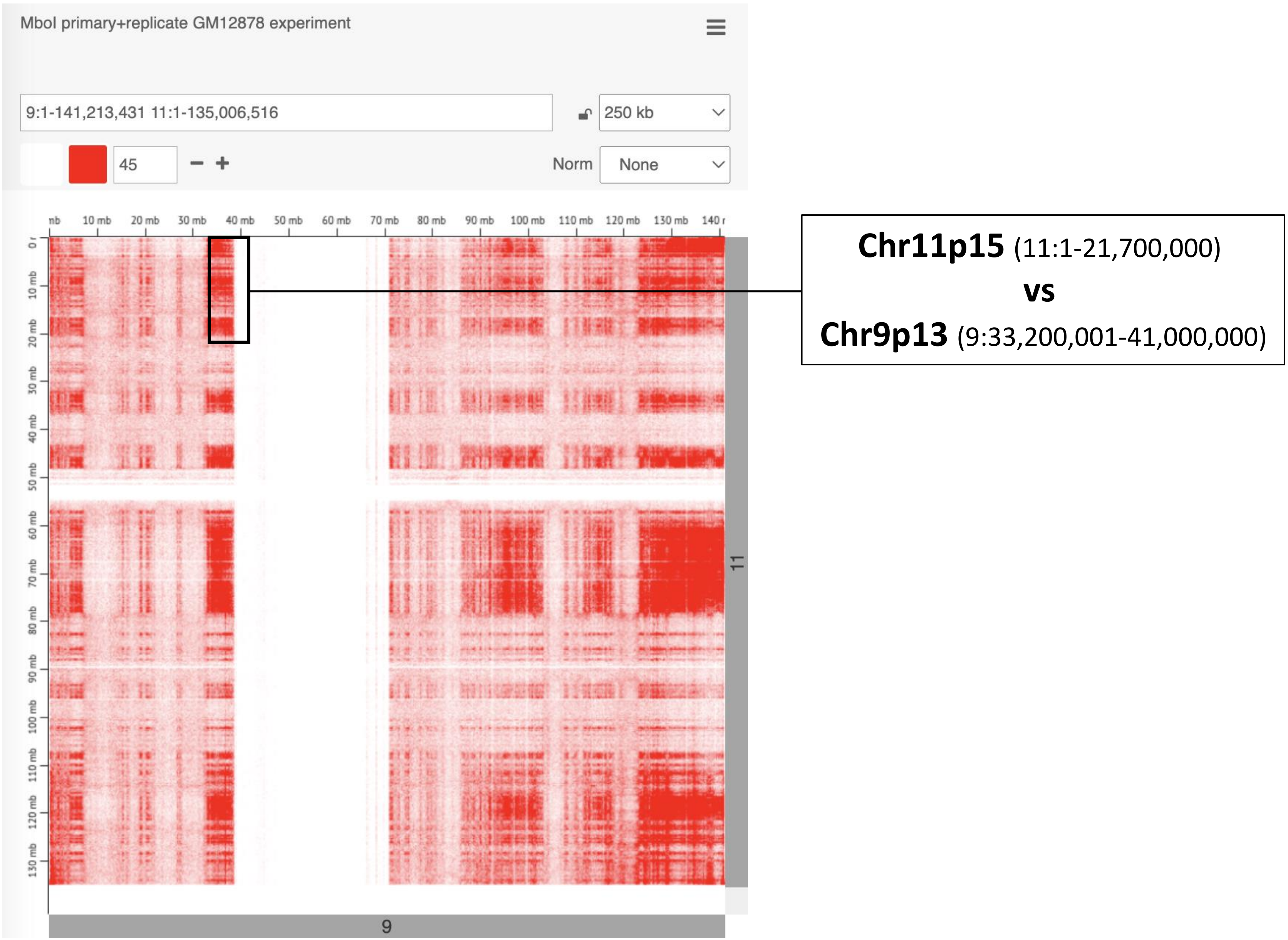

MboI primary+replicate GM12878 experiment
9:1-141,213,431 11:1-135,006,516
250 kb
45
Norm
None
10 mb
20 mb
30 mb
40 mb
50 mb
60 mb
70 mb
80 mb
90 mb
100 mb
110 mb
120 mb
130 mb
11
9
Chr11p15 (11:1-21,700,000)
vs
Chr9p13 (9:33,200,001-41,000,000)

k

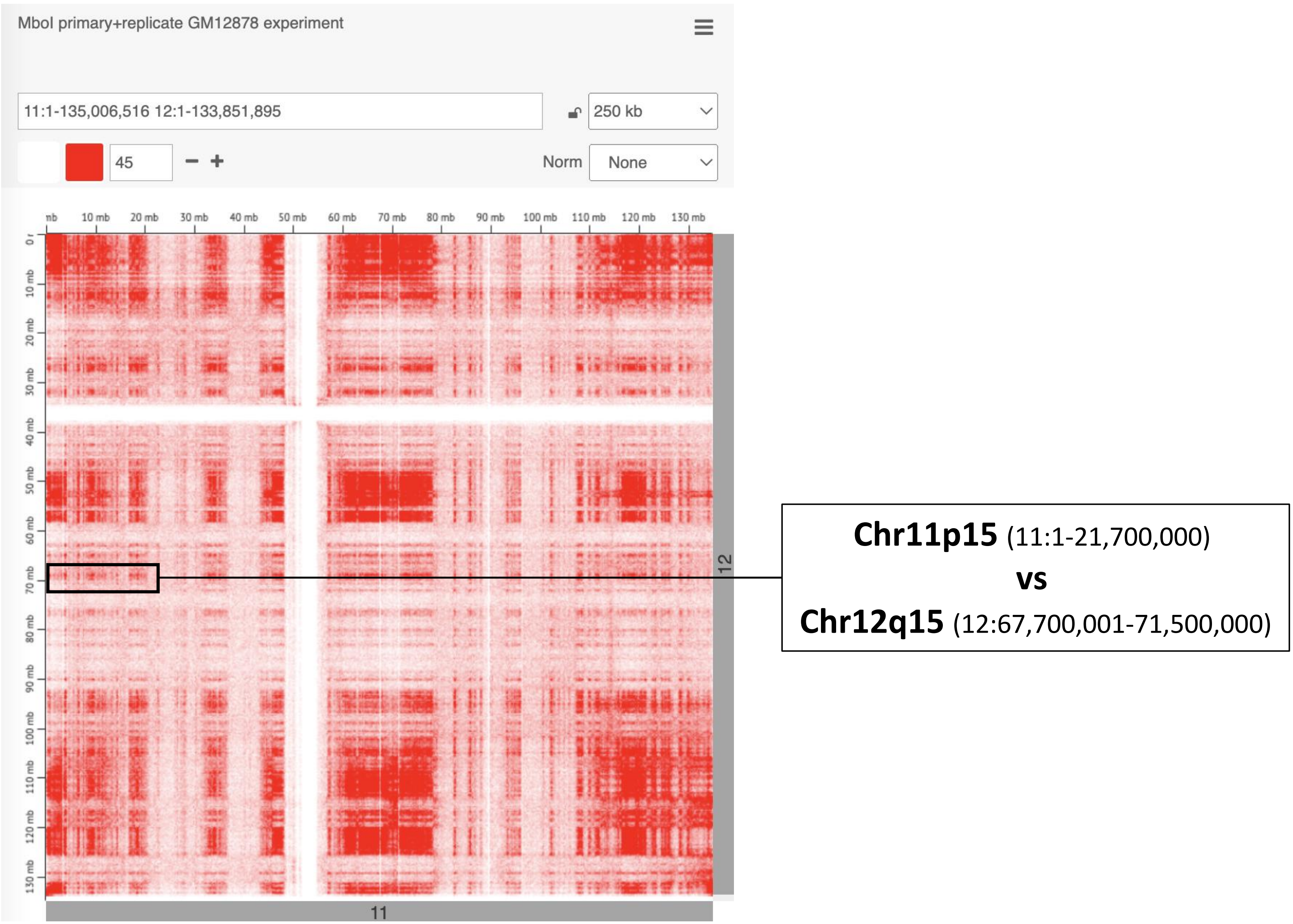

MboI primary+replicate GM12878 experiment
11:1-135,006,516 12:1-133,851,895
250 kb
45
Norm
None
10 mb
20 mb
30 mb
40 mb
50 mb
60 mb
70 mb
80 mb
90 mb
100 mb
110 mb
120 mb
130 mb
12
11
Chr11p15 (11:1-21,700,000)
vs
Chr12q15 (12:67,700,001-71,500,000)

I

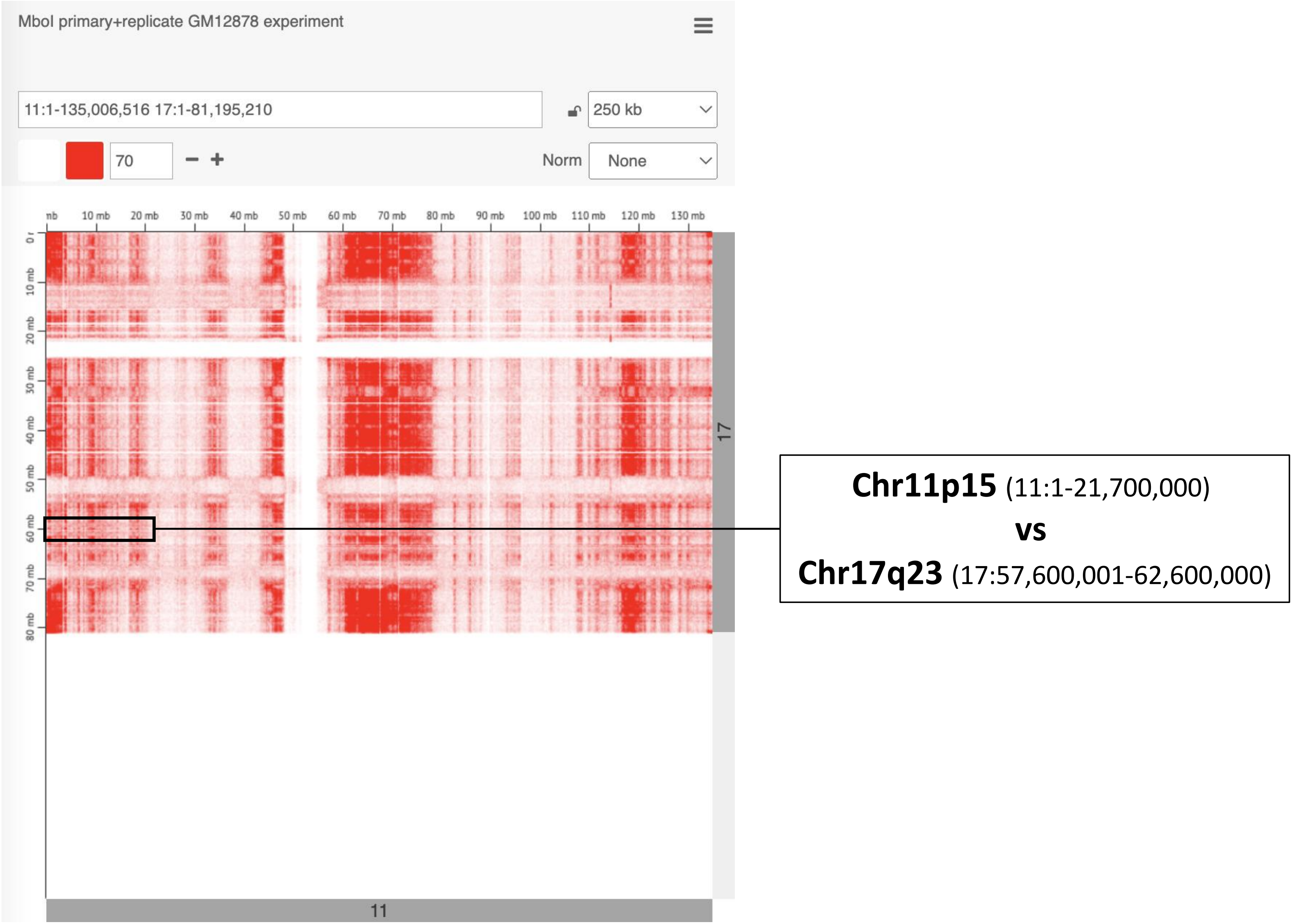
MboI primary+replicate GM12878 experiment
11:1-135,006,516 17:1-81,195,210
250 kb
70
Norm
None
10 mb
20 mb
30 mb
40 mb
50 mb
60 mb
70 mb
80 mb
90 mb
100 mb
110 mb
120 mb
130 mb
17
11
Chr11p15 (11:1-21,700,000)
vs
Chr17q23 (17:57,600,001-62,600,000)

m

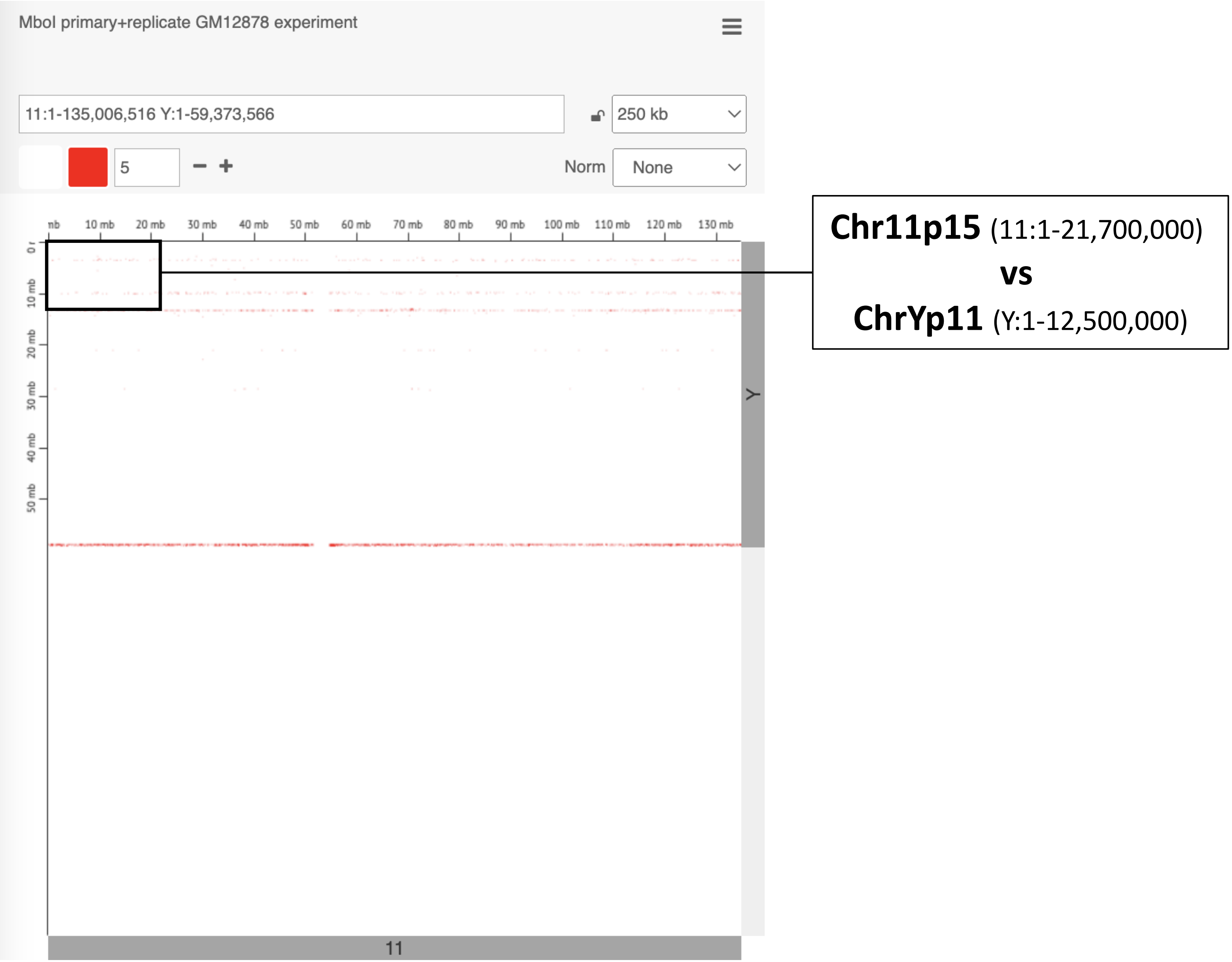
MboI primary+replicate GM12878 experiment
11:1-135,006,516 Y:1-59,373,566
250 kb
5
Norm
None
10 mb
20 mb
30 mb
40 mb
50 mb
60 mb
70 mb
80 mb
90 mb
100 mb
110 mb
120 mb
130 mb
Y
11
Chr11p15 (11:1-21,700,000)
vs
ChrYp11 (Y:1-12,500,000)

n

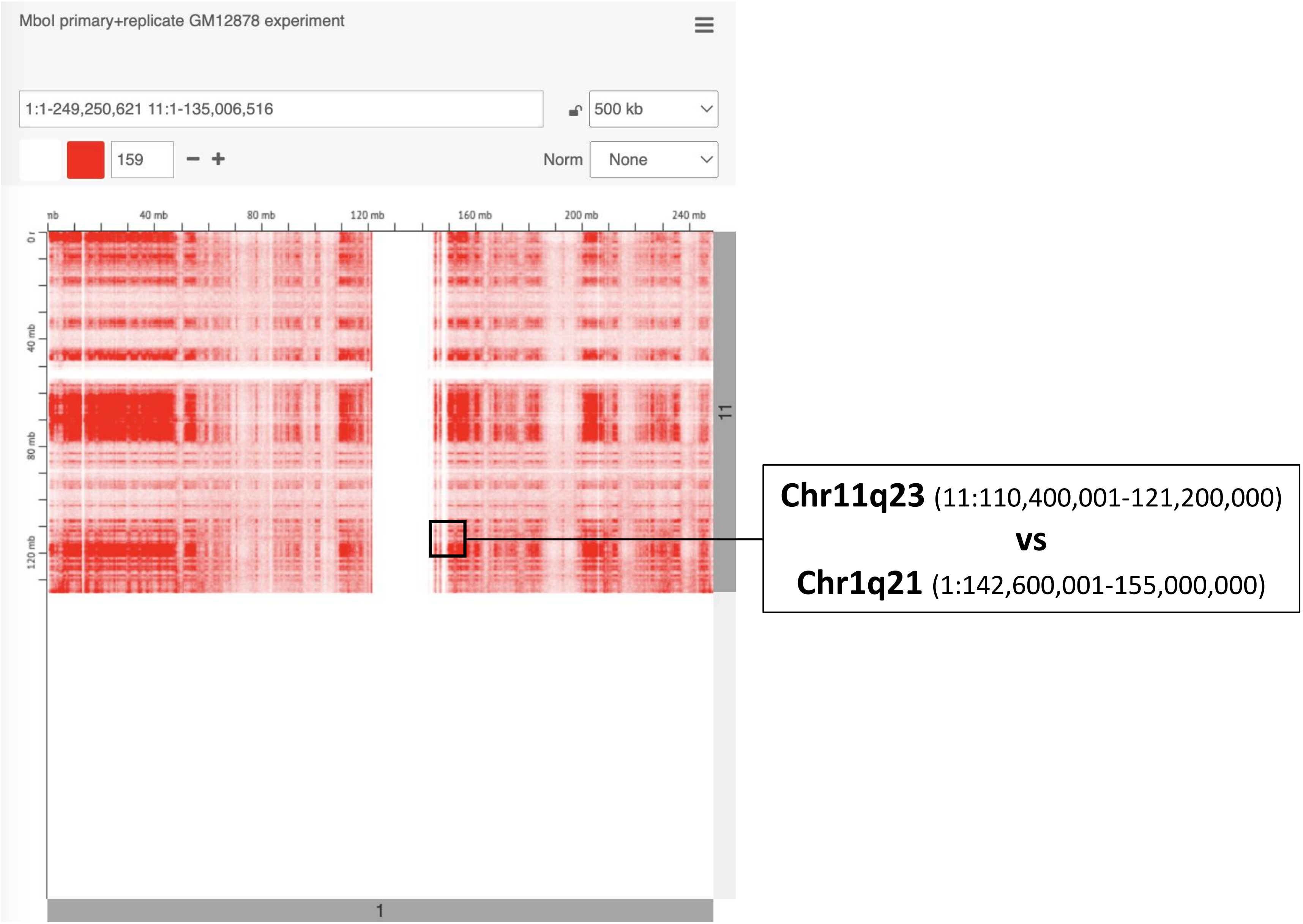
MboI primary+replicate GM12878 experiment
1:1-249,250,621 11:1-135,006,516
500 kb
159
Norm
None
40 mb
80 mb
120 mb
160 mb
200 mb
240 mb
11
1
Chr11q23 (11:110,400,001-121,200,000)
vs
Chr1q21 (1:142,600,001-155,000,000)

o

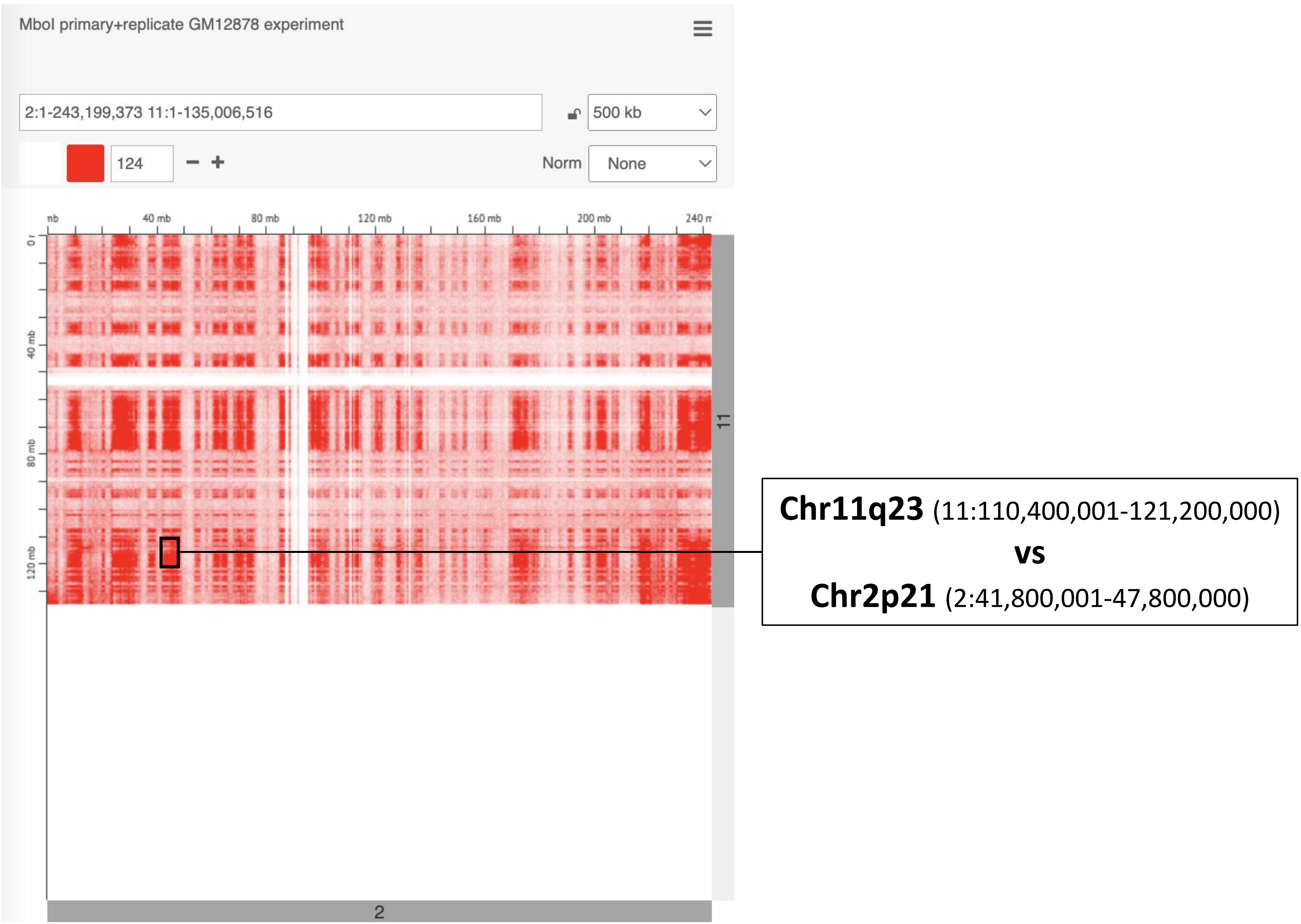

MboI primary+replicate GM12878 experiment
2:1-243,199,373 11:1-135,006,516
500 kb
124
Norm
None
40 mb
80 mb
120 mb
160 mb
200 mb
40 mb
80 mb
120 mb
11
2
Chr11q23 (11:110,400,001-121,200,000)
vs
Chr2p21 (2:41,800,001-47,800,000)

p

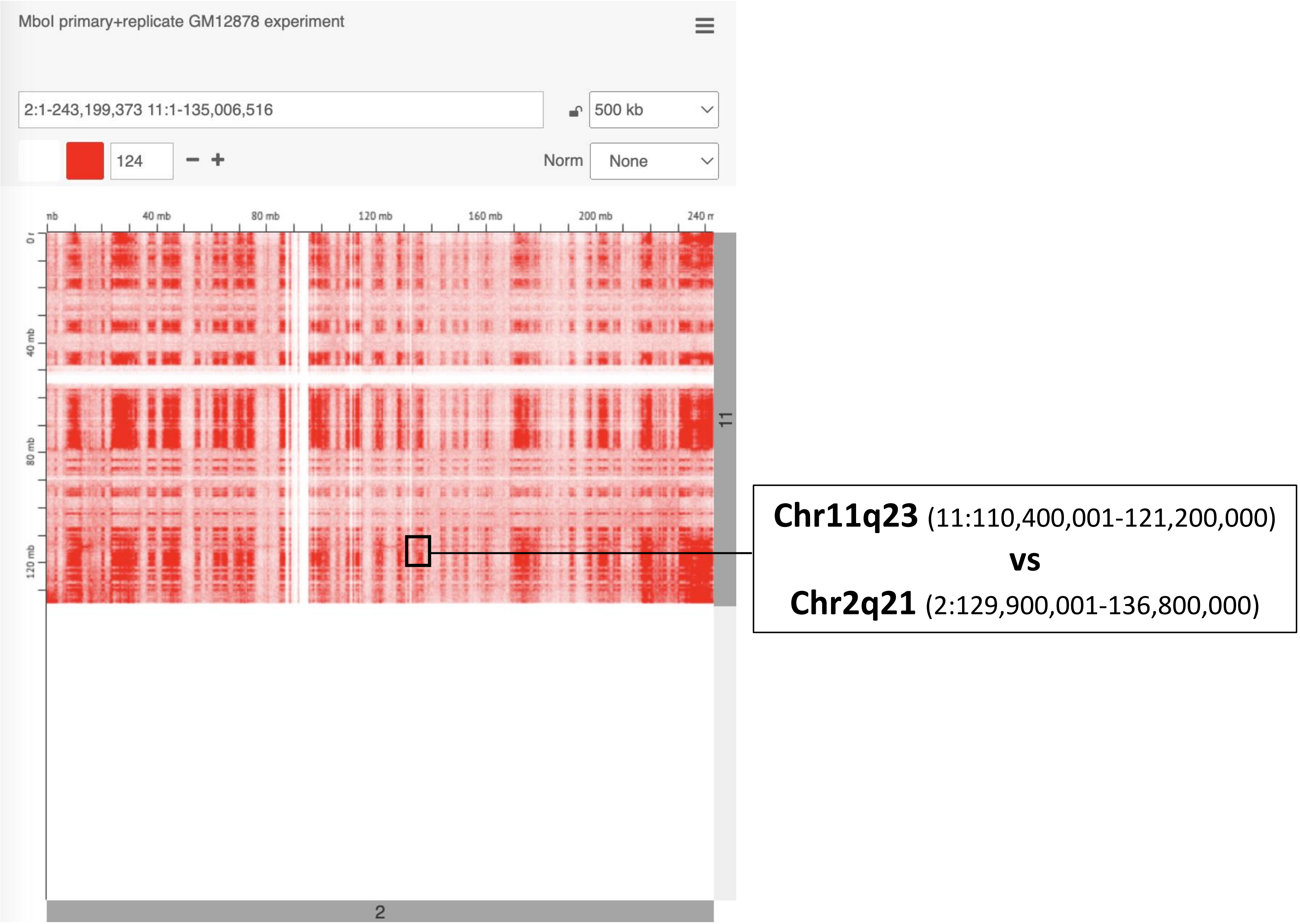
MboI primary+replicate GM12878 experiment
2:1-243,199,373 11:1-135,006,516
500 kb
124
Norm
None
40 mb
80 mb
120 mb
160 mb
200 mb
11
2
Chr11q23 (11:110,400,001-121,200,000)
vs
Chr2q21 (2:129,900,001-136,800,000)

q

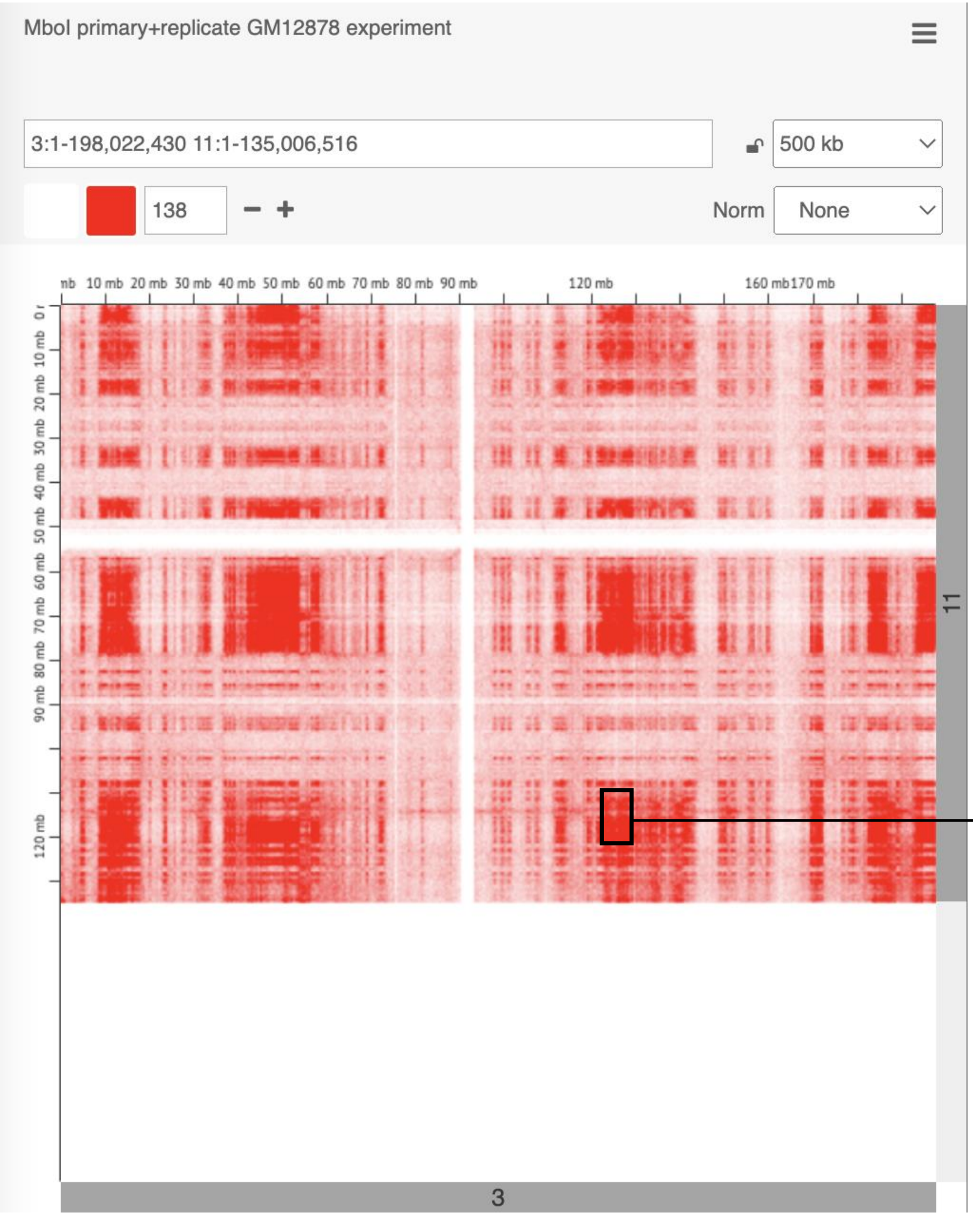


**Chr11q23** (11:110,400,001-121,200,000)

**vs**

**Chr3q21** (3:121,900,001-129,200,000)

r

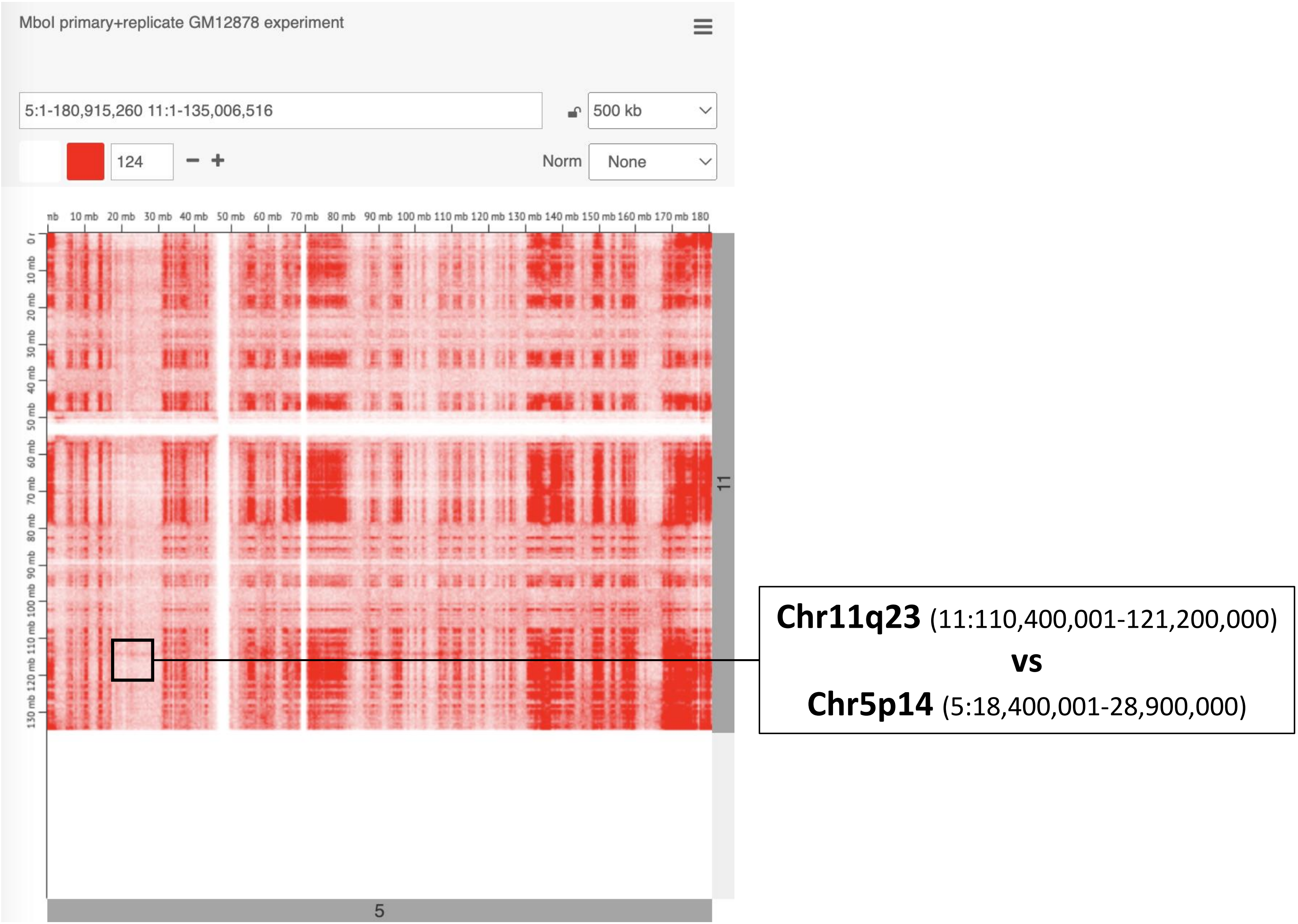

MboI primary+replicate GM12878 experiment
5:1-180,915,260 11:1-135,006,516
500 kb
124
Norm
None
5
11
Chr11q23 (11:110,400,001-121,200,000)
vs
Chr5p14 (5:18,400,001-28,900,000)

S

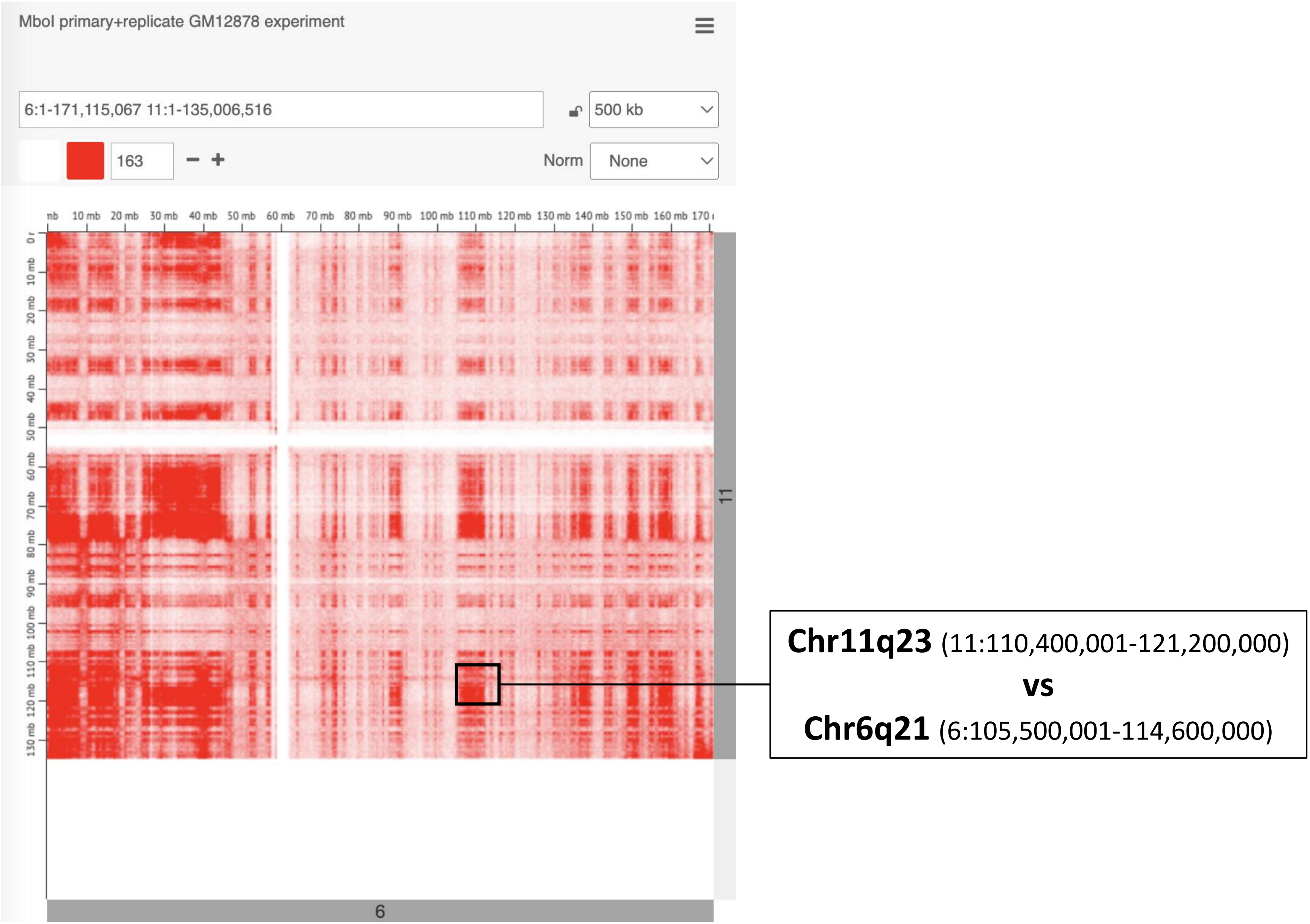
MboI primary+replicate GM12878 experiment
6:1-171,115,067 11:1-135,006,516
500 kb
163
Norm
None
11
6
Chr11q23 (11:110,400,001-121,200,000)
vs
Chr6q21 (6:105,500,001-114,600,000)

t

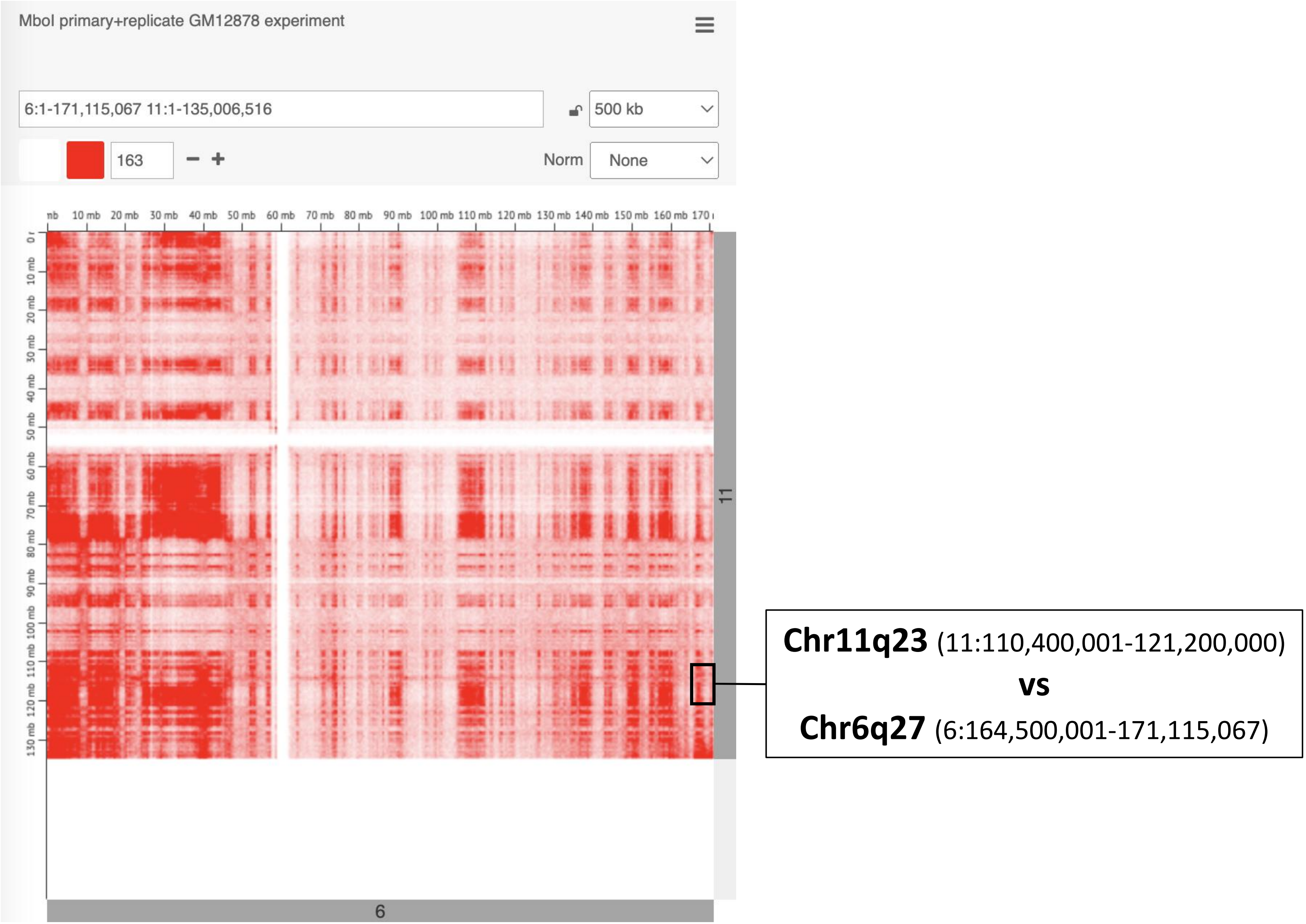
MboI primary+replicate GM12878 experiment
6:1-171,115,067 11:1-135,006,516
500 kb
163
Norm
None
11
6
**Chr11q23** (11:110,400,001-121,200,000)
**vs**
**Chr6q27** (6:164,500,001-171,115,067)

u

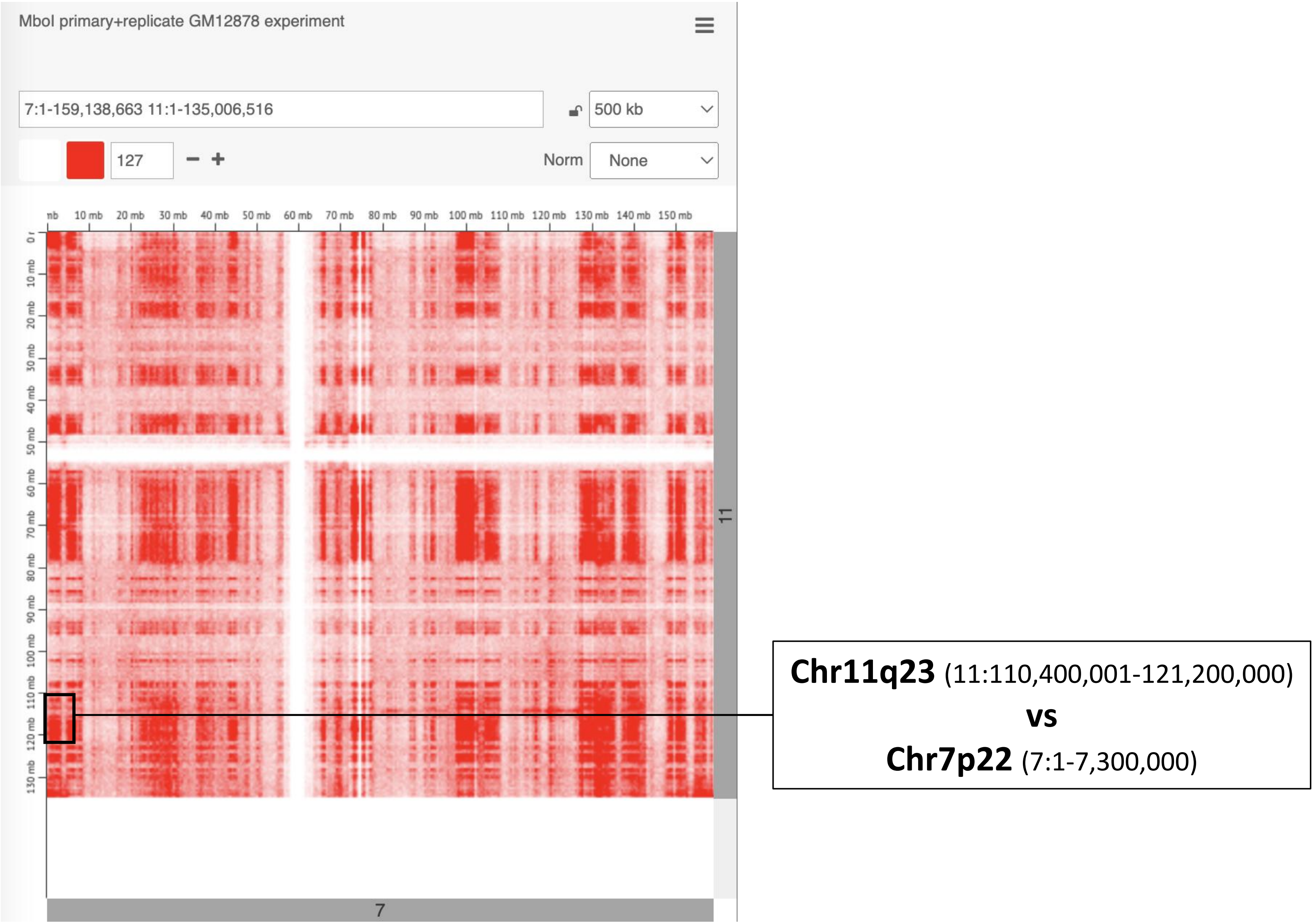
MboI primary+replicate GM12878 experiment
7:1-159,138,663 11:1-135,006,516
500 kb
127
Norm
None
10 mb
20 mb
30 mb
40 mb
50 mb
60 mb
70 mb
80 mb
90 mb
100 mb
110 mb
120 mb
130 mb
140 mb
150 mb
11
7
Chr11q23 (11:110,400,001-121,200,000)
vs
Chr7p22 (7:1-7,300,000)

V

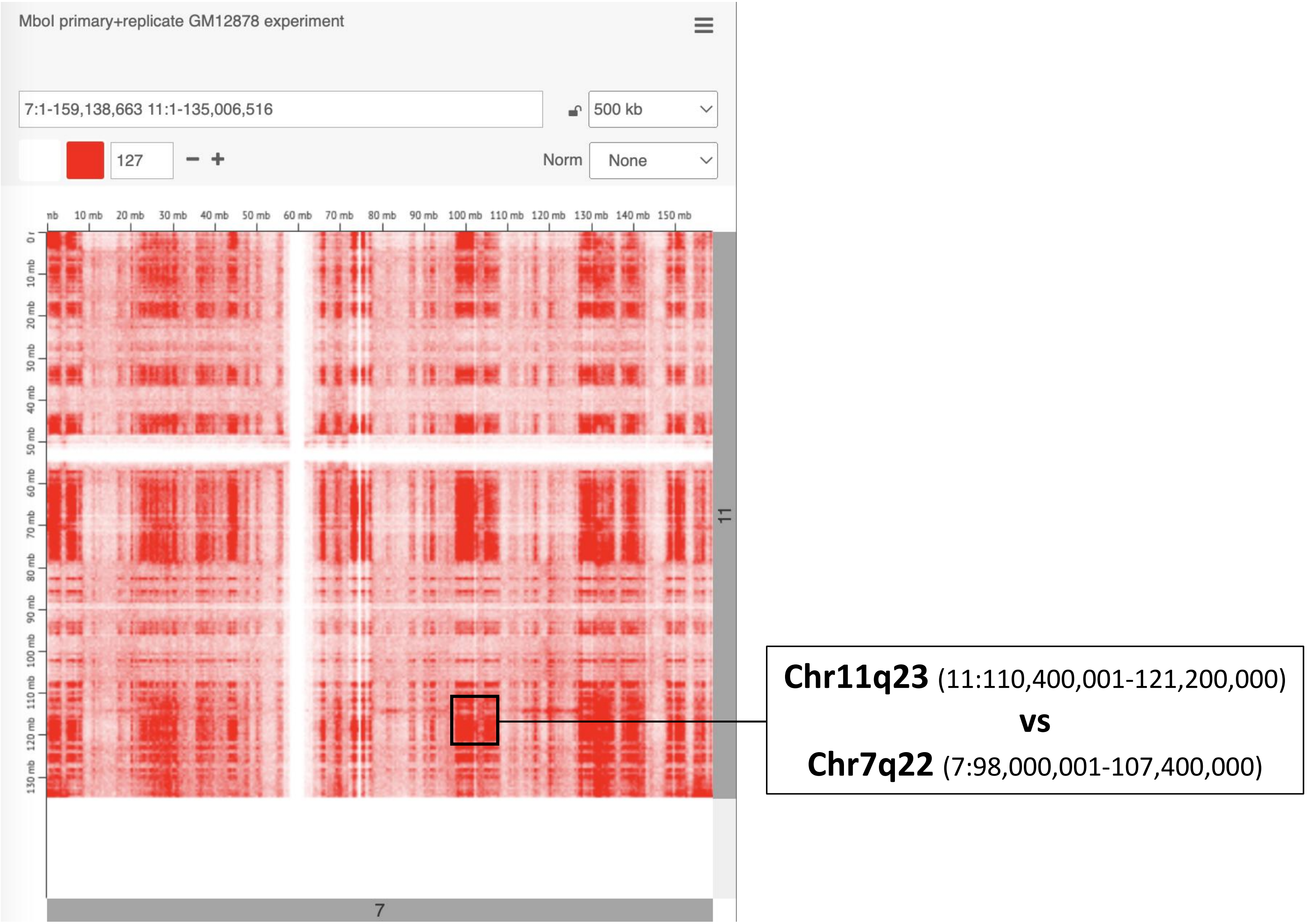
MboI primary+replicate GM12878 experiment
7:1-159,138,663 11:1-135,006,516
500 kb
127
Norm
None
10 mb
20 mb
30 mb
40 mb
50 mb
60 mb
70 mb
80 mb
90 mb
100 mb
110 mb
120 mb
130 mb
140 mb
150 mb
11
7
Chr11q23 (11:110,400,001-121,200,000)
vs
Chr7q22 (7:98,000,001-107,400,000)

w

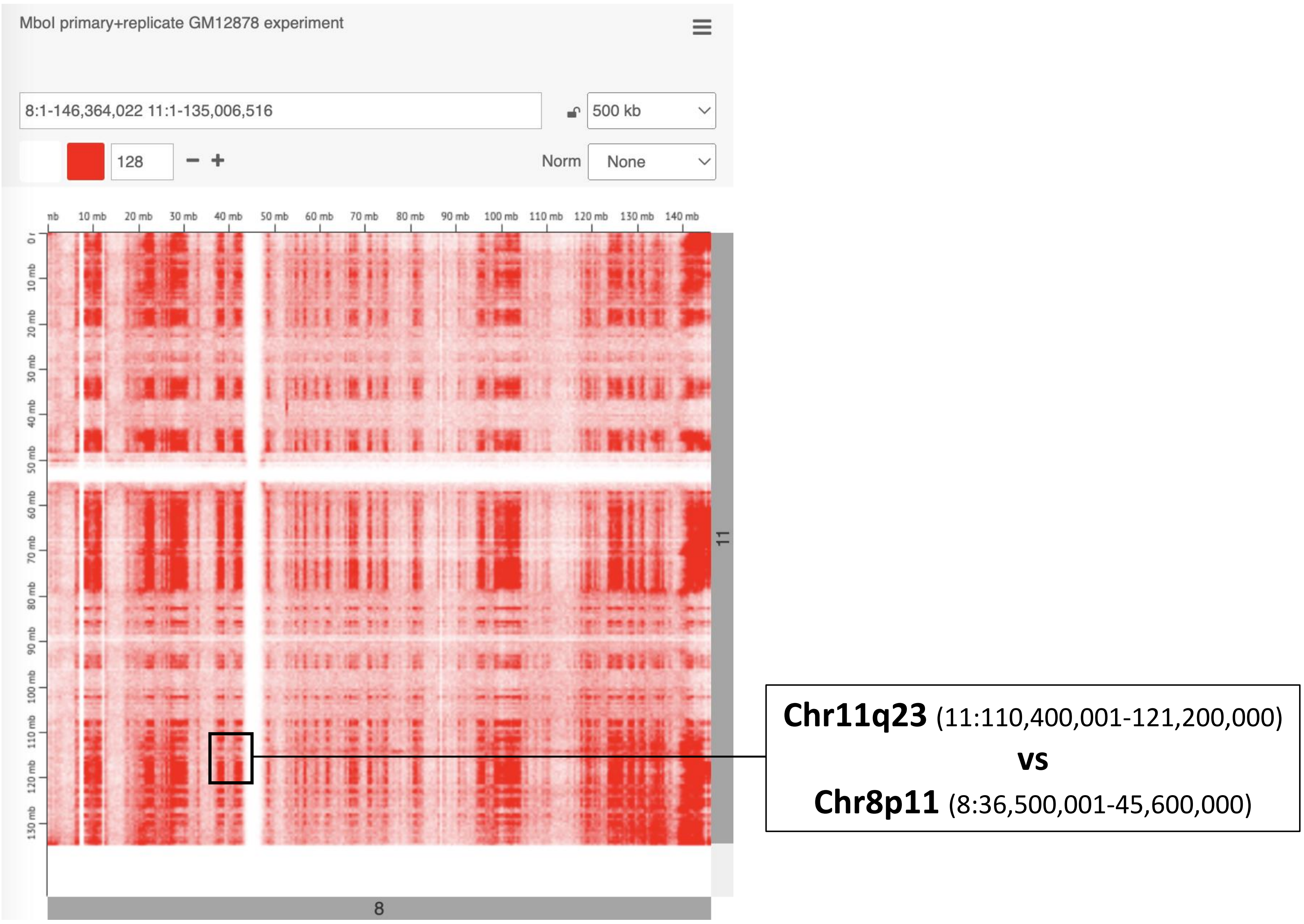

MboI primary+replicate GM12878 experiment
8:1-146,364,022 11:1-135,006,516
500 kb
128
Norm
None
10 mb
20 mb
30 mb
40 mb
50 mb
60 mb
70 mb
80 mb
90 mb
100 mb
110 mb
120 mb
130 mb
140 mb
11
8
Chr11q23 (11:110,400,001-121,200,000)
vs
Chr8p11 (8:36,500,001-45,600,000)

X

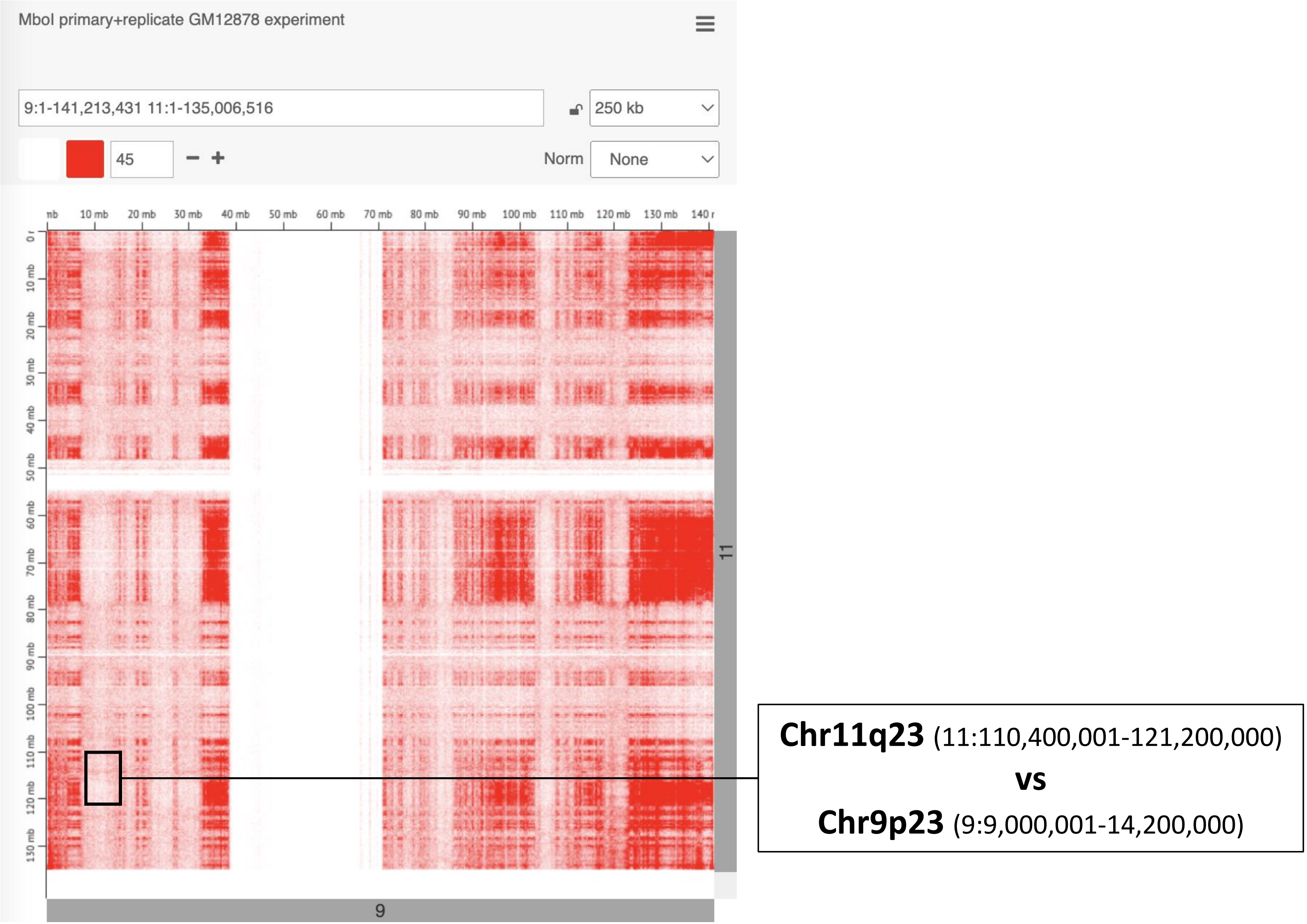
MboI primary+replicate GM12878 experiment
9:1-141,213,431 11:1-135,006,516
250 kb
45
Norm
None
10 mb
20 mb
30 mb
40 mb
50 mb
60 mb
70 mb
80 mb
90 mb
100 mb
110 mb
120 mb
130 mb
9
11
Chr11q23 (11:110,400,001-121,200,000)
vs
Chr9p23 (9:9,000,001-14,200,000)

y

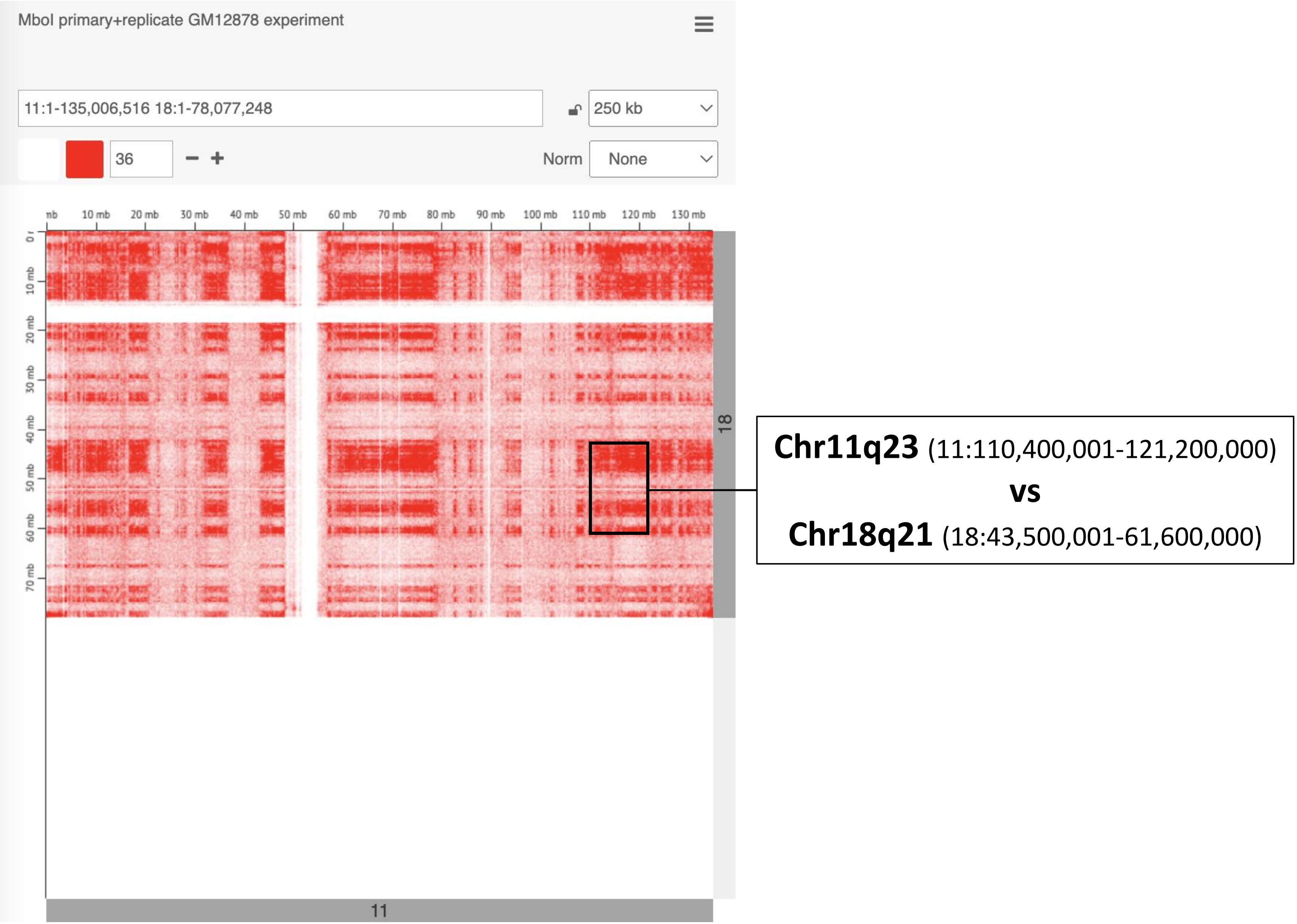
MboI primary+replicate GM12878 experiment
11:1-135,006,516 18:1-78,077,248
250 kb
36
Norm
None
10 mb
20 mb
30 mb
40 mb
50 mb
60 mb
70 mb
80 mb
90 mb
100 mb
110 mb
120 mb
130 mb
18
11
Chr11q23 (11:110,400,001-121,200,000)
vs
Chr18q21 (18:43,500,001-61,600,000)

z

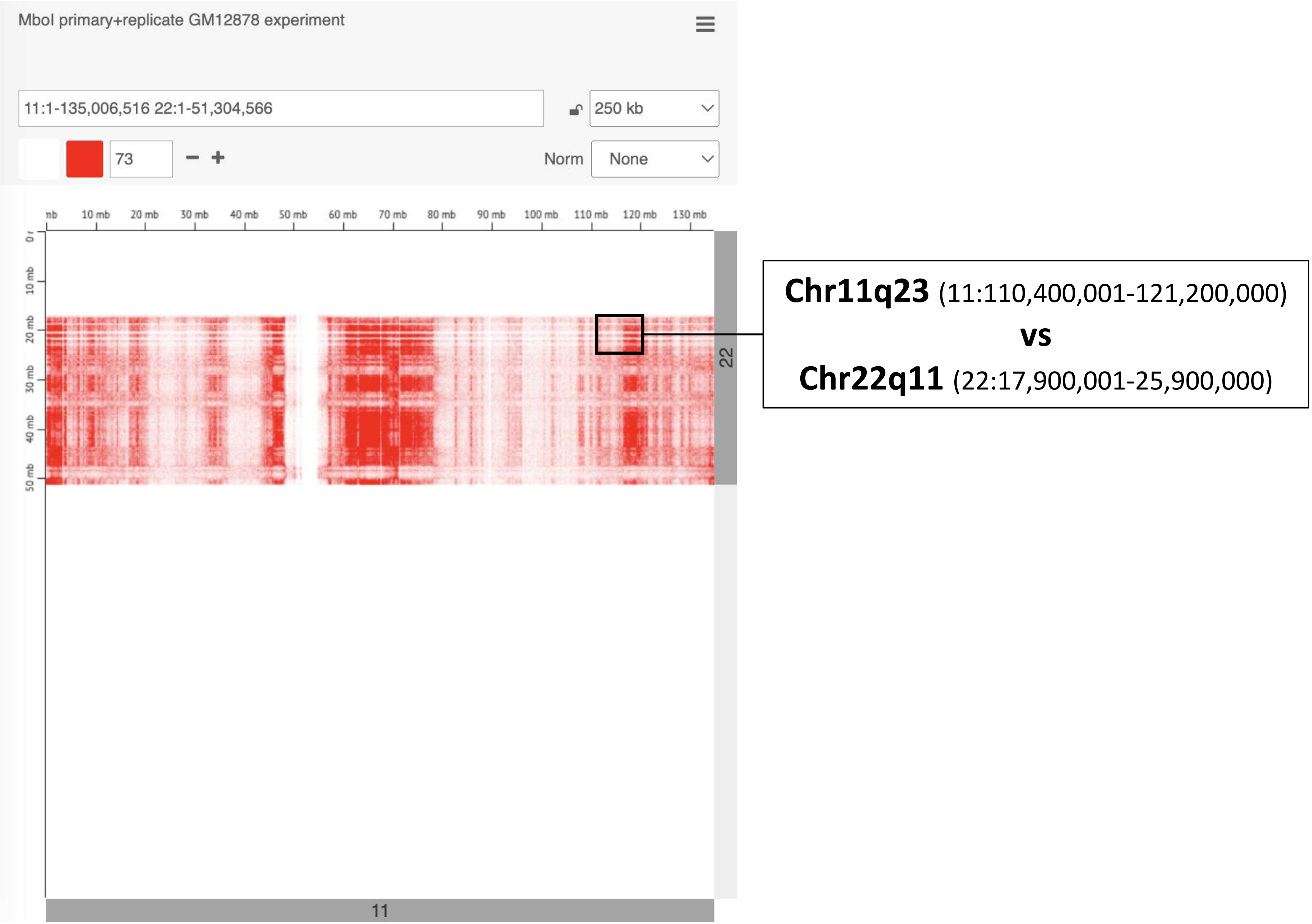

MboI primary+replicate GM12878 experiment
11:1-135,006,516 22:1-51,304,566
250 kb
73
Norm
None
10 mb
20 mb
30 mb
40 mb
50 mb
60 mb
70 mb
80 mb
90 mb
100 mb
110 mb
120 mb
130 mb
22
11
**Chr11q23** (11:110,400,001-121,200,000)
**vs**
**Chr22q11** (22:17,900,001-25,900,000)

**Table S1:** Sex distribution of patients showing chromosome 11 anomalies identified by conventional chromosome analysis.

| Sex | Translocations | Inversions | Insertion | Deletions | Total |
|---|---|---|---|---|---|
| male | 72 | 3 | 0 | 1 | 76 |
| female | 66 | 11 | 1 | 1 | 79 |
| total | 138 | 14 | 1 | 2 | 155 |

**Table S2:** Sex distribution of patients showing chromosome 11 anomalies identified by array-CGH.

| Sex | Deletions | Duplications | Total |
|---|---|---|---|
| male | 23 | 18 | 41 |
| female | 12 | 14 | 26 |
| total | 35 | 32 | 67 |

**Table S3.** Inheritance of translocations.

| chromosome | paternal | maternal | de novo | unknown | total |
|---|---|---|---|---|---|
| 1 | 2 | 1 | 0 | 4 | 7 |
| 2 | 2 | 0 | 1 | 6 | 9 |
| 3 | 0 | 1 | 4 | 1 | 6 |
| 4 | 0 | 2 | 0 | 5 | 7 |
| 5 | 0 | 3 | 1 | 2 | 6 |
| 6 | 0 | 1 | 0 | 4 | 5 |
| 7 | 1 | 2 | 0 | 6 | 9 |
| 8 | 2 | 4 | 0 | 4 | 10 |
| 9 | 1 | 1 | 1 | 2 | 5 |
| 10 | 0 | 2 | 0 | 4 | 6 |
| 12 | 1 | 1 | 1 | 0 | 3 |
| 13 | 0 | 2 | 0 | 1 | 3 |
| 14 | 0 | 0 | 0 | 3 | 3 |
| 15 | 1 | 0 | 0 | 1 | 2 |
| 16 | 0 | 0 | 0 | 1 | 1 |
| 17 | 1 | 1 | 0 | 1 | 3 |
| 18 | 0 | 2 | 1 | 0 | 3 |
| 21 | 2 | 0 | 0 | 0 | 2 |
| 22 | 4 | 16 | 2 | 23 | 45 |
| X | 0 | 0 | 1 | 1 | 2 |
| Y | 0 | 0 | 1 | 0 | 1 |
| total | 17 | 39 | 13 | 69 | 138 |

**Table S4.** Distribution of translocation breakpoints on chromosome 11 partners. Based on Glusman's mapping (see ref. 4 in the main text), OR gene families are present or absent[1].

| Chromosome | Bkps in cytoband with ORs | Bkps in cytoband without ORs | Tot bkps (%) | Chromosome lenght (Mb) | Average number of bkps per 100 Mb |
|---|---|---|---|---|---|
| 1 | 2 | 5 | 7 (5.1) | 249 | 2.8 |
| 2 | 3 | 6 | 9 (6.5) | 243 | 3.7 |
| 3 | 3 | 3 | 6 (4.3) | 198 | 3.0 |
| 4 | 1 | 6 | 7 (5.1) | 191 | 3.7 |
| 5 | 0 | 6 | 6 (4.3) | 181 | 3.3 |
| 6 | 3 | 2 | 5 (3.6) | 171 | 2.9 |
| 7 | 1 | 8 | 9 (6.5) | 159 | 5.7 |
| 8 | 0 | 10 | 10 (7.2) | 146 | 6.8 |
| 9 | 2 | 3 | 5 (3.6) | 141 | 3.5 |
| 10 | 1 | 5 | 6 (4.3) | 136 | 4.4 |
| 12 | 0 | 3 | 3 (2.2) | 134 | 2.2 |
| 13 | 1 | 2 | 3 (2.2) | 115 | 2.6 |
| 14 | 2 | 1 | 3 (2.2) | 107 | 2.8 |
| 15 | 1 | 1 | 2 (1.4) | 103 | 1.9 |
| 16 | 0 | 1 | 1 (0.7) | 90 | 1.1 |
| 17 | 3 | 0 | 3 (2.2) | 81 | 3.7 |
| 18 | 1 | 2 | 3 (2.2) | 78 | 3.85 |
| 19 | 0 | 0 | 0 | 59 | 0.0 |
| 20 | 0 | 0 | 0 | 63 | 0.0 |
| 21 | 2 | 0 | 2 (0.7) | 48 | 4.2 |
| 22 | 0 | 45 | 45 (32.6) | 51 | 88.2 |
| X | 0 | 2 | 2 (1.4) | 155 | 1.3 |
| Y | 0 | 1 | 1 (0.7) | 59 | 1.7 |
| total | 26 (18.8%) | 112 (81.2%) | 138 | | |

Bkps: breakpoints

**Table S5. Cytobands involved in the main collected translocations, visualized on the Hi-C maps**

| Chromosome 11 cytoband (chromosome coordinates, hg19) | Partner chromosome cytoband (chromosome coordinates, hg19) | Figure S5 panel showing interchromosomal interactions |
|---|---|---|
| Chr11p15 (11:1-21,700,000) | Chr2q31 (2:169,700,001-183,000,000) | a |
| | Chr4p14 (4:35,800,001-41,200,000) | b |
| | Chr5p15 (5:1-18,400,000) | c |
| | Chr5q31 (5:130,600,001-144,500,000) | d |
| | Chr6p23 (6:13,400,001-15,200,000) | e |
| | Chr6q16 (6:93,100,001-105,000,000) | f |
| | Chr7p11 (7:54,000,001-59,900,000) | g |
| | Chr7q21 (7:77,500,001-98,000,000) | h |
| | Chr8q24 (8:117,700,001-146,364,022) | i |
| | Chr9p13 (9:33,200,001-41,000,000) | j |
| | Chr12q15 (12:67,700,001-71,500,000) | k |
| | Chr17q23 (17:57,600,001-62,600,000) | l |
| | ChrYp11 (Y:1-12,500,000) | m |
| Chr11q23 (11:110,400,001-121,200,000) | Chr1q21 (1:142,600,001-155,000,000) | n |
| | Chr2p21 (2:41,800,001-47,800,000) | o |
| | Chr2q21 (2:129,900,001-136,800,000) | p |
| | Chr3q21 (3:121,900,001-129,200,000) | q |
| | Chr5p14 (5:18,400,001-28,900,000) | r |
| | Chr6q21 (6:105,500,001-114,600,000) | s |
| | Chr6q27 (6:164,500,001-171,115,067) | t |
| | Chr7p22 (7:1-7,300,000) | u |
| | Chr7q22 (7:98,000,001-107,400,000) | v |
| | Chr8p11 (8:36,500,001-45,600,000) | w |
| | Chr9p23 (9:9,000,001-14,200,000 | x |
| | Chr18q21 (18:43,500,001-61,600,000) | y |
| | Chr22q11 (22:17,900,001-25,900,000) | z |

**Supplemental Methods.**

1) **Array Comparative Genomic Hybridization (Array-CGH)**

The *Cytogenetics, Molecular Genetics and Medical Genetics Unit, Toma Advanced Biomedical Assays* used several platform of analysis.

1) Samples until 2016. A genome-wide BAC platform (ConstitutionalChip 4.0, PerkinElmer Wallac, Turku, Finland) with a dye-swap approach. The average spatial resolution is 600 Kbp and the reference DNAs were commercial pools of human male or female DNAs (Promega Corporation, Madison, Wisconsin, USA). The referred samples were analysed using a dye-swap experiment by reversal dye approach hybridizing the sample DNA against a sex matched human commercial DNA reference (Promega). Data were analysed with the OneClickCGH 4.3.3 Software (PerkinElmer).
2) Samples from 2016 to 2020. Post-natal samples were run on a 135K-feature, whole-genome oligonucleotide-based microarray (Roche NimbleGen, Madison, Wis., USA). Data were analysed and displayed with Genoglyphix analysis software 2.6 (Signature Genomics, a subsidiary of PerkinElmer, Inc., Spokane, Wash., USA). Pre-natal samples were run on an oligo genome-wide CGX Array 37K (PerkinElmer). Resolution: in critical regions 1 probe/10Kb, in backbone 1 probe/100Kb. Design: whole-genome oligonucleotide-based microarray analysing the backbone and more than 980 regions including >200 critical regions for known microdeletion/microduplication syndromes, 41 subtelomeres, 43 pericentromeric regions, >200 transcription factors and >200 developmental genes. Genoglyphix 3.0 software (Signature Genomics, PerkinElmer) is set to use a segmentation algorithm flagging segments of copy-number gain or loss involving a minimum of 5 consecutive probes and a log2 of the normalized ratio of the sample: control signal intensities (log2 ratio) of ±0.3 which provides an average effective resolution of 40Kb in the critical region and 400Kb in the backbone.
3) Samples from 2020 onwards. Post-natal samples were run on an oligo genome-wide GenetiSure Cyto 4x180K CGH+SNP (Agilent). Labelling of genomic DNA and hybridization is conducted by aCGH kit (Agilent). Microarray analysis is performed by Cytogenomics software v5.0.2.5 (Agilent). The platform analyses 3644 genes, with the following resolution: 1 probe/7.3 kb in critical regions, 1 probe/57.1 kb in backbone, 1/25 kb in subtelomeric region and 1/10.5 Kb in PAR region. The software is set to use ADM2 algorithm flagging segments of copy-number gain or loss involving a minimum of 5 consecutive probes with an average effective resolution of 29.2 kb in the critical regions and 228.4 kb in the backbone and 8Mb for LOH. Pre-natal samples were run on an oligo genome-wide GenetiSure Cyto 8x60K CGH (Agilent). Labelling of genomic DNA and hybridization is conducted by aCGH kit (Agilent). Microarray analysis is performed by Cytogenomics software v5.1.2.1 (Agilent). The platform analyses 3644 genes, with the following resolution: 1 probe every 7.1 kb in critical regions, 1 every 67.4 kb in backbone, 1 every 31.8 kb in subtelomeric region and 1 every 13.5 Kb in PAR regions. The software is set to use ADM2 algorithm flagging segments of copy-number gain or loss involving a minimum of 5 consecutive probes with an average effective resolution of 28.4 kb in the critical regions and 269.6 kb in the backbone.

2) **Juicebox Aiden Lab Tool** (http://www.aidenlab.org/juicebox/), a Hi-C data visualization software, was used to investigate the possible proximity of the chromosomal territories between chromosome 11 and its partner chromosomes involved in the collected translocations. Experiment ENCSR410MDC (ENCFF718AWL), with reference assembly hg19, carried out on Homo sapiens GM12878 cell line and whose fastq raw data files had been deposited in ENCODE by Erez, Aiden and Baylor Lab, was selected as reference dataset. The interaction regions between each of the two main cytobands of chromosome 11 involved in the translocations (11p15 and 11q23) and those of their respective partner chromosomes were visualized on the Hi-C maps (Table S5 and Figure S4). Genomic coordinates of the cytobands were obtained from UCSC Genome Browser on Human (GRCh37/hg19) (https://genome.ucsc.edu/). We observed territorial proximity for several of the cytobands involved in the translocations, as indicated by the deep red staining of their interactions on the Hi-C heat maps (Figure S5 a, b, d, e, i, j, n, o, p, q, s, t, u, v, w, y).
